# Reprogramming Lung Redox Homeostasis by NIR Driven Ultra‐Small Pd Loaded Covalent Organic Framework Inhibits NF‐κB Pathway for Acute Lung Injury Immunotherapy

**DOI:** 10.1002/advs.202413697

**Published:** 2025-02-18

**Authors:** Doudou Lei, Lin Liao, Tao Qin, Xiaoxuan Guan, Kunpeng Duan, Zhiwei Gao, Weiqian Jin, Mingjing Yin, Ke Zhang, Yan Liu, Yin Chen, Huyang Gao, Jiaxiao Li, Feifei Huang, Wenjing Liu, Chengde Xia, Bailei Wang, Hualin Huang, Shengqiu Lv, Qiang Zhi, Jiahao Huang, Ming Gao, Junyu Lu

**Affiliations:** ^1^ Intensive Care Unit The Second Affiliated Hospital of Guangxi Medical University Nanning Guangxi 530 007 China; ^2^ Department of Clinical Laboratory Key Laboratory of Clinical Laboratory Medicine of Guangxi Department of Education The First Affiliated Hospital of Guangxi Medical University Nanning Guangxi 530 021 China; ^3^ Department of Emergency Guangxi Medical University Cancer Hospital Nanning Guangxi 530 021 China; ^4^ Life Sciences Institute Guangxi Medical University Nanning Guangxi 530 021 China; ^5^ Department of Colorectal and Anal Surgery Department of Emergency The First Affiliated Hospital of Guangxi Medical University Nanning Guangxi 530 021 China; ^6^ Plastic Surgery The Second Affiliated Hospital of Nanchang University Nanchang Jiangxi 330 006 China; ^7^ Department of Burns The First People's Hospital of Zhengzhou Zhengzhou 450 004 China; ^8^ Department of Critical Care Medicine The Ninth Affiliated Hospital of Guangxi Medical University Beihai 536 000 China

**Keywords:** acute lung injury, covalent organic framework, M2 directional polarization, NF‐κB signaling pathway, ROS scavenging

## Abstract

Acute lung injury (ALI) refers to damage to lung related cells, typically caused by an uncontrollable inflammatory response, and over‐generated reactive oxygen species (ROS). Increasing evidence suggests that reprogramming lung redox homeostasis holds significant potentials for the clinical treatment of ALI. Herein, the simple synthesis of ultra‐small Pd loaded covalent organic framework (COF) (TP@Pd) is reported, which, when combined with near infrared (NIR) irradiation, exhibits nanozyme functionalities, including multiple enzyme mimicking activities and broad spectrum ROS scavenging, thereby promoting tissue repair for ALI immunotherapy. Mechanistically, through the therapeutic strategy of TP@Pd+NIR, the damaged cells and tissues are ameliorated by decreasing intracellular ROS levels (total ROS, ·OH and ·O_2_^−^), downregulating inflammatory cytokines levels (IL‐6, TNF‐α and IL‐1β), upregulating antioxidant factor level (SOD2), inducing macrophage M2 directional polarization (downregulation of iNOS and CD86, and upregulation of IL‐10 and CD206), activating immunoregulation (CD4^+^/CD8^+^ ratio increase), promoting tissue repair factor levels (upregulation of HSP70 and CD31), and suppressing the NF‐κB signaling pathway (downregulation of phosphorylated p65 and IκBα). Furthermore, following intravenous (IV) injection in rats, TP@Pd accumulated in lung tissue for 6 h, indicating the promising therapeutic efficacy via this administration route. Notably, the TP@Pd+NIR strategy demonstrated the excellent synergistic effects in alleviating lung inflammation storms, reducing diffuse alveolar damage, and accelerating lung tissue repair. Summarily, this work has designed a novel TP@Pd+NIR strategy for the synergistic enhancement of ALI amelioration, which may serve as a promising therapeutic approach for other ROS related diseases.

## Graphical abstract

The strategy of ultra‐small Pd loaded covalent organic framework combined with NIR irradiation (TP@Pd+NIR) is developed to reprogram lung redox homeostasis for the synergistic enhancement of acute lung injury immunotherapy via ROS scavenging, M2 directional polarization, NF‐κB pathway inhibition as well as tissue repair acceleration.

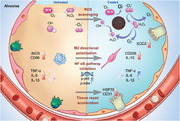

## 1 Introduction

Sepsis is a systemic inflammatory syndrome in response to infection, commonly observed in patients with severe trauma or infectious diseases, and often leading to multiple organ failure.^[^ ^1^ ^]^ Annually, the global incidence of sepsis exceeds 19 million cases, resulting in ≈6 million deaths and 3 million individuals with cognitive impairment.^[^ ^2^ ^]^ Notably, the lungs are among the most sensitive organs affected during the progression of sepsis, clinically manifesting as acute lung injury (ALI).^[^ ^3^ ^]^ ALI is a clinical syndrome characterized by chest distress and pain, severe hypoxemia, and respiratory failure.^[^ ^4^ ^]^ Despite advances in mechanical ventilation and symptomatic treatments, their efficacy remains uncertain due to high mortality rates and poor prognoses. Therefore, developing novel therapeutic strategies is of great significance for achieving safe and effective treatment of sepsis associated ALI.

Numerous studies have confirmed that ALI progression is always characterized by the excessive generated reactive oxygen species (ROS), leading to the oxidative injury in lungs. And the overproduction of ROS can activate NF‐κB pathway, subsequently upregulating the expression levels of pro‐inflammatory cytokines, which eventually results in pulmonary infiltration of massive immune cells, including macrophages, lymphocytes, and neutrophils.^[^ ^5^ ^]^ Conversely, inflammatory responses can alter pulmonary microenvironments, thereby leading to the overproduction of ROS and oxidative stress.^[^ ^6^ ^]^ Although the pathogenesis of ALI is influenced by multiple factors, excessive ROS generation, and inflammation storms are considered as the primary triggers of ALI.^[^ ^7^ ^]^ Therefore, developing a novel strategy that combines ROS scavenging and anti‐inflammation represents an efficient approach for ALI management.

Nanozymes, a class of nanomaterials with multiple enzymatic activities, are relatively stable, cost‐effective, and serve as the substitutes for natural enzymes and alternatives in nanobiomedicine, garnering significant interest in biomedical fields.^[^ ^8^ ^]^ Among the previously reported nanozymes, covalent organic frameworks (COFs), an emerging class of crystalline porous materials consisted of organic molecules connected by stable covalent bonds, have shown potentials in various biomedical applications, such as biosensing,^[^ ^9^ ^]^ anti‐tumor therapy,^[^ ^10^ ^]^ and anti‐inflammation therapy.^[^ ^11^ ^]^ This is attributed to their large specific surface area, regular pores, easily controllable structure, relatively high thermal and chemical stability, and multifunctionality.^[^ ^12^ ^]^ However, pure COFs exhibit limited catalytic activity and dispersion ability, which ultimately constrains their applications in catalytic therapy.^[^ ^13^ ^]^ Recent advancements in COF based nanomaterials primarily focus on forming nanocomposites by conjugating them with photosensitizers,^[^ ^14^ ^]^ ligands,^[^ ^15^ ^]^ drugs^[^ ^16^ ^]^ and other agents. Compared to other drug carriers such as mesoporous silicon and metal organic frameworks (MOFs), COFs offer superior biodegradability and they do not contain multivalent metals.^[^ ^17^ ^]^ Interestingly, beyond serving as drug carriers, COFs can also act as catalytic biomedicines,^[^ ^18^ ^]^ such as converting ·OH to H_2_O for ROS scavenging, or inducing ROS generation under external stimuli.^[^ ^19^ ^]^ To further enhance their catalytic activities, COFs have been designed to load various metal elements or metal oxides, including Au,^[^ ^20^ ^]^, Pt,^[^ ^21^ ^]^, Cu,^[^ ^22^ ^]^, and Mn.^[^ ^23^ ^]^ Notably, current metal loaded COFs still require stimulation via ultrasonic waves,^[^ ^24^ ^]^ near infrared (NIR) irradiation^[^ ^25^ ^]^ or ultraviolet (UV) stimuli to fully harness their comprehensive performance, thereby achieving optimal therapeutic effects for various diseases.

On the other hand, owing to its biocompatibility, NIR absorbance and ROS regulation ability, nanosized palladium (Pd) has been regarded as an efficient nanozyme for disease therapy.^[^ ^26^ ^]^ Notably, due to the quantum confinement of electrons in ultra‐small size regime,^[^ ^27^ ^]^ ultra‐small Pd exhibits exceptional catalytic activity for ROS scavenging. However, as the size of ultra‐small Pd decreases, its stability diminishes, making it prone to aggregation, and less likely to disperse uniformly.^[^ ^28^ ^]^ To address these issues, it is essential to select a carrier capable of uniformly and stably dispersing ultra‐small Pd, thereby enabling its comprehensive effects. In this context, an ideal COF based composited nanoplatform, with functions including enhanced anti‐inflammatory therapeutic effects, targeted delivery of ultra‐small metal with dispersion stability, and excellent photothermal therapy (PTT) efficacies, represents an urgent need. Nevertheless, such a novel treatment strategy has not yet been reported.

**Figure 1:**
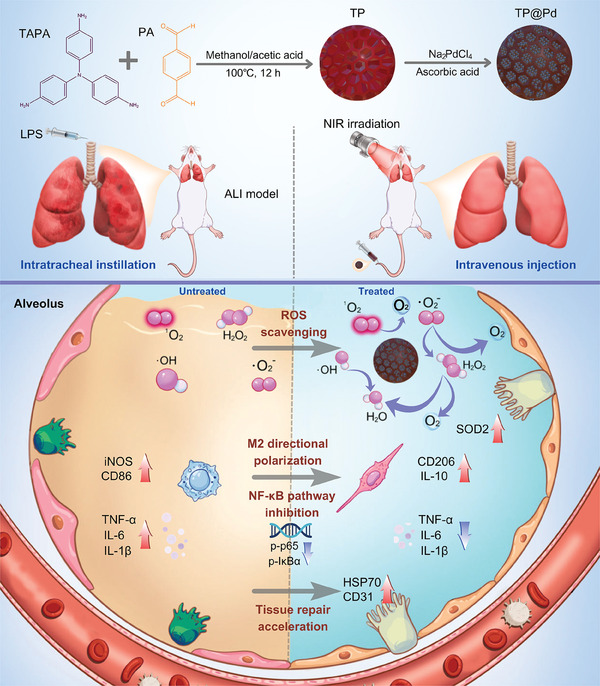
Schematic illustration of the fabrication of TP@Pd and synergistic enhanced ALI immunotherapy by the strategy of intravenous (IV) injection of TP@Pd combining with NIR irradiation via ROS scavenging, M2 directional polarization, NF‐κB pathway inhibition, and tissue repair acceleration.

In this study, we designed a novel composited nanoplatform (TP@Pd) by encapsulating tris(4‐aminophenyl)amine (TAPA) and p‐phthalaldehyde (PA) formed COF (TP) with ultra‐small Pd. Compared to TP and TP@Pd alone, TP@Pd+NIR demonstrated enhanced multiple enzyme catalytic activities and ROS scavenging capacities (**Figure** **1**). Additionally, as shown in lipopolysaccharide (LPS) induced macrophages and ALI rat models, TP@Pd+NIR efficiently reduced intracellular ROS and inflammatory cytokine levels while promoting antioxidant and tissue repair factor levels, all with favorable biosafety. Mechanistically, TP@Pd+NIR induced macrophage M2 polarization, regulated CD4^+^/CD8^+^ T cells numbers, and suppressed NF‐κB signaling pathway, thereby reprogramming lung redox homeostasis to ameliorate ALI. Consequently, the synergistic effects of ROS scavenging and photothermal therapy offered an effective strategy for reprogramming organ redox homeostasis, ultimately facilitating the immunotherapy of ROS related diseases.

## 2 Results and Discussion

### 2.1 Preparation and Physicochemical Characterization

**Figure 2:**
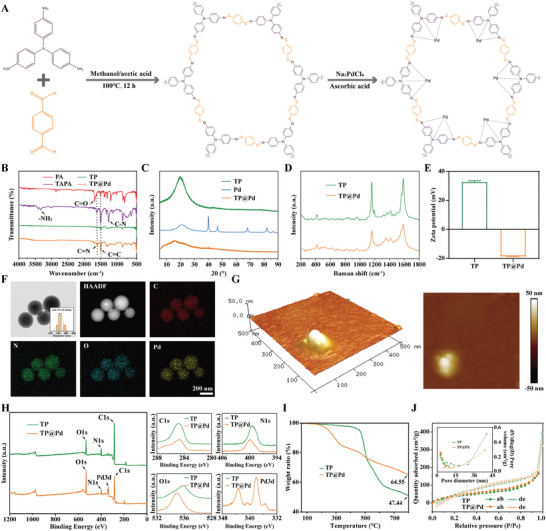
Preparation and basic characterization of TP@Pd. A) Synthesis procedures of TP@Pd. B) FTIR results of TAPA, PA, TP, and TP@Pd. C) XRD results of TP, Pd, and TP@Pd. D) Raman results of TP and TP@Pd. E) Zeta potential of TP and TP@Pd. F) TEM‐mapping images of TP@Pd and the corresponding element composition (HAADF, C, N, O, and Pd images). G) AFM image of TP@Pd. H) XPS results of TP and TP@Pd. I) TGA results of TP and TP@Pd. J) BET results of TP and TP@Pd.

COFs not only function as efficient drug carriers but also act as nanozymes with multiple catalytic activities, enabling their application in biomedical fields. Herein, the novel TP (red) was prepared via a hydrothermal reaction of TAPA (gray) and PA (beige) in a mixture of methanol and acetic acid (Figure S1, Supporting Information). The stable TP was formed through a Schiff base reaction between the amino groups of TAPA and aldehyde groups of PA. Subsequently, the composited nanoplatform TP@Pd (brown) was fabricated by loading TP with ultra‐small Pd (Figure S1, Supporting Information). The molecular structures of the chemicals were characterized by Ultraviolet‐visible spectroscopy (UV‐vis) and Fourier transform infrared  spectrometer (FTIR). As shown in Figure S2 (Supporting Information), an obvious peak was emerged at 300 nm, corresponding to the double bonds of benzene ring in TAPA, while another peak existed at 262 nm, another double bonds of benzene ring in PA. After forming TP and TP@Pd, no peaks were observed, indicating their successful formation, and the absence of unreacted TAPA and PA. Furthermore, the ultra‐small Pd loading did not introduce additional peaks for TP@Pd. As illustrated in **Figure** **2**B, prominent peaks were observed at 3336.85 cm⁻¹, 1616.35 cm⁻¹, 1496.76 cm⁻¹ and 1255.66 cm⁻¹, respectively corresponding to NH₂, C═N, C═C and C─N groups of TAPA. Additionally, C═O and C═C peaks were detected for PA at 1683.86 cm⁻¹ and 1498.69 cm⁻¹, respectively. After forming TP, the NH₂ peak disappeared, while the peaks for C═N, C═C and C─N groups remained. The disappearance of NH₂ peak and the persistence of C═N group indicated a reaction between the NH₂ group of TAPA and the C═O group of PA, leading to the formation of TP. There were no significant differences in the FTIR spectra of TP with or without Pd loading. The crystallinity of Pd, TP, and TP@Pd was further analyzed by using X‐ray diffraction (XRD). As illustrated in Figure 2C, while nanosized Pd exhibited several peaks, TP displayed only a single peak, confirming its monomorphism. However, TP@Pd showed no additional peaks, and only exhibited a broader peak compared to TP. Similarly, Raman spectroscopy revealed no significant differences between TP and TP@Pd, indicating that the molecular structure of TP was preserved after Pd loading (Figure 2D). The zeta potential analysis showed a value of 32.63 ± 1.03 mV for TP, which shifted to −18.50 ± 0.46 mV for TP@Pd after Pd loading (Figure 2E). In TP@Pd, ultra‐small Pd occupied the pores of TP, becoming trapped within its framework without altering its molecular structure. However, the homogeneous distribution of ultra‐small Pd in the TP pores affected the surface charges and their distribution, as reflected in the zeta potential differences.^[^ ^29^ ^]^

The morphology of TP and TP@Pd was investigated using transmission electron microscopy (TEM)‐mapping. As observed in Figure 2F and Figure S3 (Supporting Information), TP exhibited a spherical shape with a diameter of 216.15 ± 17.16 nm, and it retained its spherical morphology with a slightly increased diameter of 231.97 ± 31.69 nm after Pd loading for TP@Pd (Table S1, Supporting Information). From the mapping results, only C, N and O elements were displayed in TP (Figure S3, Supporting Information), whereas TP@Pd displayed the presence of C, N, O and Pd elements (Figure 2F). TP@Pd was further characterized by atomic force microscope (AFM). As shown in Figure 2G, TP@Pd exhibited a height of ≈100 nm, and a diameter of about 200 nm, confirming its 3D spherical shape. Additionally, X‐ray photoelectron spectroscopy (XPS) was employed to analyze the elemental composition of TP and TP@Pd. As shown in Figure 2H, C, N and O elements were prominently observed in TP, while TP@Pd displayed an additional Pd element. The elemental composition ratios for TP were 80.42%, 7.86% and 11.72% for C, N and O, respectively. For TP@Pd, the ratios were 68.36%, 6.91%, 22.87% and 1.86% for C, N, O and Pd, respectively (Table S2, Supporting Information). Furthermore, inductively coupled plasma optical emission spectrometer (ICP‐OES) analysis revealed that TP@Pd contained 8.60 ± 0.21% Pd (Table S2, Supporting Information).

The weight loss of TP and TP@Pd was analyzed using thermogravimetric analysis (TGA). As illustrated in Figure 2I, TP exhibited a weight loss ratio of 52.56%, which decreased to 35.45% for TP@Pd. This difference was attributed to the Pd loading ratio and the residual structure of TP. Additionally, Brunauer‐Emmett‐Teller (BET) analysis revealed that the adsorption and desorption curves were nearly identical for TP and TP@Pd (Figure 2J). The pore size, pore volume and specific surface area were tested as 16.85 nm, 0.63 cm^3^ g^−1^ and 161.32 m^2^ g^−1^ for TP, and 12.14 nm, 0.51 cm^3^ g^−1^ and 192.30 m^2^ g^−1^ for TP@Pd, respectively (Table S3, Supporting Information). These changes in specific surface area and porosity were primarily attributed to Pd loading on TP@Pd.^[^ ^30^ ^]^ The in vitro degradation of TP@Pd was also evaluated. As shown in Figure S4 (Supporting Information), TP@Pd initially exhibited a spherical shape, retaining a similar structure over time in phosphate buffered saline (PBS). However, at pH 5.0, TP@Pd exhibited slight swelling after 1 h, and was completely degraded after 4 h. The size and its polydispersity index (PDI), and zeta potential of TP@Pd were analyzed using the zeta sizer. As shown in Figure S5 (Supporting Information), TP@Pd had an initial size of 444.03 ± 17.05 nm, and a PDI of 0.17 ± 0.00, which remained nearly unchanged over time in PBS, indicating its strong stability. The size differences observed between zeta sizer and TEM were attributed to the aggregation of TP@Pd, as zeta sizer measurements could not reflect the true size of TP@Pd. The zeta potential of TP@Pd also remained stable over time in PBS (Figure S6, Supporting Information). However, at pH 5.0, the zeta potential of TP@Pd initially was −18.50 mV, and progressively decreased over time, reaching −51.82 mV, −55.38 mV, −61.07 mV, −62.50 mV, −67.05 mV and −64.63 mV at 0.5, 1, 2, 4, 8, and 24 h, respectively (Figure S7, Supporting Information). The dynamic balance of ─C═N─ groups in TP contributed to the good degradability of TP@Pd under weak acidic conditions, which ultimately led to the destruction of hollow COF structure.

From the above findings, the successful formation of TP and TP@Pd was confirmed. TP was synthesized through a Schiff base reaction between amino groups and aldehyde groups, with the relatively stable amide groups contributing to the stability of COF structure. Additionally, ultra‐small Pd was incorporated into TP to form TP@Pd via in‐situ reduction using ascorbic acid. The incorporation of Pd could not significantly alter the molecular structure, chemical composition, crystallinity, morphology, or diameter of TP. However, it did affect the zeta potential, elemental composition, pore size, pore volume, surface energy and weight loss ratio.

### 2.2 Dispersion, Photothermal Effects, and ROS Scavenging Abilities Investigation

**Figure 3:**
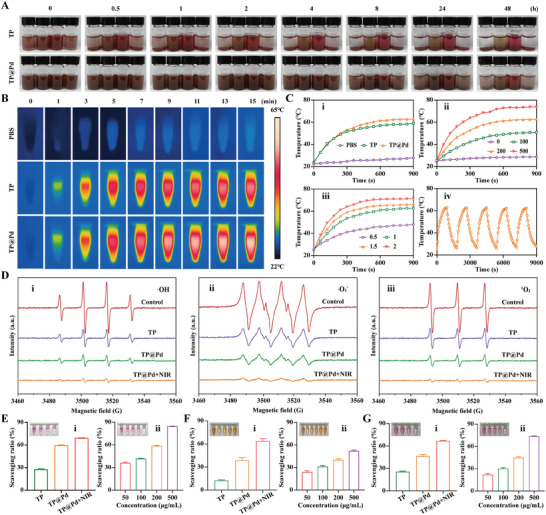
Physicochemical properties of NPs. A) Dispersion and stability of TP and TP@Pd at predetermined time points (0, 0.5, 1, 2, 4, 8, 24, and 48 h), and the corresponding solutions were PBS, FBS, DMEM, and H_2_O_2_ (5 mM) respectively (from left to right). B) Photothermal images of PBS, TP, and TP@Pd with the same concentration of 200 µg mL^−1^ under NIR irradiation (1 W cm^−2^) versus time. C) Temperature changes of PBS, TP, and TP@Pd with the same concentration of 200 µg mL^−1^ under NIR irradiation (1 W cm^−2^) (i), different concentration (0, 100, 200, and 500 µg mL^−1^) of TP@Pd under NIR irradiation (1 W cm^−2^) (ii), and 200 µg mL^−1^ TP@Pd under different power intensity of NIR irradiation (0.5, 1, 1.5 and 2 W cm^−2^) (iii) versus time, and photothermal stability of 200 µg mL^−1^ TP@Pd under NIR irradiation (1 W cm^−2^) for 5 “on” and “off” cycles (iv). D) ROS scavenging ability of different NPs by ESR: ·OH (i), ·O_2_^−^ (ii) and ^1^O_2_ (iii). E) ROS scavenging capacity by CAT testing kit: different NPs with 200 µg mL^−1^ (i) and TP@Pd with different concentrations (ii). F) ROS scavenging capacity by ·OH testing kit: different NPs with 200 µg mL^−1^ (i) and TP@Pd with different concentrations (ii). G) ROS scavenging capacity by ·O_2_^−^ testing kit: different NPs with 200 µg mL^−1^ (i) and TP@Pd with different concentrations (ii).

For in vivo therapy, nanoparticles (NPs) must maintain stable dispersion, and resist aggregation to complete blood circulation and accumulate in target organs. Dispersion testing was conducted by dispersing TP and TP@Pd in different solutions: PBS to reflect normal physiological conditions, Dulbecco's modified eagle medium (DMEM) and fetal bovine serum (FBS) to simulate cell culture conditions, and 5 mM H₂O₂ to represent ROS conditions.^[^ ^31^ ^]^ TP alone was homogeneously dispersed in PBS, FBS, DMEM and 5 mM H₂O₂ at the start. By 0.5 h, deposition began in PBS and 5 mM H₂O₂, while TP remained stable in DMEM and FBS. After 1 h, TP completely settled at the bottom of PBS and 5 mM H₂O₂, leaving a transparent supernatant, and fully precipitated in DMEM and FBS by 8 h. Conversely, TP@Pd remained completely dispersed in all solutions up to 4 h. It began to deposit at 8 h, and was almost fully precipitated in PBS and 5 mM H₂O₂ by 48 h. Notably, TP@Pd maintained partial dispersion in DMEM and FBS even at 48 h (**Figure** **3**A). Besides, the dispersion of TP and TP@Pd in blood serum was also investigated. As displayed in Figure S8 (Supporting Information), both TP and TP@Pd were homogeneously distributed in blood serum at the start. For TP, gradual deposition occurred over time, with complete sedimentation by 8 h. In contrast, TP@Pd remained largely dispersed at 8 h, with only a small fraction deposited. By 24 h, most TP@Pd had settled, although some dispersion persisted. These results confirmed that TP@Pd exhibited significantly better dispersion and stability in various solutions compared to TP.^[^ ^32^ ^]^ The enhanced dispersion and stability of TP@Pd could be attributed to TP's role as a carrier for ultra‐small Pd, which contributed to the dispersion of ultra‐small Pd.^[^ ^33^ ^]^ Additionally, Pd loading efficiently reduced the surface energy of TP@Pd, preventing aggregation in aqueous solutions, and further improving its stability and dispersion.^[^ ^34^ ^]^

PTT has been regarded as an efficient non‐invasive therapeutic strategy for various diseases due to its advantages of short treatment duration, excellent therapeutic efficacy, and minimal toxic side effects. As shown in Figure 3B, under NIR irradiation, the temperatures of TP and TP@Pd significantly increased, while no noticeable temperature changes were observed for PBS over time. At the concentration of 200 µg mL^−1^, the temperature increased to 59.0 °C and 62.7 °C for TP and TP@Pd after 15 min of NIR irradiation (808 nm, 1 W cm^−2^) (i in Figure 3C). When the concentration of TP@Pd was increased from 0 to 100, 200 and 500 µg mL^−1^, the corresponding temperatures rose from 28.8 °C to 50.9 °C, 62.6 °C and 73.8 °C, respectively (ii in Figure 3C). Similarly, when the irradiation intensity of NIR light increased from 0.5 to 1, 1.5 and 2 W cm^−2^, the temperatures increased from 48.2 °C to 62.8 °C, 66.4 °C and 71.4 °C, respectively (iii in Figure 3C). Notably, even after five “on” and “off” cycles, the photothermal effects remained stable, with a consistent trend in temperature changes observed (iv in Figure 3C). The vibration, rotation and electronic excitation induced by NIR irradiation caused strong collisions and friction among NPs, thereby generating heat. Increasing the concentration of NPs and the power intensity of NIR light enhanced the photothermal effects. Additionally, the stable COF structure of TP@Pd contributed to its consistent photothermal performance.

The ROS scavenging abilities of NPs were evaluated using electron spin resonance (ESR) and ROS testing kits. ESR was employed to characterize unpaired electrons, such as those in ROS. As displayed in Figure 3D, compared to control group, NPs obviously reduced the curves of ·OH, ·O₂^−^, and ¹O₂, with the ROS scavenging ability ranking as TP < TP@Pd < TP@Pd+NIR. Additionally, ROS scavenging abilities were quantified using ROS testing kits. For catalase (CAT) activity testing, the H₂O₂ scavenging ratio was 27.74 ± 0.75%, 59.75 ± 0.48% and 69.30 ± 0.26% for TP, TP@Pd and TP@Pd+NIR, respectively, at the concentration of 200 µg mL^−1^ (i in Figure 3E; Table S4, Supporting Information). For TP@Pd, the scavenging ratio significantly increased from 36.32 ± 0.84% to 84.69 ± 0.20% as the concentration rose from 50 to 500 µg mL^−1^ (ii in Figure 3E; Table S5, Supporting Information). Similarly, for ·OH scavenging, the ratio was 12.45 ± 0.93% for TP, increasing to 38.73 ± 3.75% for TP@Pd, and 64.00 ± 2.93% for TP@Pd+NIR at the concentration of 200 µg mL^−1^ (i in Figure 3F; Table S5, Supporting Information). As the concentrations of TP@Pd increased from 50 to 500 µg mL^−1^, the corresponding ·OH scavenging ratio rose from 24.13 ± 1.45% to 52.26 ± 0.98% (ii in Figure 3F; Table S5, Supporting Information). For ·O₂^−^ scavenging, TP@Pd+NIR demonstrated the highest scavenging ratio of 66.90 ± 0.51%, followed by TP@Pd (46.62 ± 1.86%) and TP (25.69 ± 0.77%) at 200 µg mL^−1^ (i in Figure 3G; Table S4, Supporting Information). A high concentration of TP@Pd (500 µg mL^−1^) resulted in an enhanced ·O₂⁻ scavenging ratio of 73.79 ± 0.63% (ii in Figure 3G; Table S5, Supporting Information). The ROS scavenging capacities of individual components, including nanosized Pd, TP, and NIR irradiation alone, were also tested. As shown in Figure S9A (Supporting Information), the H₂O₂ scavenging ratio of Pd was 78.07 ± 0.14%, which decreased to 28.68 ± 0.32% and 1.73 ± 0.71% for TP and NIR irradiation, respectively. The ·OH and ·O₂⁻ scavenging ratios were 32.51 ± 1.26% and 44.24 ± 1.06% for Pd, 12.62 ± 0.95% and 23.31 ± 1.45% for TP, and 3.43 ± 2.18% and 6.19 ± 1.92% for NIR irradiation alone (Figure S9B,C and Table S6, Supporting Information). These results indicated that TP exhibited a certain level of ROS scavenging capacity due to the abundance of amino groups, while nanosized Pd displayed excellent ROS scavenging capacities.^[^ ^35^ ^]^ NIR irradiation alone contributed minimally to ROS scavenging.^[^ ^36^ ^]^ However, the incorporation of ultra‐small Pd enhanced the free energy, and introduced external defects in TP@Pd, leading to improved ROS scavenging abilities. Notably, under NIR irradiation, the movement of ultra‐small Pd was intensified, which improved adsorption energy, and further enhanced the scavenging capacities of various ROS for TP@Pd+NIR.

### 2.3 Biological Activities Investigation in Cellular Levels

**Figure 4:**
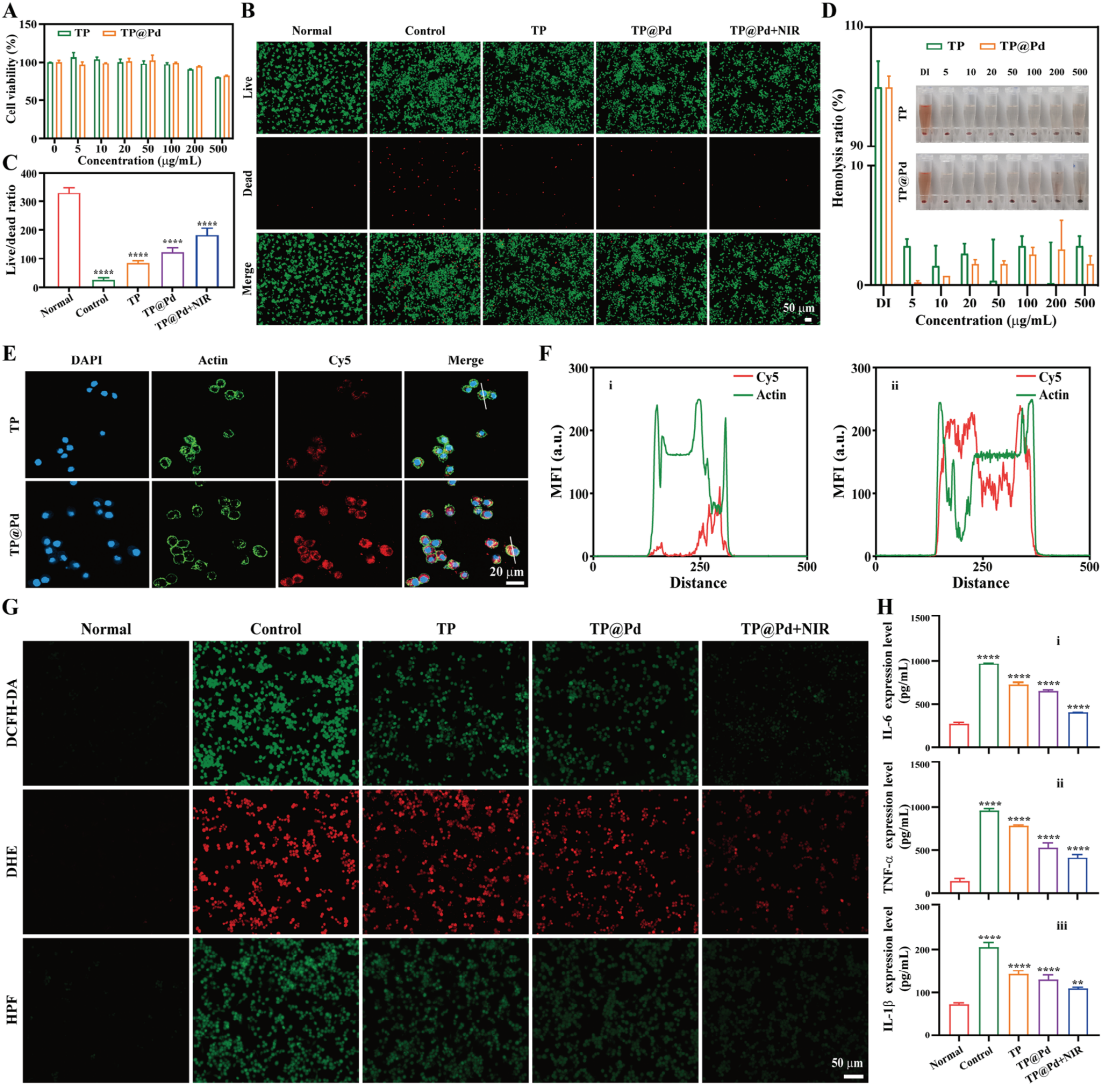
A) Cell viability of TP and TP@Pd with different concentrations ranging from 0 to 500 µg mL^−1^. B) Live/dead staining images of treated cells and their corresponding quantified results (C). D) Blood biocompatibility of TP and TP@Pd with different concentrations ranging from 5 to 500 µg mL^−1^. E) Cellular uptake images of cells incubated with Cy5‐TP and Cy5‐TP@Pd for 3 h by confocal microscope and the corresponding co‐localization results: Cy5‐TP (i) and Cy5‐TP@Pd (ii) (F). G) Intracellular ROS (DCFH‐DA, DHE, and HPF) levels of treated cells by fluorescent microscopy. H) Inflammatory factors (IL‐6 (i), TNF‐α (ii) and IL‐1β (iii)) expression levels of the supernatant of treated cells by ELISA. The corresponding groups were: cells without treatment (normal group), cells pre‐treated with LPS followed by incubating with PBS (control group), cells pre‐treated with LPS followed by incubating with 200 µg mL^−1^ TP (TP), cells pre‐treated with LPS followed by incubating with 200 µg mL^−1^ TP@Pd (TP@Pd), and cells pre‐treated with LPS followed by incubating with 200 µg mL^−1^ TP@Pd and NIR irradiation (1 W cm^−2^) (TP@Pd+NIR). (“*” symbol compared with normal group, *p < 0.05, **p < 0.01, ***p < 0.001 and ****p < 0.0001).

Macrophages, as vital components of the body's immune system, play the significant role in various diseases, and also in different stages of the same disease. During these stages, multiple signaling pathway molecules regulate and activate macrophage polarization, resulting in the secretion of pro‐inflammatory and anti‐inflammatory mediators, promotion of tissue repair, and regulation of immune responses throughout the occurrence, progression and resolution of inflammatory diseases.^[^ ^37^ ^]^ In this study, murine macrophage cells (RAW264.7) was selected as the target cells for investigation. And the cell viability of RAW264.7 was tested by using the cell counting kit‐8 (CCK‐8) assay. As shown in **Figure** **4**A, cell viability exceeded 90% for both TP and TP@Pd at the concentrations ranging from 0 to 200 µg mL^−1^, but began to decrease at the concentrations above 200 µg mL^−1^. Therefore, 200 µg mL^−1^ was selected as the concentration of NPs for subsequent experiments. Next, the oxidative stress protection ability of NPs was also investigated through live/dead staining of treated cells. As illustrated in Figure 4B, normal group displayed a large number of live cells (green intensity), whereas LPS treated cells (control group) exhibited a substantial increase in dead cells (red intensity). Incubation with NPs effectively reduced the number of dead cells, with TP@Pd+NIR showing the most pronounced effects, followed by TP@Pd and TP. Statistical analysis of the live/dead ratio revealed the values of 330.83 ± 18.78, 25.07 ± 7.71, 84.03 ± 8.51, 122.72 ± 15.79 and 182.75 ± 23.66 for normal group, control group, TP, TP@Pd and TP@Pd+NIR, respectively (Figure 4C).

Furthermore, NPs must be injected into the blood stream, and directly interact with blood. Therefore, a hemolysis testing was conducted to assess the blood compatibility of NPs. As shown in Figure 4D, in contrast to deionized (DI) water, which exhibited significant hemolysis, no hemolysis was observed for either TP or TP@Pd across the tested concentration ranges. The hemolysis ratio for both NPs remained below 5%,^[^ ^38^ ^]^ demonstrating the excellent hemocompatibility of TP and TP@Pd.

To maximize their functionality, it is essential for NPs to be effectively taken up by target cells. Thus, the cellular uptake abilities of TP and TP@Pd were evaluated. Cy5‐PEG2000‐Thiol, containing a thiol group, was reacted with the double bonds of TP and TP@Pd to form stable Cy5 labeled TP and TP@Pd (Cy5‐TP and Cy5‐TP@Pd). The Cy5 labeled NPs were then incubated with cells for 3 h before observation. As observed in Figure 4E, after 3 hours’ incubation, minimal fluorescence was observed for TP, whereas TP@Pd exhibited significant fluorescence, indicating that the cellular uptake ability of TP@Pd was superior to that of TP. Similarly, the degree of overlap between red fluorescence (Cy5) and green fluorescence was substantially higher for Cy5‐TP@Pd compared to Cy5‐TP (Figure 4F). Statistical analysis revealed that the mean fluorescent intensity (MFI) was 40.85 ± 2.06 for TP and 75.91 ± 0.55 for TP@Pd (Figure S10, Supporting Information). The enhanced cellular uptake of TP@Pd by macrophages through endocytosis likely contributed to improved biological functions at the cellular level.^[^ ^39^ ^]^ Additionally, the co‐localization of TP@Pd and lysosomes in macrophages was investigated. As shown in Figure S11A (Supporting Information), green fluorescence (Lyso‐Tracker) and red fluorescence (Cy5‐TP@Pd) were observed around the nucleus (4', 6‐diamidino‐2‐phenylindole dilactate (DAPI)), though they were not completely overlapping. Further analysis revealed partial overlap between TP@Pd and lysosomes, while a majority of TP@Pd was not overlapping, suggesting that TP@Pd could escape from lysosomes (Figure S11B, Supporting Information) and perform its intracellular functions.^[^ ^40^ ^]^

Intracellular ROS levels are indicative of the ROS scavenging abilities of NPs within cells. These levels were evaluated using ROS‐specific probes, including 2', 7'‐dichlorodihydrofluorescein diacetate (DCFH‐DA, Maokangbio, China), dihydroethidium (DHE, Maokangbio, China) or hydroxyphenyl fluorescein (HPF, Maokangbio, China). As imaged in Figure 4G, comparing with normal cells (normal group) with minimal fluorescence, the intracellular ROS levels in LPS stimulated cells were significantly elevated, as indicated by the strong fluorescence observed with the DCFH‐DA, DHE, and HPF probes. TP and TP@Pd effectively reduced the intracellular ROS levels, as evidenced by the decline in fluorescence intensity. Notably, TP@Pd+NIR exhibited the most pronounced reduction in fluorescence, highlighting its superior ability to lower intracellular ROS levels. Statistical analysis revealed that, for the DCFH‐DA probe, the MFI was 10.18 ± 0.34 for normal group, 78.44 ± 2.89 for control group, 49.21 ± 1.82 for TP, 31.67 ± 2.59 for TP@Pd, and 20.07 ± 0.96 for TP@Pd+NIR (i in Figure S12, Supporting Information). A similar trend was observed in intracellular ·O₂⁻ (DHE, ii in Figure S12, Supporting Information) and ·OH (HPF, iii in Figure S12, Supporting Information) levels, with the order of ROS levels: control group >TP >TP@Pd >TP@Pd+NIR >normal group. In addition, intracellular ROS levels were quantified analyzed by using flow cytometry (CytoFLEX LX, China). As shown in Figure S13A (Supporting Information), the MFI of DCFH‐DA was 353 for normal group and significantly increased to 2745 for control group. TP, TP@Pd, and TP@Pd+NIR reduced the MFI, with TP@Pd+NIR showing the greatest reduction. Similarly, the MFI values of DHE and HPF were 352 and 526, respectively, for normal group, increasing to 5645 and 2659 for control group. These values were reduced to 3645 and 1893 for TP, 1788 and 1716 for TP@Pd, and 626 and 1192 for TP@Pd+NIR, respectively (Figure S13B,C, Supporting Information). These results confirm that TP, TP@Pd, and TP@Pd+NIR effectively reduce intracellular ROS levels, with TP@Pd+NIR demonstrating the highest efficiency. Extracellular ROS levels were also evaluated by testing the ROS levels of the supernatant of treated cells. As illustrated in Figure S14 (Supporting Information), the fluorescent intensities of DCFH‐DA, DHE, and HPF were 880.00 ± 20.88, 1545.67 ± 40.70, and 1712.00 ± 126.93, respectively, for normal group, which significantly increased to 1292.67 ± 51.64, 2545.33 ± 199.36, and 2424.67 ± 89.72 for control group, indicating elevated extracellular ROS levels due to LPS induction. TP, TP@Pd, and TP@Pd+NIR reduced the fluorescent intensities, reflecting a decrease in extracellular ROS levels. Among them, TP@Pd+NIR exhibited the most effective reduction of extracellular ROS levels.

Furthermore, the antioxidant and anti‐inflammatory capacities of NPs were initially investigated using enzyme‐linked immunosorbent assay (ELISA). As shown in Figure 4H, the IL‐6, TNF‐α, and IL‐1β expression levels were 968.03 ± 7.51, 953.89 ± 29.54, and 204.62 ± 11.22, respectively, in control group. These levels significantly increased in LPS induced cells. TP slightly reduced the expression levels of these inflammatory factors to 724.74 ± 25.56 for IL‐6, 778.33 ± 14.53 for TNF‐α, and 141.77 ± 8.11 for IL‐1β. TP@Pd further enhanced this effect, lowering the levels to 652.25 ± 13.90 for IL‐6, 527.22 ± 53.29 for TNF‐α, and 129.03 ± 10.94 for IL‐1β. Notably, TP@Pd+NIR exhibited the most potent anti‐inflammatory activity, reducing the levels of IL‐6, TNF‐α, and IL‐1β expression to 403.09 ± 5.50, 411.39 ± 39.46, and 107.47 ± 3.63 respectively. Additionally, compared to control group (101.61 ± 15.99), the TNF‐α expression level was obviously reduced by Pd (33.49 ± 2.20) and slightly decreased by TP (74.35 ± 0.68). However, NIR irradiation alone had minimal impact, with TNF‐α levels remaining at 83.28 ± 10.57. Conversely, IL‐10 expression, which was lowest in control group (20.86 ± 1.64), showed minimal change for NIR irradiation (22.19 ± 3.85), slight elevation for TP (31.90 ± 3.75), and significant enhancement for Pd (85.62 ± 5.29) (Figure S15, Supporting Information). These findings confirm that Pd more efficiently downregulated TNF‐α expression levels and upregulated IL‐10 expression levels compared to TP. NIR irradiation alone had minimal or negligible effects on TNF‐α and IL‐10 expression.

**Figure 5:**
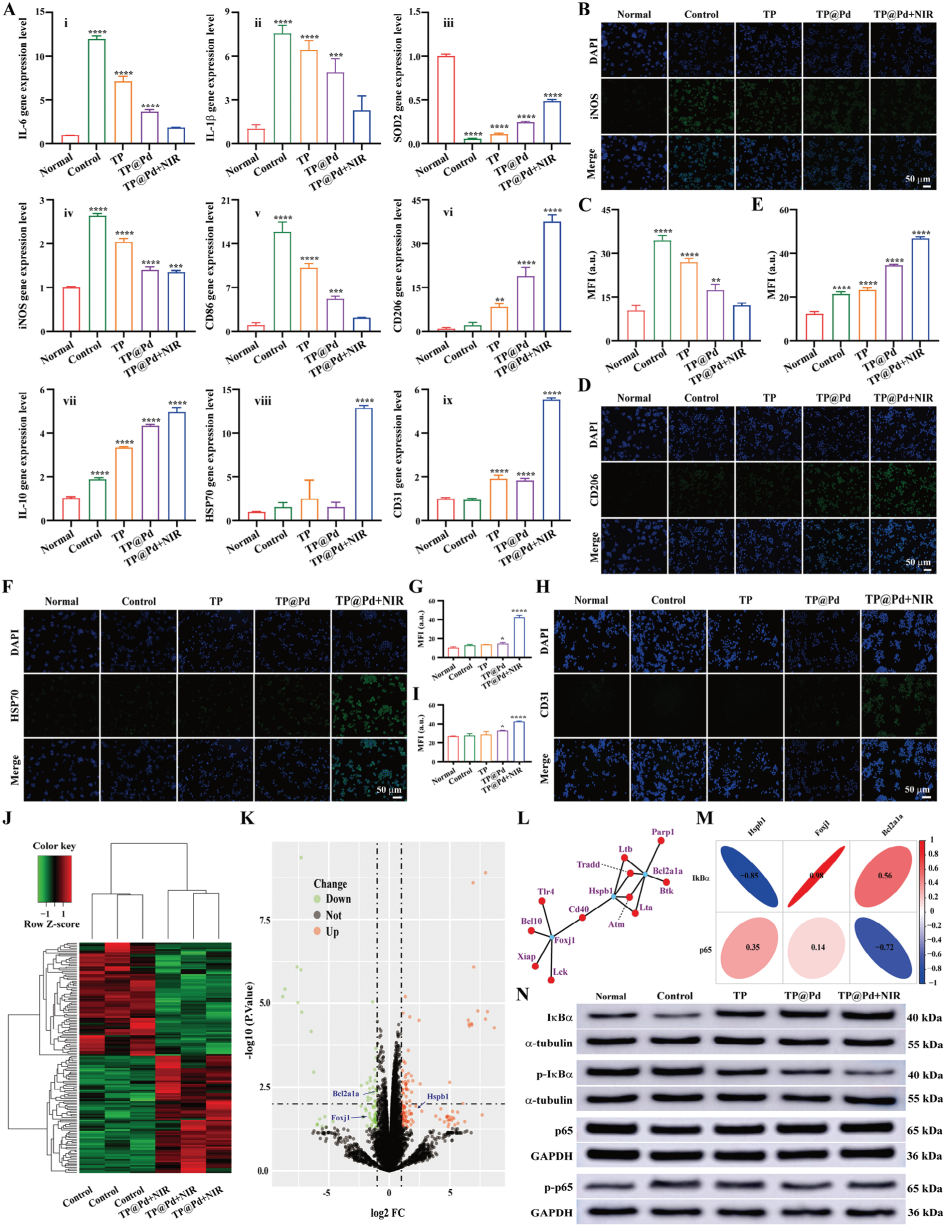
A) Relative genes expression levels of treated cells by RT‐qPCR: IL‐6 (i), IL‐1β (ii), SOD2 (iii), iNOS (iv), CD86 (v), CD206 (vi), IL‐10 (vii), HSP70 (viii), and CD31 (ix). B) iNOS expression level of treated cells by fluorescent microscope and the corresponding quantified results (C). D) CD206 expression level of treated cells by fluorescent microscope and the corresponding quantified results (E). F) HSP70 expression level of treated cells by fluorescent microscope and the corresponding quantified results (G). H) CD31 expression level of treated cells by fluorescent microscope and the corresponding quantified results (I). J) A heatmap of DEGs between control group and TP@Pd+NIR. K) A volcano plotting of the landscape of DEGs. L) A gene interaction network of the relationship between NF‐κB pathway related genes (Red node) and three genes of interest (Blue node). M) A correlation heatmap of the correlation between IκBα/p65 and three genes of interest. N) Relative proteins expression levels of treated cells by WB. The corresponding groups were: cells without treatment (normal group), cells pre‐treated with LPS followed by incubating with PBS (control group), cells pre‐treated with LPS followed by incubating with 200 µg mL^−1^ TP (TP), cells pre‐treated with LPS followed by incubating with 200 µg mL^−1^ TP@Pd (TP@Pd), and cells pre‐treated with LPS followed by incubating with 200 µg mL^−1^ TP@Pd and NIR irradiation (1 W cm^−2^) (TP@Pd+NIR). (“*” symbol compared with normal group, *p < 0.05, **p < 0.01, ***p < 0.001, and ****p < 0.0001).

In addition, reverse transcription‐quantitative real‐time polymerase chain reaction (RT‐qPCR) was performed to quantify the expression levels of inflammatory genes (IL‐6 and IL‐1β), antioxidant genes (SOD2), M1 type genes (iNOS and CD86), M2 type genes (CD206 and IL‐10), and tissue repair genes (HSP70 and CD31), reflecting the antioxidant, anti‐inflammatory, and tissue repair capacities of NPs (**Figure** **5**A). Consistent with the enzyme linked immunosorbent assay (ELISA) results, LPS stimulation significantly increased the expression levels of inflammatory genes (IL‐6: 12.02 ± 0.34, IL‐1β: 7.54 ± 0.56) and M1 type genes (iNOS: 2.64 ± 0.05, CD86: 15.95 ± 1.60), decreased the antioxidant gene expression (SOD2: 0.05 ± 0.00), and affected the expression levels of M2 type genes (CD206: 2.15 ± 0.90, IL‐10: 1.87 ± 0.07) in control group compared to normal group. However, NPs treatment efficiently reduced the expression levels of inflammatory (IL‐6 and IL‐1β) and M1 type genes (iNOS and CD86), while enhancing the expression levels of antioxidant (SOD2) and M2 type genes (CD206 and IL‐10) in LPS stimulated cells. Among the treatments, TP@Pd+NIR demonstrated the most effective regulation, followed by TP@Pd and TP. Specifically, for IL‐10, a cytokine involved in inflammation and immune suppression, LPS stimulation induced a moderate upregulation as part of the inflammatory response.^[^ ^41^ ^]^ For individual components of TP@Pd+NIR, Pd was the most efficient in downregulating the expression levels of IL‐6 (98.67 ± 7.37), IL‐1β (3.95 ± 0.48), and iNOS (2.28 ± 0.22), compared to control group (254.40 ± 31.22, 7.63 ± 0.31, and 3.92 ± 0.23, respectively), followed by TP (155.05 ± 9.57, 5.77 ± 0.85, and 2.85 ± 0.33) (Figure S16A–C, Supporting Information). Conversely, the expression level of SOD2, which was lowest in control group and NIR‐treated cells, was only slightly reduced for Pd (0.81 ± 0.07) compared to normal group (1.00 ± 0.04) (Figure S16D, Supporting Information). Notably, NIR irradiation alone had minimal effects on the expression levels of IL‐6 (221.14 ± 11.93), IL‐1β (7.98 ± 0.65), iNOS (3.45 ± 0.07), and SOD2 (0.10 ± 0.01), which remained the similar to those in control group (Figure S16, Supporting Information). Furthermore, the expression levels of tissue repair genes (HSP70 and CD31) were analyzed. As shown in viii and ix in Figure 5A, HSP70 (12.86 ± 0.28) and CD31 (5.55 ± 0.07) levels were highest for TP@Pd+NIR, followed by TP (2.51 ± 2.12 and 1.92 ± 0.16), TP@Pd (1.56 ± 0.52 and 1.84 ± 0.09), control group (1.56 ± 0.50 and 0.95 ± 0.06), and normal group (1.00 ± 0.06 and 1.00 ± 0.05) respectively.

Finally, immunofluorescent staining was conducted to analyze the expression levels of related factors in treated cells. After LPS stimulation, IL‐6 and iNOS expression levels were significantly elevated, as indicated by high fluorescence intensity compared to normal group. Conversely, CD206 expression was slightly ascended in control group, with a little fluorescence observed. Incubation with NPs effectively decreased IL‐6 and iNOS expression levels, as demonstrated by reduced fluorescence intensity, and increased CD206 expression levels, as evidenced by enhanced fluorescence. Among the treatments, TP@Pd+NIR exhibited the most pronounced effects, followed by TP@Pd and TP (Figure S17, Supporting Information; Figure 5B,D, Supporting Information). Statistical analysis showed that the MFI of IL‐6 expression was 8.05 ± 0.64, 38.75 ± 1.35, 28.75 ± 1.25, 18.21 ± 0.56, and 14.19 ± 0.15 for normal group, control group, TP, TP@Pd, and TP@Pd+NIR, respectively (Figure S18, Supporting Information). Similarly, the MFI of iNOS expression decreased from 34.38 ± 1.57 in control group to 26.81 ± 1.27 for TP, 17.28 ± 2.01 for TP@Pd, 12.11 ± 0.72 for TP@Pd+NIR, and 10.38 ± 1.66 for normal group (Figure 5C). Conversely, the order of the MFI of CD206 expression was TP@Pd+NIR (47.14 ± 0.66) >TP@Pd (34.73 ± 0.41) >TP (23.45 ± 0.91) >control group (21.60 ± 1.00) >normal group (12.58 ± 0.95) (Figure 5E). These findings confirm that TP@Pd+NIR achieved the lowest MFI for IL‐6 and iNOS expression, and the highest MFI for CD206 expression, indicating its superior efficiency in suppressing inflammatory factor expression and inducing M2 polarization of macrophages. Additionally, the expression levels of tissue repair genes, HSP70 and CD31, were evaluated using immunofluorescent staining. As shown in Figure 5F,H, TP@Pd+NIR demonstrated significantly higher expression levels of HSP70 and CD31 compared to other groups. From the statistical analysis, it revealed that the MFI of HSP70 was 10.43 ± 1.09, 13.01 ± 1.03, 13.85 ± 0.08, 14.79 ± 1.19, and 42.74 ± 2.23 for normal group, control group, TP, TP@Pd, and TP@Pd+NIR, respectively (Figure 5G). Similarly, the MFI of CD31 expression followed the order: TP@Pd+NIR (41.83 ± 0.54) >TP@Pd (32.08 ± 0.40) >TP (28.02 ± 3.13) >control group (27.08 ± 1.99) >normal group (26.38 ± 0.26) (Figure 5I).

LPS is a unique component of the cell wall of gram‐negative bacteria, also known as endotoxins. Infection with LPS can lead to multiple organ dysfunction syndrome, and also sepsis. LPS can induce the host's immune response through signal transduction pathways, and stimulate immune cells to produce a huge number of inflammatory cytokines.^[^ ^42^ ^]^ Due to the similar pathological characteristics of ALI patients, LPS intervention is commonly considered to be applied for creating in vitro and in vivo ALI models.^[^ ^43^ ^]^ Previous studies have also confirmed that HSP70 and CD31 play crucial roles in restoring the normal functions of mutated proteins, and their high expression help to promoting soft tissue repair.^[^ ^44^ ^]^ The results above indicated that TP and TP@Pd exhibited the favorable biocompatibility and hemocompatibility within certain concentration ranges, highlighting their feasibility for clinical application. LPS induced oxidative stress could lead to cell death, but TP@Pd, with enhanced ROS scavenging capacities, had been shown to more efficiently prevent LPS induced cell death compared to TP. Notably, these capabilities were further enhanced by NIR irradiation, which accelerated the movement of ultra‐small Pd particles, improving their ROS scavenging abilities.^[^ ^45^ ^]^ Although the zeta potential of TP and TP@Pd were positive and negative respectively, both were efficiently taken up by cells due to their nanoscale size effects. The cellular uptake of TP@Pd was significantly enhanced compared to TP, and despite the change in zeta potential from positive to negative, ultra‐small Pd loading significantly improved the dispersion ability of TP@Pd, preventing aggregation and facilitating better cellular uptake. Furthermore, considering the multiple catalytic activities of nanozymes, the results demonstrated that TP@Pd exhibited more efficient antioxidant and anti‐inflammatory capacities in LPS stimulated macrophages compared to TP. The presence of ultra‐small Pd enhanced the ROS scavenging ability of TP@Pd. Most significantly, TP@Pd+NIR displayed the optimal ROS scavenging abilities and was efficiently taken up by cells, providing the most effective protection against LPS induced oxidative stress. Thus, in turn, lowered intracellular ROS levels, downregulated inflammatory factor expression, upregulated antioxidant factor expression, induced M2 polarization of macrophages, and improved the expression levels of HSP70 and CD31, all of which contributed to alleviating ALI.

### 2.4 Related Therapeutic Mechanisms Investigation

According to previous studies, nanozymes, including nanosized Pd, exhibit anti‐inflammatory effects due to their widely reported multiple catalytic functions, such as catalase (CAT) and superoxide dismutase (SOD) activities, which effectively scavenge extracellular and intracellular ROS levels.^[^ ^46^ ^]^ By scavenging ROS, nanozymes can alleviate cellular oxidative stress, thereby inhibiting the NF‐κB signaling pathway.^[^ ^47^ ^]^ Moreover, ROS not only directly stimulates NF‐κB activation but also participates in the activation of upstream pathways, such as IκB kinase (IKK), and mitogen‐activated protein kinase (MAPK) pathways.^[^ ^48^ ^]^ It had been demonstrated that TP@Pd+NIR could efficiently alleviate ALI in vitro by reprogramming redox homeostasis. To investigate the changes in gene expression levels in LPS induced macrophages with or without treatment, whole transcriptome sequencing was performed. As shown in Figure S19 (Supporting Information), the expressional correlation between three typical antioxidant enzymes (SOD2, CAT, and Nqo1), and the differentially expressed genes (DEGs) were visualized. The analysis revealed that DEGs positively correlated with antioxidant enzymes were highly expressed, while those negatively correlated were expressed at lower levels in TP@Pd+NIR treated macrophages, suggesting a potential antioxidant pathway mediated by TP@Pd+NIR (Figure S20, Supporting Information).

Meanwhile, KEGG enrichment analysis was conducted to explore the biological functions of the DEGs (Figure S21, Supporting Information). Based on the Gene Ontology (GO) database, each DEGs were annotated with its respective molecular pathway, and NF‐κB pathway related DEGs were selected as genes of interest. Differential analysis identified 149 DEGs between control group and TP@Pd+NIR (Figure 5J,K). By annotating the molecular functions of these 149 DEGs, Foxj1, Bcl2ala and HSPBl were identified as NF‐κB pathway related DEGs. Subsequently, NF‐κB pathway related genes were retrieved from the KEGG database, followed by ensemble ID conversion and intersection with all identified genes (Figure S22, Supporting Information). The resulting 42 genes were subjected to Pearson correlation analysis with NF‐κB pathway related DEGs (correlation coefficient >0.2 and *p*‐value < 0.05), constructing an expression relevance network (Figure 5L). From the analysis, it revealed a strong negative correlation between IκBα and HSPBl, as well as a strong positive correlation between IκBα and Foxj1 (Figure 5M). These findings provided the evidence that NF‐κB pathway might be a potential mechanism underlying ALI therapy mediated by TP@Pd+NIR.

The NF‐κB signaling pathway regulates gene expression, and influences various biological processes. Dysregulation of this pathway is associated with multiple human diseases including cancer, inflammation, viral infections, and sepsis.^[^ ^49^ ^]^ Therefore, inhibiting NF‐κB signaling pathway can help to alleviate pulmonary inflammation storms, and promote lung injury repair. Western blotting (WB) was performed to characterize the representative proteins expression involved in NF‐κB pathway. As displayed in Figure S23 (Supporting Information) and Figure 5N, the IκBα expression level decreased in control group compared to normal group, but significantly increased in all treated groups. However, no significant differences were observed in p65 expression levels among all groups. Meanwhile, the phosphorylation levels of IκBα (p‐IκBα) and p65 (p‐p65) were highest in control group compared to all treated groups. Furthermore, the expression levels of p‐IκBα decreased in the order of control group, normal group, TP, TP@Pd and TP@Pd+NIR, while p‐p65 expression levels followed the order: control group >TP >TP@Pd >TP@Pd+NIR >normal group. The statistical analysis revealed that the relative IκBα expression level decreased to 0.47 ± 0.04 in control group compared to 0.60 ± 0.02 in normal group, but it increased to 0.83 ± 0.01, 0.97 ± 0.03 and 1.12 ± 0.02 in TP, TP@Pd and TP@Pd+NIR respectively (Figure S24A, Supporting Information). However, the p65 expression levels were approximately 0.91 ± 0.08 across all groups (Figure S24C, Supporting Information). Significantly, the expression levels of p‐IκBα and p‐p65 were reduced to 0.61 ± 0.08 and 0.80 ± 0.03, respectively, in TP@Pd+NIR compared to 1.27 ± 0.02 and 1.28 ± 0.01 in control group (Figure S24B,D, Supporting Information). These findings indicated that TP@Pd effectively inhibited the NF‐κB signaling pathway by downregulating p‐p65 and p‐IκBα expression levels, further achieving anti‐inflammatory effects and promoting tissue repair. These effects were significantly enhanced by TP@Pd+NIR. To identify the key component responsible for inhibiting NF‐κB signaling pathway, WB analysis was also conducted. As shown in Figure S26 (Supporting Information), IκBα expression levels were reduced in control group compared to normal group but increased in TP, Pd and NIR. Conversely, p‐IκBα expression levels were higher in control group and NIR compared to normal group, but lower in Pd and TP. Additionally, p65 expression levels decreased, and p‐p65 expression levels increased in control group compared to normal group. In contrast, p65 expression levels significantly increased, and p‐p65 expression levels markedly decreased in Pd, followed by TP. As shown in statistical analysis, the relative IκBα/GAPDH ratio was 0.85 ± 0.00 in normal group, decreased to 0.25 ± 0.00 in control group, and increased to 0.72 ± 0.01, 0.64 ± 0.00 and 0.53 ± 0.00 in Pd, TP and NIR, respectively (Figure S27A, Supporting Information). Conversely, the relative p‐IκBα/GAPDH ratio was 0.60 ± 0.01 in normal group, increased to 0.75 ± 0.01 and 0.85 ± 0.02 in control group and NIR, and reduced to 0.68 ± 0.01 and 0.56 ± 0.01 in TP and Pd, respectively (Figure S27B, Supporting Information). For the p65/GAPDH and p‐p65/GAPDH ratios, the respective values were 1.05 ± 0.01 and 0.47 ± 0.01 (normal group), 0.94 ± 0.00 and 0.68 ± 0.03 (control group), 1.20 ± 0.01 and 0.26 ± 0.01 (Pd), 1.17 ± 0.01 and 0.34 ± 0.01 (TP), and 1.09 ± 0.01 and 0.48 ± 0.01 (NIR) (Figure S27C,D, Supporting Information). These results confirmed that Pd played a key role in ROS scavenging and NF‐κB signaling pathway inhibition. TP also contributed to the inhibition of NF‐κB pathway, though less effectively than Pd. Notably, NIR irradiation alone had minimal or negligible effects on NF‐κB signaling pathway.

### 2.5 In Vivo ALI Therapy Evaluation

**Figure 6:**
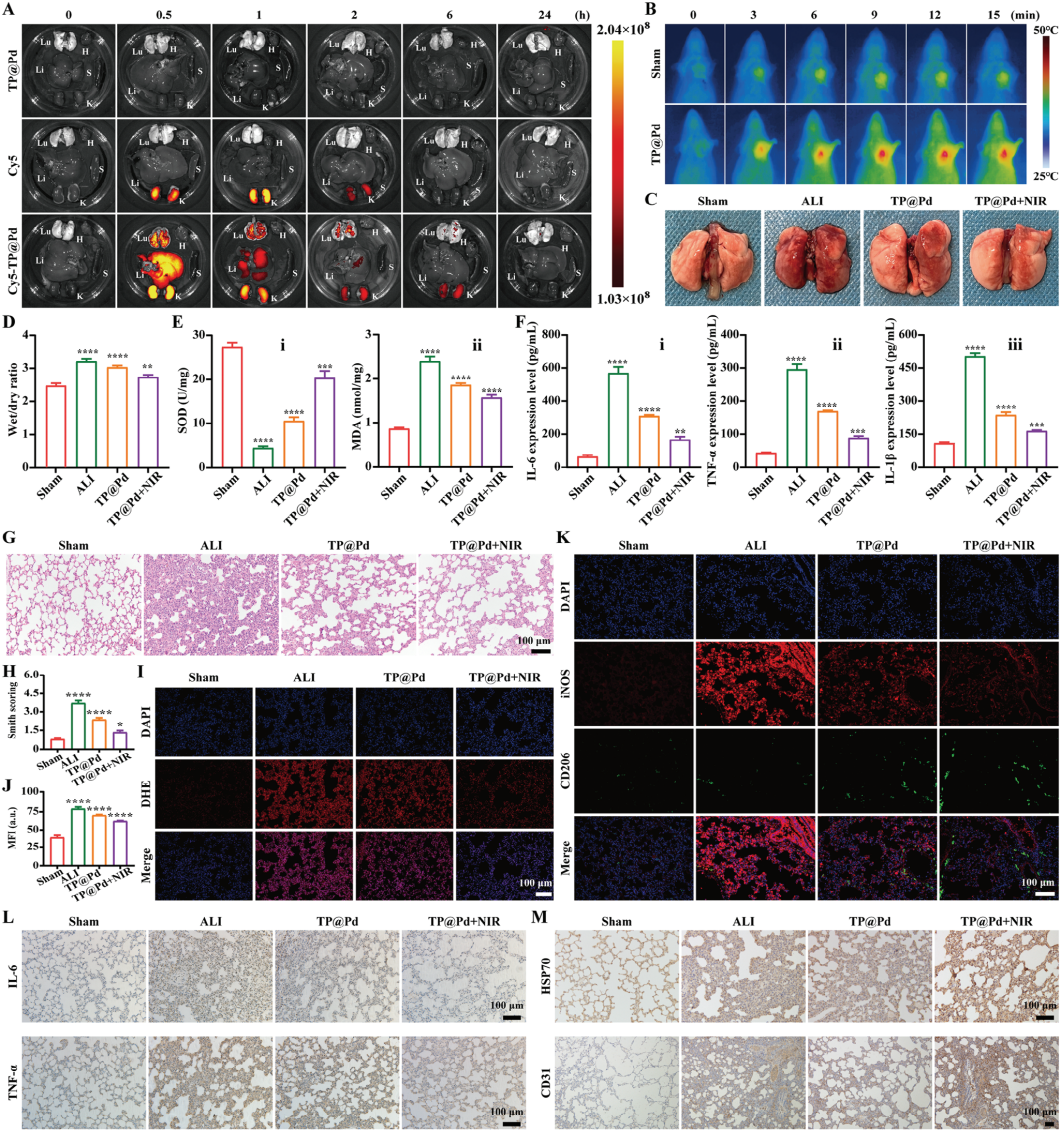
In vivo ALI therapy evaluation. A) In vivo bio‐distribution of NPs. The fluorescent images of major isolated organs (heart (H), liver (Li), spleen (S), lung (Lu), and kidney (K)) at predetermined time points (0, 0.5, 1, 2, 6, and 24 h) by IVIS. The corresponding groups were: rats injected with TP@Pd (TP@Pd), Cy5 (Cy5), and Cy5‐TP@Pd (Cy5‐TP@Pd). B) In vivo photothermal images of treated rats versus time under NIR irradiation (1 W cm^−2^). C) Macroscopic observation of lung tissue of treated rats. D) The wet/dry ratio of lung tissue of treated rats. E) The SOD (i) and MDA (ii) levels of lung homogenate of treated rats by the corresponding testing kits. F) Inflammatory factors (IL‐6 (i), TNF‐α (ii), and IL‐1β (iii)) expression levels of lung homogenate of treated rats by ELISA. G) H&E staining images of lung tissue of treated rats and the corresponding Smith score (H). I) ROS levels of lung tissue of treated rats and the corresponding quantified results (J). K) iNOS and CD206 co‐immunostaining images of lung tissue of treated rats. L) IL‐6 and TNF‐α expression levels of lung tissue of treated rats. M) HSP70 and CD31 expression levels of lung tissue of treated rats. The corresponding groups were: rats without treatment (sham group), LPS induced rats with saline injection (ALI group), LPS induced rats with TP@Pd injection (TP@Pd), and LPS induced rats with TP@Pd injection and NIR irradiation (TP@Pd+NIR). (“*” symbol compared with sham group, *p < 0.05, **p < 0.01, ***p < 0.001 and ****p < 0.0001).

In clinical applications, biomedicines must enrich in the target tissue, remain there for a specific duration, and eventually degrade and be eliminated from the body. From in vitro experiments, it was demonstrated that TP@Pd exhibited superior ROS scavenging capacities, and higher cellular uptake efficiency compared to TP. Therefore, TP was not considered for in vivo therapy evaluation. To investigate the biodistribution of TP@Pd, in vivo animal imaging systems (IVIS, Pekin Elmer, USA) was used to monitor the fluorescence intensity changes in the major organs of Sprague dawley (SD) rats. As displayed in **Figure** **6**A, after intravenous (IV) injection, no fluorescence was observed for TP@Pd alone, indicating that TP@Pd did not contribute any fluorescence detectable by IVIS. In contrast, for Cy5 alone, fluorescence was only observed in the kidney (K), with a strong signal at 0.5 h that disappeared after 2 h. For Cy5‐TP@Pd, fluorescence was apparent in the liver (Li), lung (Lu), and kidney (K), while no fluorescence was detected in the heart (H) or spleen (S) at 0.5 h. Although the fluorescence intensity of Cy5‐TP@Pd gradually decreased over time, it remained detectable in the lung (Lu) for more than 6 h, and disappeared completely at 24 h. For the liver (Li) and kidney (K), fluorescence disappeared entirely by 24 h, indicating that TP@Pd was eventually cleared from the body. Statistical analysis showed that no fluorescent signal was present in the lung (Lu) at the beginning of the experiment. At 0.5 h, Cy5‐TP@Pd exhibited a strong fluorescent signal in the lung, with the highest MFI of 6.60 × 10⁹, which gradually decreased over time and disappeared entirely at 24 h (Figure S28, Supporting Information). These results confirmed that TP@Pd could enrich in the lung, retain there for ≈6 h, and eventually be cleared, highlighting its potential for clinical applications. Specifically, the differences in fluorescence between Cy5 and Cy5‐TP@Pd indicated that the gradually decreasing fluorescence of Cy5‐TP@Pd was due to the degradation of TP@Pd rather than fluorescence quenching of Cy5. The in vivo biodistribution of passively targeted NPs primarily depends on their size. NPs smaller than 50 nm are rapidly cleared by the kidney during blood circulation. In contrast, NPs ≈200 nm can retain a significant amount in the liver, lung, and kidney, where they gradually degrade.^[^ ^50^ ^]^ Studies had shown that NPs larger than 100 nm could effectively remain in the lung for treating lung related diseases.^[^ ^32^, ^51^ ^]^ Thus, TP@Pd with its size enabling retention in the lung for ≈6 h following IV injection, holds promise for ALI therapy. It is eventually cleared, reducing the risk of additional cytotoxicity.

NIR irradiation is considered an effective strategy to achieve controllable therapy at disease sites with minimal side effects. Given its tissue penetration depth of ≈1 cm, NIR light (808 nm) was utilized to evaluate the feasibility of in vivo PTT. As shown in Figure 6B, in sham group, the temperature at the lung site increased slightly at the beginning, and stabilized at 38.1 °C after 15 min of NIR irradiation. For TP@Pd, NIR light was applied to the lung site of SD rats after IV injection for 0.5 hours, when TP@Pd was confirmed to be highly enriched in the lung based on IVIS imaging. The corresponding temperature at the lung site increased significantly over time, reaching 47.8 °C after 15 min of irradiation (Figure S29, Supporting Information). This temperature is below the threshold for irreversible tissue damage (50 °C),^[^ ^36^, ^52^ ^]^ ensuring that no harm was caused to normal tissues. The observed photothermal effects were entirely attributed to the photosensitizer (TP@Pd) rather than the surrounding environment. These results confirmed that TP@Pd exhibited excellent in vivo photothermal effects, demonstrating its feasibility for PTT in treating ALI.

For clinic application, in vivo biosafety is a critical factor that must be carefully evaluated. On the 7th day after administration, the body weight, blood indicators, and pathology of major organs were observed and assessed. As illustrated in Figure S30 (Supporting Information), the body weight of normal rats (sham group) slightly increased versus time, exhibiting a similar trend to that of TP@Pd treated rats. Additionally, no obvious differences were observed between sham group and TP@Pd in the blood routine indicators (WBC, RBC, HGB, and PLT), liver and kidney function indicators (AST, ALT, CREA, and UREA), myocardial enzyme indicators (CK and CK‐MB), or coagulation indicators (PT, INR, APTT, TT, and FIB) following IV injection of TP@Pd after 7 days (Figure S31 and Table S7, Supporting Information). Furthermore, from the images of hematoxylin and eosin (H&E) staining, it revealed no obvious damages to major organs including the heart, liver, spleen, lung and kidney in TP@Pd, consistent with the findings in sham group (Figure S32, Supporting Information). These results demonstrated that this therapeutic strategy possessed favorable in vivo biosafety and did not induce additional side effects.

It is well known that LPS intervention can cause systemic organ failure. To minimize damage to other organs, intratracheal instillation of LPS was used to establish an ALI animal model.^[^ ^53^ ^]^ Based on the in vivo biodistribution and photothermal effects, IV injection was applied for the administration of in vivo ALI therapy. After establishing the animal model, ALI rats were IV injected with the respective dose solutions. And the blood samples and major organs including heart, liver, spleen, lung, and kidney were collected 24 h after administration. Initially, blood indicators related to blood routine, liver and kidney function, myocardial enzymes, and coagulation were tested. As shown in Figure S33 and Table S8 (Supporting Information), no significant differences happened among all groups except for FIB, WBC, RBC, HGB, PLT, AST, and UREA, which were significantly increased in ALI group, but decreased following treatment with TP@Pd and TP@Pd+NIR. Additionally, the PLT levels, which decreased in ALI group compared to sham group, increased after treatment. Similarly, the expression levels of IL‐6, TNF‐α, and IL‐1β in blood serum were analyzed using ELISA. As illustrated in Figure S34 (Supporting Information), compared to sham group (5.86 ± 2.97, 18.90 ± 4.86, and 14.57 ± 4.10), ALI group exhibited significantly elevated levels of inflammatory factors (113.40 ± 6.21, 248.02 ± 8.86 and 50.84 ± 4.99). These levels were reduced by TP@Pd treatment (82.03 ± 5.91, 150.05 ± 18.20, and 27.64 ± 3.53), and further decreased by TP@Pd+NIR treatment (24.68 ± 1.67, 121.00 ± 7.39, and 16.58 ± 2.53). Among the treatments, TP@Pd+NIR most efficiently lowered IL‐6, TNF‐α and IL‐1β expression levels in serum. Meanwhile, major organs, except for the lungs, were evaluated through gross examination and H&E staining. It was observed that there were almost no damages to the heart, liver, spleen, or kidney in ALI group, TP@Pd and TP@Pd+NIR, comparable to those in sham group (Figures S35 and S36, Supporting Information). The ALI animal model was successfully established using intratracheal administration of LPS for 30 min, rather than cecal ligation surgery, followed by IV injection for in vivo therapy. And the therapeutic evaluations were conducted within 24 h. These results confirmed the successful establishment of ALI animal model, absence of damage to other organs, and lack of additional side effects associated with TP@Pd+NIR treatment strategy.

Significantly, the lung tissue was the main research target for in vivo therapeutic efficacy. Initially, the gross appearance of the lung was observed. By analyzing the color, texture and morphology of lung tissue, the therapeutic effects were assessed. As shown in Figure 6C, the lungs of sham group were pale pink, indicating smooth texture, good elasticity, and the absence of congestion or edema existed in normal lung. However, the lung surface in ALI group appeared dark red and congested, with visible focal bleeding and necrosis. Compared to ALI group, TP@Pd and TP@Pd+NIR exhibited a significant reduction in lesions, with TP@Pd+NIR showing almost no visible lesions. In addition, the wet‐to‐dry (w/d) weight ratio of the lungs was measured. As illustrated in Figure 6D, the w/d weight ratio was 2.49 ± 0.06 for sham group, significantly increased to 3.21 ± 0.08 in ALI group, indicating the presence of pulmonary edema caused by LPS intervention. Treatment with TP@Pd and TP@Pd+NIR effectively reduced the w/d weight ratio to 3.03 ± 0.05 and 2.74 ± 0.06, respectively. SOD activity is a well‐established indicator of oxidative stress in tissues.^[^ ^54^ ^]^ And malondialdehyde (MDA) content is a stable metabolite of lipid peroxidation, which could reflect the level of free radicals, and the lipid peroxidation reactions caused by free radicals.^[^ ^55^ ^]^ Both parameters can indirectly indicate the degree of tissue damage resulting from oxidative stress. Next, the SOD activity and MDA content in lung homogenates were analyzed. For SOD activity, sham group exhibited high levels of 27.36 ± 1.03 U mg^−1^, which significantly decreased to 4.43 ± 0.39 U mg^−1^ in ALI group. Treatment with TP@Pd and TP@Pd+NIR restored SOD activity to 10.58 ± 0.83, and 20.39 ± 1.53 U mg^−1^ respectively (i in Figure 6E). Conversely, MDA content was low in sham group (0.87 ± 0.02 nmol mg^−1^), but increased to 2.40 ± 0.11 nmol mg^−1^ in ALI group. Following treatment, MDA content was reduced to 1.86 ± 0.04 and 1.57 ± 0.06 nmol mg^−1^ for TP@Pd and TP@Pd+NIR respectively (ii in Figure 6E). Furthermore, the IL‐6, TNF‐α, and IL‐1β expression levels in lung tissues were analyzed using ELISA. As illustrated in Figure 6F (i), the IL‐6 level in the lung homogenate was 64.95 ± 8.83 pg mL^−1^ in sham group but increased to 569.36 ± 37.36 pg mL^−1^ in ALI group. Treatment with TP@Pd and TP@Pd+NIR significantly lowered IL‐6 levels to 309.61 ± 6.89 and 166.89 ± 17.58 pg mL^−1^ respectively. A similar trend was observed for TNF‐α and IL‐1β expression levels. In ALI group, TNF‐α and IL‐1β levels were 296.09 ± 15.86 and 504.31 ± 13.53 pg mL^−1^ respectively, while these levels were reduced to 89.05 ± 5.20 and 164.76 ± 4.02 pg mL^−1^ respectively in TP@Pd+NIR (ii and iii in Figure 6F).

Furthermore, the pathological characteristics of lung tissue were also evaluated. From H&E staining result, it revealed that the lung tissue structure in sham group was complete and clear, with no capillary dilation or congestion in the alveolar septa, and no signs of inflammatory cell infiltration or edema. In contrast, ALI group displayed severe ALI symptoms including the destruction of alveolar structures, thickening of alveolar walls, infiltration of a huge number of inflammatory cells, and extensive exudation of inflammatory cells into the alveolar cavity, confirming the successful establishment of ALI animal model. Treatment with TP@Pd and TP@Pd+NIR significantly alleviated these symptoms. Notably, in TP@Pd+NIR, the alveolar structure was relatively intact, with minimal infiltration of inflammatory cells and thinner alveolar walls (Figure 6G). After Smith scoring, the results were 0.77 ± 0.12 for sham group, 3.73 ± 0.21 for ALI group, 2.37 ± 0.15 for TP@Pd, and 1.33 ± 0.15 for TP@Pd+NIR (Figure 6H). Additionally, ROS levels in lung tissue were analyzed using DHE staining. Stronger fluorescence indicated higher ROS levels in the lung tissue. As shown in Figure 6I, sham group exhibited low red fluorescence, indicating low ROS levels, while ALI group displayed significantly enhanced red fluorescence. Treatment with TP@Pd and TP@Pd+NIR effectively reduced the ROS levels in the lung tissue of ALI rats. Statistical analysis showed that the MFI was 39.43 ± 3.03 in sham group, which increased to 78.54 ± 2.47 in ALI group. Following treatment, the MFI decreased to 69.78 ± 1.02 for TP@Pd, and 61.46 ± 0.80 for TP@Pd+NIR respectively (Figure 6J).

Equally, macrophage polarization was analyzed through co‐immunofluorescent staining, where green fluorescence corresponded to CD206 expression, and red fluorescence indicated the expression of iNOS. In sham group, macrophages in lung tissue were in the M0 state, with relatively low expression levels of iNOS (23.87 ± 0.82) and CD206 (82.42 ± 3.69). However, in ALI group, the iNOS expression level significantly increased to 114.53 ± 3.40, and a slight increase in CD206 expression (100.08 ± 1.93) was observed, indicating the M1 polarization of macrophages. Notably, treatment with TP@Pd and TP@Pd+NIR reduced iNOS expression level, and increased CD206 expression level, indicating a shift from M1 to M2 polarization (Figure 6K; Figure S37, Supporting Information). To further quantify macrophage polarization, RT‐qPCR was used to detect iNOS and CD206 expression levels in lung tissue. As shown in Figure S38 (Supporting Information), iNOS gene expression in sham group was low (1.00 ± 0.04), but significantly increased in ALI group (17.50 ± 0.40). Treatment with TP@Pd and TP@Pd+NIR reduced iNOS expression levels to 11.83 ± 0.64 and 2.36 ± 0.17 respectively (Figure S36A, Supporting Information). Conversely, CD206 expression was relatively low in both sham group (1.05 ± 0.41) and ALI group (0.93 ± 0.26), but increased significantly with TP@Pd (15.61 ± 1.64) and TP@Pd+NIR (20.68 ± 2.66) treatment. The low iNOS and high CD206 expression levels in TP@Pd and TP@Pd+NIR indicated the M2 polarization of macrophages in lung tissue after therapy. Additionally, the number of CD4^+^ and CD8^+^ T cells in blood and lung tissue was analyzed. In blood, CD8^+^ T cells accounted for 50.07 ± 0.21% in sham group, and 50.43 ± 0.42% in ALI group, decreasing to 40.23 ± 0.15% and 33.63 ± 0.95% in TP@Pd and TP@Pd+NIR respectively. Conversely, CD4^+^ T cells were 45.63 ± 0.15% in sham group, and 45.00 ± 0.66% in ALI group, and increased sequentially in TP@Pd (54.03 ± 0.49%) and TP@Pd+NIR (60.27 ± 1.08%). The calculated CD4^+^/CD8^+^ ratio in blood was 0.91 ± 0.01, 0.89 ± 0.02, 1.34 ± 0.01 and 1.79 ± 0.08 for sham group, ALI group, TP@Pd and TP@Pd+NIR, respectively (Figure S40, Supporting Information). T cell populations in lung tissue were also evaluated using flow cytometry and immunostaining. As shown in Figure S41 (Supporting Information), CD4^+^ and CD8^+^ T cells counts decreased in ALI group (6.99 ± 0.37% and 13.60 ± 0.46%) compared to sham group (10.11 ± 1.55% and 16.07 ± 1.86%). Treatment with TP@Pd increased CD4^+^ T cells to 12.57 ± 0.25%, and slightly increased CD8^+^ T cells to 15.07 ± 0.25%, while TP@Pd+NIR increased CD4^+^ T cells significantly to 22.87 ± 0.29%, and CD8^+^ T cells significantly to 23.23 ± 0.23%. The CD4^+^/CD8^+^ ratio in lung tissue was 0.64 ± 0.18 and 0.51 ± 0.03 for sham and ALI groups, increasing to 0.83 ± 0.03 and 0.98 ± 0.02 for TP@Pd and TP@Pd+NIR respectively (Figure S42, Supporting Information). Immunostaining further corroborated these findings. As shown in Figure S43 (Supporting Information), green fluorescence (CD4^+^ T cells) decreased, and red fluorescence (CD8^+^ T cells) increased in ALI group compared to sham group. Treatment with TP@Pd, especially TP@Pd+NIR, resulted in the increased green fluorescence and decreased red fluorescence, indicating an increase in CD4^+^ T cells, and a decrease in CD8^+^ T cells. Statistical analysis showed that the CD4^+^/CD8^+^ ratio was 0.81 ± 0.09 in sham group, decreased to 0.34 ± 0.09 in ALI group, and increased to 0.96 ± 0.08 and 1.62 ± 0.07 in TP@Pd and TP@Pd+NIR respectively (Figure S44, Supporting Information). In conclusion, increased CD206 and decreased iNOS expression levels, along with an increased number of CD4^+^ T cells, and a higher CD4^+^/CD8^+^ ratio in blood and lung tissue, demonstrated the activation of immunoregulation, particularly with TP@Pd+NIR treatment, contributing to ALI therapy.

Finally, the IL‐6 and TNF‐α expression levels in lung tissue were investigated using immunohistochemical staining. As shown in Figure 6L and Figure S45 (Supporting Information), ALI group exhibited the highest IL‐6 and TNF‐α expression levels, with the average optical densities (AOD) of 0.30 ± 0.02 and 0.24 ± 0.03, respectively. However, these expression levels decreased following treatments, with the AOD of 0.26 ± 0.01 and 0.20 ± 0.01 for TP@Pd, and 0.16 ± 0.00 and 0.14 ± 0.00 for TP@Pd+NIR. Notably, the AOD for IL‐6 and TNF‐α in TP@Pd+NIR were almost comparable to those of sham group (0.12 ± 0.01 and 0.07 ± 0.01 respectively). In addition to antioxidant and anti‐inflammatory effects, tissue repair capabilities were also evaluated using immunohistochemical staining. As displayed in Figure 6M and Figure S46 (Supporting Information), the expression levels of HSP70 and CD31 were significantly higher in TP@Pd+NIR, with the AOD of 0.44 ± 0.00 and 0.32 ± 0.01 respectively, compared to relatively lower levels observed in other groups.

Altogether, the results demonstrated the optimal in vivo ALI therapeutic effect of TP@Pd+NIR. Due to its unique nanosized framework, TP@Pd was not only enriched at the lung site following IV injection, but also retained there for several hours. Importantly, it ultimately degraded, and was cleared from the body, meeting the primary requirements of a biomedicine for diseases therapy. The destabilization of C═N groups contributed to the degradation of TP, ensuring its biocompatibility. Moreover, TP and TP@Pd exhibited favorable photothermal effects. Specifically, TP@Pd demonstrated feasibility for in vivo PTT of lung related diseases by heating the tissue to 47.8 °C under NIR irradiation (1 W cm^−2^) for 15 min, sufficient to maintain the optimal temperature (≈45.0 °C) for tissue repair. Additionally, as TP@Pd is composed solely of a stable COF and ultra‐small Pd, it demonstrated favorable in vivo biosafety, with no side effects existed on body weight, blood indicators or major organs, underscoring its potential for clinical application. LPS intervention via intratracheal instillation was employed to establish precise ALI animal models, ensuring lung specific injury while avoiding damage to other organs. The gross view of major organs confirmed the successful ALI animal model establishment. IVIS imaging and in vivo photothermal effects provided further evidences supporting the efficacy of ALI therapy via IV injection of TP@Pd combined with NIR irradiation. Previous studies had demonstrated that COFs could act as nanozymes with ROS scavenging capacity, and serve as effective photothermal agents for photothermal conversion.^[^ ^56^ ^]^ Compared to TP alone, the addition of ultra‐small Pd significantly enhanced ROS scavenging capacity in TP@Pd. Notably, when combined with NIR irradiation, TP@Pd+NIR exhibited remarkably improved ROS scavenging abilities, effectively downregulating IL‐6, TNF‐α, and IL‐1β in blood serum and lung tissue, reducing MDA content in lung tissue, increasing SOD activity, and alleviating oxidative damage in the lungs. In particular, TP@Pd+NIR demonstrated exceptional ROS scavenging and immunoregulation activation capabilities by reducing ROS levels in lung tissue, downregulating iNOS expression, upregulating CD206 expression, increasing the number of CD4^+^ T cells, and elevating CD4^+^/CD8^+^ ratio. Furthermore, under NIR irradiation, TP@Pd+NIR significantly promoted the expression of HSP70 and CD31, thereby accelerating lung tissue repair. Collectively, these results provided compelling evidence that TP@Pd+NIR efficiently achieved in vivo ALI immunotherapy by reprogramming redox homeostasis.

## 3 Conclusion

In summary, we successfully developed a novel COF based nanozyme (TP@Pd) combined with NIR irradiation for synergistically enhanced ALI immunotherapy. TP@Pd was designed by incorporating ultra‐small Pd into the COF framework, enabling the dual functionality of ROS scavenging and PTT. Enhanced ROS scavenging capacities were achieved through the combination of ultra‐small Pd loading and NIR irradiation. Benefiting from the integration of excellent ROS scavenging and photothermal enhancement, TP@Pd+NIR acted as an efficient synergistic strategy for ALI immunotherapy by downregulating inflammatory cytokine expression, reducing intracellular ROS levels, and inducing M2 polarization of macrophages. Furthermore, TP@Pd demonstrated enhanced lung accumulation and retention, coupled with gradual degradation over time, showcasing excellent biocompatibility and hemocompatibility. Notably, TP@Pd+NIR exhibited significant capabilities in reducing lung inflammation, activating immunoregulation, alleviating pulmonary oxidative damage, and promoting lung tissue repair. Mechanistically, these synergistic effects amplified ALI therapeutic efficacy by efficiently inhibiting NF‐κB signaling pathway, and reprogramming redox homeostasis. We believed the findings of this study presented a highly valuable strategy for the synergistic therapy of ROS related diseases in clinical settings.

## 4 Experimental Section

### Material and Chemicals

TAPA (C_18_H_18_N_4_, 98%, Mw = 290.36), PA (C_8_H_6_O_2_, 98%, Mw = 134.13) and L‐ascorbic acid (C_6_H_8_O_6_, 99.99%, Mw = 176.12) were purchased from Macklin (China). LPS (≥97%) was supplied by Sigma‐Aldrich (St. Louis, US), and H_2_O_2_ (30% w/w, Mw = 34.01) was obtained from Junobio (Nanning, China). Methanol (CH_4_O, 99.5%, Mw = 32.04), anhydrous ethanol (C_2_H_6_O, ≥99.7%, Mw = 46.07), sodium tetrachloropalladate (Na_2_PdCl_4_, 98%, Mw = 294.21) and acetic acid (CH_3_CO_2_H, ≥99.7%, Mw = 60.05) were procured from Aladdin (Shanghai, China). All chemicals were applied directly without further purification.

### Preparation and Characterization of TP@Pd

TP@Pd was synthesized via a simple hydrothermal reaction of TAPA and PA, followed by in‐situ reduction of ultra‐small Pd. Briefly, 60 mg of TAPA and 30 mg of PA were dissolved in a solution containing 30 mL of methanol and 2 mL of acetic acid. After ultrasonic dispersion for 15 min, the mixture was transferred into a reactor and maintained at 100 °C overnight. The resulting mixture was centrifuged at 12 000 rpm, and re‐dispersed in ethanol for three times, and TP was isolated and obtained after vacuum drying. Subsequently, 50 mg of TP was dispersed in 100 mL of ethanol, followed by the addition of 5 mL of Na₂PdCl₄ solution (10 mg mL^−1^). After magnetic stirring for 1 h, 2 mL of L‐ascorbic acid solution (20 mg/mL) was added slowly, and the final mixture was allowed to react for 6 h. Finally, TP@Pd was purified by repeated centrifugation and re‐dispersion for three times, and collected after vacuum drying.

TP and TP@Pd were characterized using UV‐vis (Shimadzu, Japan), FTIR (Shimadzu, Japan), Raman spectroscopy (HORIBA HR Evolution, France), and XRD (MiniFlex 600, Japan) to investigate their chemical structure, molecular structure, and crystallization structure, respectively. The morphology, elemental distribution, and 3D structure of TP and TP@Pd were examined using TEM (Hitachi, Japan) coupled with energy‐dispersive X ray spectroscopy (EDS, Hitachi, Japan) and AFM (Bruker, USA). XPS (ESCALAB 250XI+, USA) and ICP‐OES (Varian, USA) were employed to analyze element composition and metal element content, respectively. The zeta potential of all samples was measured using a zeta sizer (Nano ZS90, Malvern, UK). Additionally, the thermal stability of the nanoparticles was evaluated by TGA (STD650, TA, USA) from 20 to 800 °C under a nitrogen atmosphere, and their specific surface area was determined using the BET gas adsorption method (Micromeritics ASAP, USA). Finally, the degradability of TP@Pd was assessed by immersing the samples in PBS (pH 7.4, Solarbio, China) or pH 5.0. At predetermined time points (0, 0.5, 1, 2, 4, 8, 12, and 24 hours), the morphology was observed by TEM, while changes in size and its PDI, and zeta potential were measured using a zeta sizer.

### Dispersion and In Vitro Photothermal Effects Investigation

To investigate their dispersion and stability, NPs were dispersed in PBS, FBS (Every Green, China), DMEM (Gibco, USA) or 5 mM H₂O₂ in PBS and observed using a camera at different time points (0, 0.5, 1, 2, 4, 8, 24, and 48 h). Specifically, the stability of TP and TP@Pd was further evaluated by dispersing them in the blood serum of SD rats and observing their behavior with a camera at the same time points. For in vitro photothermal effects, NPs with different concentrations (0, 100, 200, or 500 µg mL^−1^) were subjected to NIR irradiation at various power intensities (808 nm, 0.5, 1, 1.5, or 2 W cm^−2^) for 15 min. In particular, photothermal stability testing was performed by subjecting 200 µg mL^−1^ TP@Pd to five repeated “on” and “off” cycles under NIR light (808 nm, 1 W cm^−2^). Corresponding images and temperature data were recorded using a thermal imaging camera (FLIR, USA).

### ROS Scavenging Capacities Testing

At the beginning, ESR (Bruker A300, Germany) was employed to evaluate the ROS scavenging ability of NPs. For ·OH testing, 5‐tert‐butoxycarbonyl 5‐methyl‐1‐pyrroline‐N‐oxide (BMPO, 100 mM) was used as the working solution. After mixing with 200 µg mL^−1^ NPs for 10 min, the corresponding signal was recorded using ESR. Similarly, xanthione (10 mM) and xanthione oxidase (XOD, 1 U mL^−1^), as well as 2, 2, 6, 6‐Tetramethylpiperidine (TEMPONE, 100 mM) were used as the working solutions for ·O₂⁻ and ¹O₂ testing, respectively. For TP@Pd+NIR, NIR irradiation (808 nm, 1 W cm^−2^) was applied for 10 min after mixing the working solution with NPs before ESR testing. Additionally, ROS testing kits, including CAT activity kits (Beyotime, China), hydroxyl radical and superoxide anion scavenging kits (Solarbio, China), were used to assess the H₂O₂, ·OH, and ·O₂^⁻^ scavenging capacities of NPs, following the manufacturers' instructions. Specifically, for H₂O₂ scavenging ability, TP and TP@Pd at various concentrations (0, 50, 100, 200, and 500 µg mL^−1^) were dispersed in the working solution. After stirring magnetically for 30 min, the supernatant of the solutions was measured at 520 nm using a microplate reader. Similarly, the OD of the supernatant was recorded at 536 nm and 530 nm to determine the ·OH and ·O₂^⁻^ scavenging capacities, respectively. For TP@Pd+NIR, NIR irradiation (1 W cm^−2^) was applied for 10 minutes during mixing before measurement by the microplate reader. To identify the primary contributor to ROS scavenging, nanosized Pd, TP (both at 200 µg mL^−1^), and NIR irradiation alone (1 W cm^−2^, 10 min) were tested for their ROS scavenging capacities using the same ROS testing kits and procedures.

### Cell Culture

RAW264.7 were commercially obtained from the American Type Culture Collection (ATCC, USA). RAW264.7 cells were cultured in DMEM supplemented with 10% FBS and 1% penicillin/streptomycin (Procell, China). The cultured medium was replaced with fresh medium every two days. Upon reaching 85% confluence, the cells were passaged, and the third passage was used for subsequent experiments.

### Cell Biocompatibility

Cell biocompatibility was initially evaluated using a CCK‐8 (Biosharp, China) following the protocols of manufacturer. Briefly, RAW264.7 was seeded into a 96‐well plate (5000 cells per well). The culture medium was removed, and replaced with 100 µL fresh solutions containing TP or TP@Pd at various concentrations (0, 5, 10, 20, 50, 100, 200, and 500 µg mL^−1^). After 24 h of incubation, cells were washed three times with PBS buffer, and then incubated with 10% CCK‐8 solution (100 µL) for another 2 h. Finally, the OD of the supernatant was measured at 450 nm using a spectrophotometer (Thermo Fisher, USA).

Additionally, the live/dead staining was performed to evaluate the protective ability of NPs for LPS stimulated RAW264.7. Briefly, cells were initially stimulated with LPS (1 µg mL^−1^) for 30 min, and then incubated with 200 µg mL^−1^ NPs for an additional 24 h. Specifically, for TP@Pd+NIR, NIR irradiation was applied three times (10 min per session, 1 W cm^−2^) at 0, 1, and 2 h after incubation. The experimental groups were as follows: cells without treatment (normal group), cells induced by LPS followed by PBS treatment (control group), cells induced by LPS followed by 200 µg mL^−1^ TP (TP) treatment, cells induced by LPS followed by 200 µg mL^−1^ TP@Pd (TP@Pd) treatment, and cells induced by LPS followed by 200 µg mL^−1^ TP@Pd combined with NIR irradiation (TP@Pd+NIR) treatment. After treatments, the cells were incubated with calcein‐AM/propidium iodide (PI, Beyotime Biotechnology, China) for 10 min, and washed for three times with PBS. Finally, the cells were imaged using fluorescence microscopy (Olympus, Japan), and the corresponding images were collected, and analyzed using ImageJ.

Finally, the hemolysis test was conducted to assess the hemocompatibility of NPs. After collecting the fresh blood from SD rats, the blood was centrifuged at 3000 rpm for 10 min, and erythrocyte pellet was collected after removing the supernatant. Subsequently, the pellet was re‐suspended in PBS buffer, and 0.5 mL of erythrocyte suspension was mixed with 0.5 mL of TP or TP@Pd at various concentrations (0, 5, 10, 20, 50, 100, 200, and 500 µg mL^−1^). And DI water (0.5 mL) served as the positive control. After 1 h of incubation, the mixture was centrifuged at 3000 rpm for 10 min, and the corresponding supernatant was measured at 540 nm using a microplate reader. Finally, the hemolysis ratio (HR) was calculated using the following formula HR = [(OD_s_‐OD_n_)/(OD_p_‐OD_n_)]/100, where OD_n_, OD_p_, and OD_s_ were the OD of negative control (0 µg mL^−1^ NPs), positive control and samples respectively.

### Cellular Uptake Investigation

At first, Initially, Cy5 labeled NPs were prepared by mixing 30 mg of TP or TP@Pd with 3 mg of Cy5‐PEG2000‐Thiol (Lumiprobe, China) in 50 mL of ethanol for 24 h. After centrifuge at 12 000 rpm for 30 min, the supernatant was removed, and the sample was re‐dispersed in ethanol. This procedure was repeated three times, and the final products (Cy5‐TP and Cy5‐TP@Pd) were collected by vacuum drying. For the cellular uptake experiment, RAW264.7 were cultured in confocal dishes, and incubated with Cy5‐TP or Cy5‐TP@Pd for 3 h. The treated cells were then washed three times with PBS, and fixed with 4% paraformaldehyde (PFA, Biosharp, China). Simultaneously, the cytoskeleton and nuclei of the cells were stained with actin‐tracker green‐488 (Actin, Biosharp, China) and DAPI (Biosharp, China) respectively following the manufacturer's instructions. Finally, the cells were washed three times with PBS, embedded in paraffin, and imaged using a confocal scanning microscopy (ZEISS, Germany). Specifically, to investigate the co‐localization of TP@Pd and lysosomes, Cy5‐TP@Pd treated cells were stained with a lysosome red fluorescent probe (Lyso‐Tracker Red, Solarbio, Beijing) before nuclei staining with DAPI. The final images were quantified, and fluorescence co‐localization was analyzed using ImageJ software.

### In Vitro Antioxidant and Anti‐Inflammatory Investigation

The intracellular ROS levels of treated RAW264.7 cells were assessed using ROS testing kits following the manufacturer's instructions. Specifically, RAW264.7 cells were stimulated with LPS (1 µg mL^−1^) for 30 min, and subsequently incubated with 200 µg mL^−1^ NPs for 24 h or subjected to NIR irradiation (1 W cm^−2^, 10 min) alone. For NIR irradiation, it was applied three times at 0, 1 and 2 h within the 24‐hour incubation period. After replacing the medium with fresh medium containing DCFH‐DA, HPF or DHE for total ROS, ·OH or ·O₂^⁻^ level detection, the cells were cultured for 30 min, and rinsed three times with PBS. After fixation with 4% paraformaldehyde (PFA), the cells were observed using a fluorescence microscope (ZEISS, Germany). In addition, the treated cells were washed three times with PBS, re‐suspended in PBS, and analyzed using flow cytometry. The supernatant from the treated cells was collected, and incubated with DCFH‐DA, HPF, and DHE probes respectively before analysis with a microplate reader to assess extracellular ROS levels.

Next, the expression levels of IL‐6, TNF‐α and IL‐1β were initially analyzed using ELISA. The supernatant from treated cells was collected and tested with ELISA kits (Meimian, China) according to the manufacturer's instructions. Additionally, the relative expression levels of these factors were further analyzed by immunofluorescent staining. The treated cells were fixed with PFA for 30 min, and blocked with goat serum. The cells were then incubated overnight at 4 °C with primary antibodies against IL‐6, iNOS, CD206, HSP70, or CD31 (1:200 dilutions, Proteintech, China). After washing three times with PBS, the cells were incubated with a secondary antibody (FITC‐conjugated AffiniPure Goat Anti‐Rabbit IgG, Boster, China) for 2 h, and stained with DAPI for 10 min. After additional PBS washing, the cells were imaged using fluorescence microscopy and analyzed with ImageJ. Finally, the expression levels of inflammatory genes (IL‐6 and IL‐1β), antioxidant gene (SOD2), M1 type genes (iNOS and CD86), M2 type genes (CD206 and IL‐10), and tissue repair genes (HSP70 and CD31) were analyzed using quantitative real‐time PCR (qRT‐PCR). At first, the total RNA was extracted from treated cells using an RNA extraction kit (Magen, China), and qRT‐PCR was performed by using the LightCycler® System (Roche, Switzerland). And the relative genes levels were analyzed by 2^−ΔΔCt^ method, and compared with glyceraldehyde‐3‐phosphate dehydrogenase (GAPDH). The detailed primer sequences were listed in Table S9 (Supporting Information).

### Relative Mechanism Analysis

To investigate the collection of all intracellular transcripts under specific physiological conditions, RNA sequencing was performed. Briefly, cells were cultured in a 6‐well plate, and induced with LPS (1 µg mL^−1^) upon reaching 85% confluence. Subsequently, the cells were treated with PBS or 200 µg mL^−1^ TP@Pd+NIR for 24 h. The total RNA of treated cells was extracted using an RNA extraction kit (Magen, China), and genomic DNA was digested with DNase I (Takara, Japan). The transcriptome library was then constructed, and used for high throughput RNA sequencing. After sequencing, the corresponding sequencing analysis was conducted using R software (version 4.42) to identify the DEGs between control group and TP@Pd+NIR. Statistical significance was determined with the *p*‐value < 0.05 and a Log₂ fold change >1.

To further confirm the anti‐inflammatory mechanism, WB was performed to detect the relative protein expression levels of specific target genes. Briefly, the treated cells were washed with ice‐cold PBS, and lysed using lysis buffer containing protease inhibitors and phosphatase inhibitors. After centrifuge at 12 000 rpm for 15 min, the supernatant was collected, and the protein concentration was measured using a BCA protein assay kit (Beyotime, China). Later, the proteins were separated by gel electrophoresis, and transferred onto polyvinylidene fluoride (PVDF) membranes (IPVH00010, Millipore, USA). And the membranes were blocked with blocking buffer for 2 h, and rinsed three times with Tris‐buffered saline with Tween‐20 (TBST, Sigma, USA). They were then incubated overnight at 4 °C with primary antibodies against IκBα, p‐IκBα, p65, or p‐p65 (Proteintech, China). After washing three times with TBST, the membranes were incubated with the secondary antibody (Goat Anti‐Rabbit, Sangon, China) for 2 h. At last, the membranes were soaked in 1 mL of UltraSignal ECL western blotting detection reagent (Beyotime, China), and scanned using an automatic chemiluminescence image analysis system (Bio‐Rad, USA).

### In Vivo Biodistribution and Photothermal Effect

The in vivo experiment was conducted with the approval of the Ethics Committee for Animal Experiments of Guangxi Medical University. SD rats (180 g) were selected, and treated in accordance with the local guidelines of the care and use of laboratory animals at Guangxi Medical University. To evaluate in vivo biodistribution, IVIS were used after IV injection of NPs. Briefly, after anesthesia, SD rats were IV injected with 0.8 mL of sample solutions (200 µg mL^−1^ TP@Pd, Cy5, or Cy5‐TP@Pd). The major organs, including heart, liver, spleen, lung, and kidney, were collected at selected time points (0, 0.5, 1, 2, 6, and 24 h), and imaged using IVIS with excitation and emission wavelengths of 646 nm and 664 nm respectively. The in vivo photothermal effects were evaluated as follows: 0.8 mL of TP@Pd (200 µg mL^−1^) was IV injected into SD rats. After 30 min, the lungs of the rats were irradiated with NIR light (1 W cm^−2^) for 15 min. The corresponding photothermal images and temperatures were collected and recorded at predetermined time points using a thermal camera.

### In Vivo Biosafety Evaluation

To evaluate in vivo biosafety, SD rats (*n* = 12, 6 in each group) were intravenously injected with 0.8 mL of saline or TP@Pd (200 µg mL^−1^) respectively. The body weight of each rat was measured daily for 7 days. After 7 days, the blood samples from rats were analyzed for routine blood testing using a fully automatic blood analyzer (BC‐6800Plus, Mindray, China), biochemical testing with a fully automatic biochemical analyzer (7600, HITACHI, Japan), and coagulation function testing using a fully automatic coagulation detection analyzer (ACL TOP750, Werfen, USA). Significantly, the heart, liver, spleen, lung, and kidney were isolated and collected. After embedding, the organs were sectioned and stained with H&E for observation under an Olympus microscopy.

### In Vivo Therapy Evaluation

To establish ALI animal models, SD rats were anesthetized, and a midline incision was made along the neck to expose the trachea of rats. LPS (1 mg mL^−1^, 5 mg kg^−1^) was infused into the trachea, after which the incision was sutured for further experiments. The SD rats (*n* = 36, 9 per group) were randomly divided into four groups: rats without treatment (sham group), ALI rats with IV injection of saline (ALI group), ALI rats with IV injection of 0.8 mL of TP@Pd (200 µg mL^−1^, TP@Pd), and ALI rats with IV injection of 0.8 mL of TP@Pd (200 µg mL^−1^) combined with NIR irradiation (TP@Pd+NIR). Specifically, NIR irradiation was applied for three times (1 W cm^−2^, 10 min), respectively at 1, 2, and 3 h after IV injection in TP@Pd+NIR. During the establishment of ALI models, LPS intervention was performed for 30 min before treatment. After 24 h of treatment, the blood samples and major organs from rats were collected, and stored for further analysis. The blood samples were analyzed for routine blood tests, biochemical parameters and coagulation function. Additionally, the fresh blood was centrifuged to collect serum, applied to assess the inflammatory factors expression levels by ELISA.

Additionally, after collecting the major organs from rats, the lungs were photographed, and their w/d weight ratios were calculated by weighing the lungs before and after drying. Similarly, the lungs were cut into pieces, homogenized, and centrifuged at 3000 rpm for 10 min. And the supernatant was collected to measure the TNF‐α, IL‐6 and IL‐1β expression levels using ELISA. Furthermore, the SOD activity of lung homogenates was analyzed using a total SOD activity detection kit. And the lipid peroxidation levels were also evaluated using a MDA detection kit (Solarbio, China) following the manufacturer's instructions.

Furthermore, the isolated lungs were fixed in 4% PFA for 24 h, and sectioned into 3 µm thick slices for subsequent experiments. First, the ROS levels in lung tissues were investigated using a DHE probe following the manufacturer's instructions, followed by nuclei stained by DAPI. After mounting, the slides were imaged using a fluorescence microscope. Meanwhile, lung sections were subjected to H&E staining, imaged using an Olympus microscope, and evaluated using Smith scoring. For immunohistochemical and immunofluorescent staining, the slides were incubated overnight at 4 °C with primary antibodies (rabbit polyclonal anti‐IL‐6, anti‐TNF‐α, anti‐iNOS, anti‐CD206, anti‐HSP70 or anti‐CD31, Servicebio, China), and washed three times against with PBS. Next, the slides were incubated with biotinylated or fluorescent labeled secondary antibodies. After rinsing and sealing, the slides were imaged using an Olympus microscope, and the AOD was analyzed using ImageJ software. Additionally, the macrophage polarization levels (iNOS and CD206 genes) in lung tissues were quantified by RT‐qPCR. After extracting the total RNA from lung tissues, the genes expression levels were analyzed. To further evaluate in vivo immunotherapy, the number of CD4^+^ and CD8^+^ T cells in blood and lung tissues of treated rats were quantified using flow cytometry. Fresh blood and lung tissues were collected, and the corresponding cells were isolated for analysis. Similarly, the expression levels of CD4^+^ and CD8^+^ T cells in lung tissues were analyzed by immunofluorescent staining after incubating the lung sections with primary antibodies (rabbit polyclonal anti‐CD4^+^ and anti‐CD8^+^, Servicebio, China).

At last, the heart, liver, spleen, and kidney were also isolated from rats, and imaged by the camera. And these organs were fixed by 4% PFA, and cut into sections with 3 µm thickness for H&E staining.

### Statistical Analysis

All experiments were conducted in triplicate unless otherwise specified, and the data are presented as mean ± standard deviation. And the statistical significance was determined using ANOVA followed by least significant difference (LSD) analysis.

## Conflict of Interest

The authors declare no conflict of interest.

## Supporting information



Supporting Information

## Data Availability

The data that support the findings of this study are available on request from the corresponding author. The data are not publicly available due to privacy or ethical restrictions.
