# Supplementary material for: Reprogramming Lung Redox Homeostasis by NIR Driven Ultra‐Small Pd Loaded Covalent Organic Framework Inhibits NF‐κB Pathway for Acute Lung Injury Immunotherapy
Source: Adv Sci (Weinh). 2025 Feb 18;12(14):2413697. doi: 10.1002/advs.202413697 (PMC11984858; doi:10.1002/advs.202413697)
Supplement: Supplementary file 1 — Supporting Information [file ADVS-12-2413697-s001.docx]

**Supplemental Material**

**Reprogramming lung redox homeostasis by NIR driven ultra-small Pd loaded covalent organic framework inhibits NF-κB pathway for acute lung injury immunotherapy**

Doudou Lei ^1 #^, Lin Liao ^2 #^, Tao Qin ^3 #^, Xiaoxuan Guan ^4^, Kunpeng Duan ^4^, Zhiwei Gao ^4^, Weiqian Jin ^4^, Mingjing Yin ^5^, Ke Zhang ^5^, Yan Liu ^5^, Yin Chen ^1^, Huyang Gao ^4^, Jiaxiao Li ^1^, Feifei Huang ^4^, Wenjing Liu ^6^, Chengde Xia ^7^, Bailei Wang ^8^, Hualin Huang ^1^, Shengqiu Lv ^1^, Qiang Zhi ^1^, Jiahao Huang ^5 *^, Ming Gao ^4 *^ and Junyu Lu ^1 *^

^1^ Intensive Care Unit, The Second Affiliated Hospital of Guangxi Medical University, Nanning, Guangxi 530007, China

^2^ Department of Clinical Laboratory, Key Laboratory of Clinical Laboratory Medicine of Guangxi Department of Education, The First Affiliated Hospital of Guangxi Medical University, Nanning, Guangxi 530007, China

^3^ Department of Emergency, Guangxi Medical University Cancer Hospital, Nanning, Guangxi 530021, China

^4^ Life Sciences Institute, Guangxi Medical University, Nanning, Guangxi 530021, China

^5^ Department of Colorectal and Anal Surgery, Department of Emergency, The First Affiliated Hospital of Guangxi Medical University, Nanning, Guangxi 530007, China

^6^ Plastic Surgery, The Second Affiliated Hospital of Nanchang University, Nanchang, Jiangxi 330006, China

^7^ Department of Burns, The First People’s Hospital of Zhengzhou, Zhengzhou 450004, China

^8^ Department of Critical Care Medicine, The Ninth Affiliated Hospital of Guangxi Medical University, Beihai 536000, China

^#^ These authors contributed equally.

**Fig. S1.** Optical image of TAPA, PA, TP and TP@Pd.


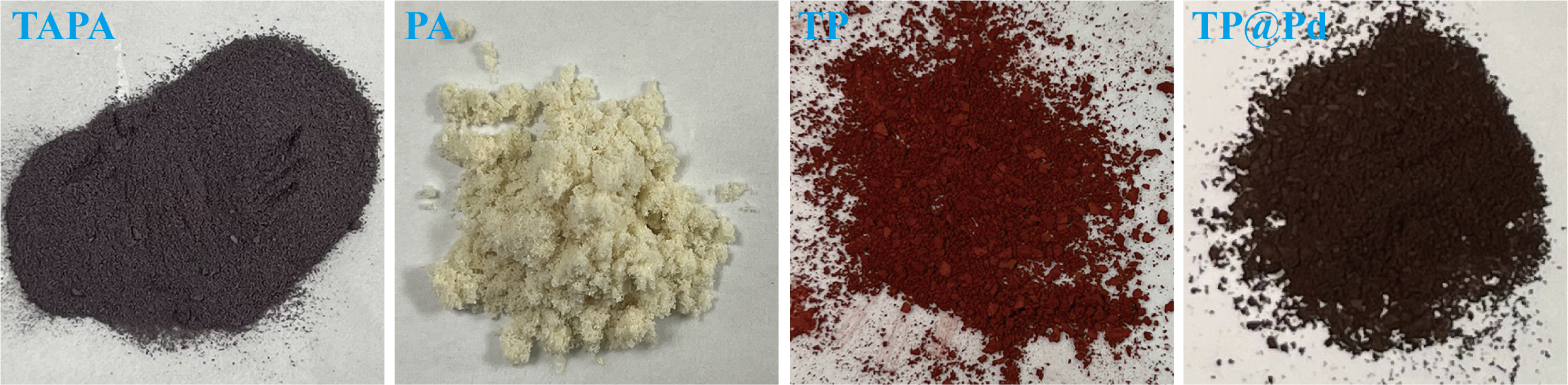

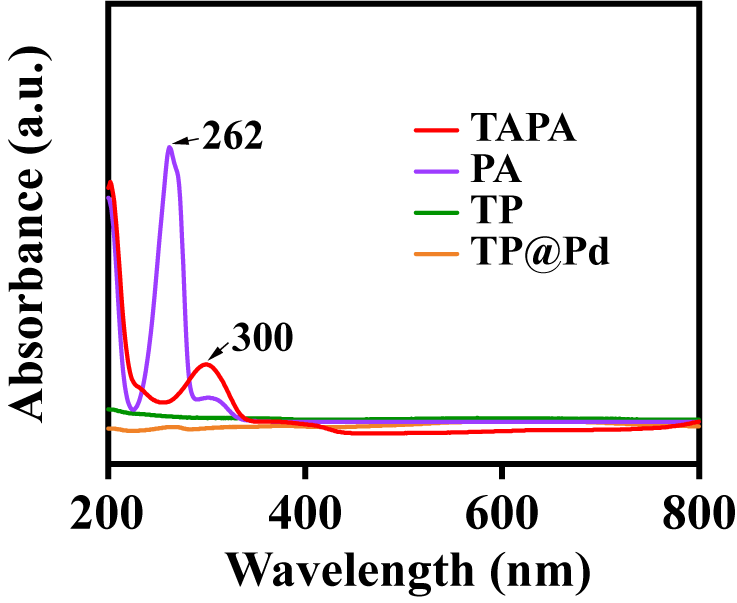


**Fig. S2.** UV-vis results of TAPA, PA, TP and TP@Pd.


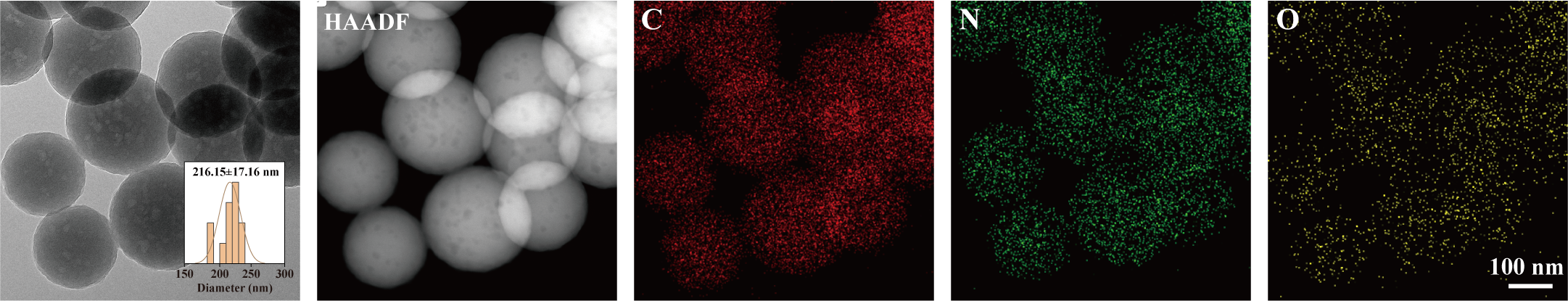


**Fig. S3.** TEM-mapping images of TP and the corresponding element composition (HAADF, C, N and O).


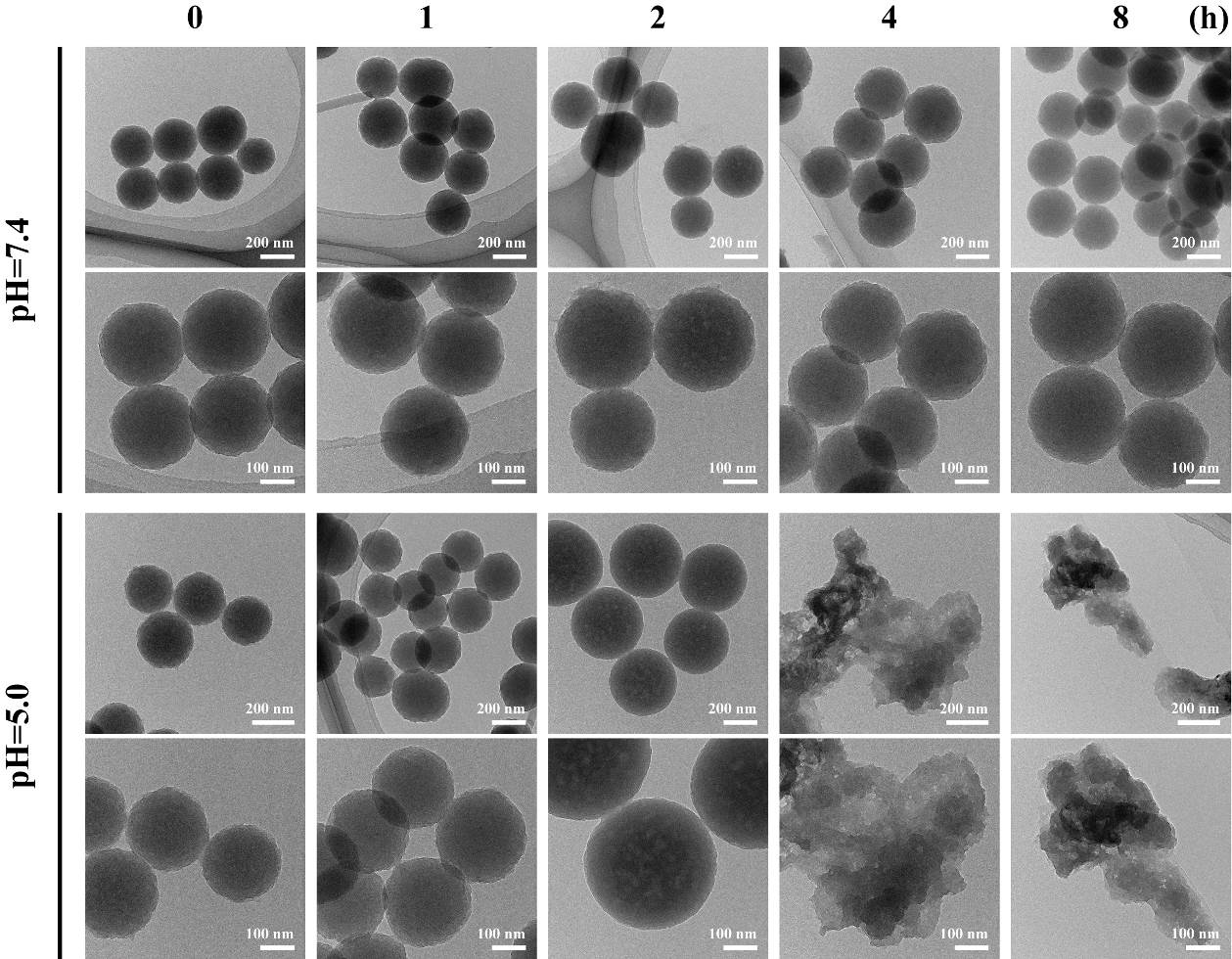


**Fig. S4.** Morphology changes of TP@Pd in PBS and pH=5.0 versus time by TEM.


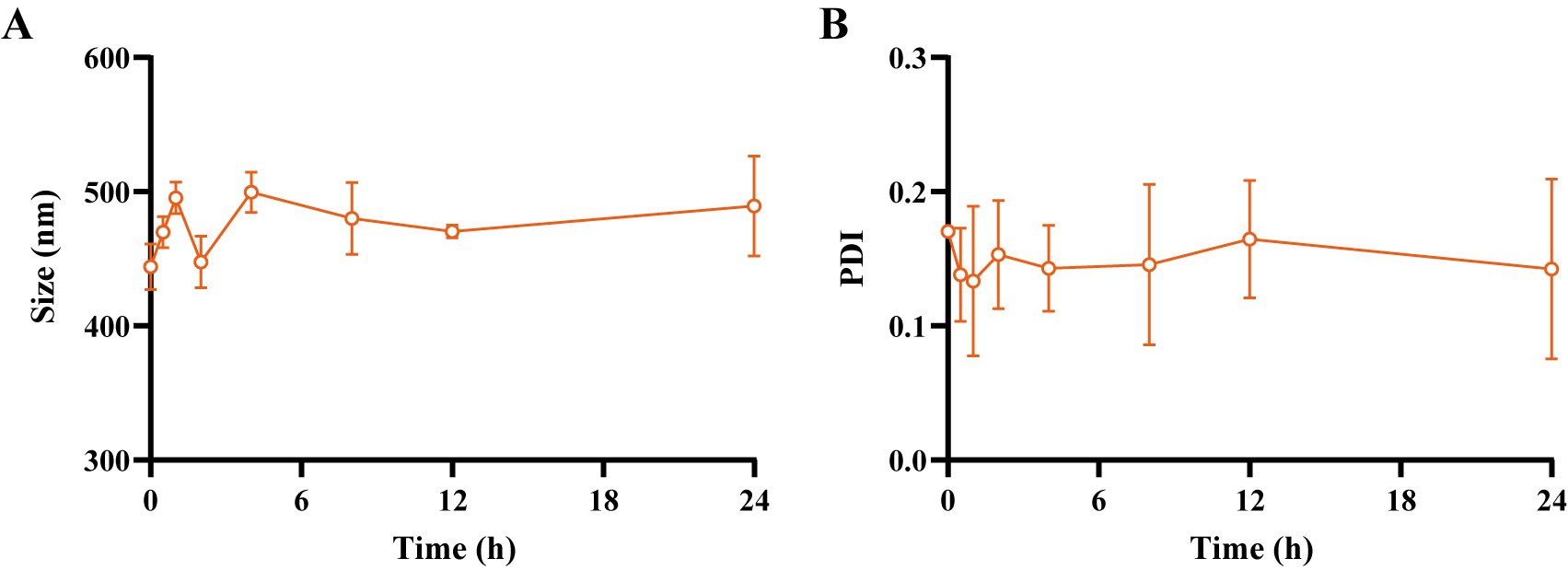


**Fig. S5.** Size and PDI changes of TP@Pd in PBS versus time by zeta sizer.


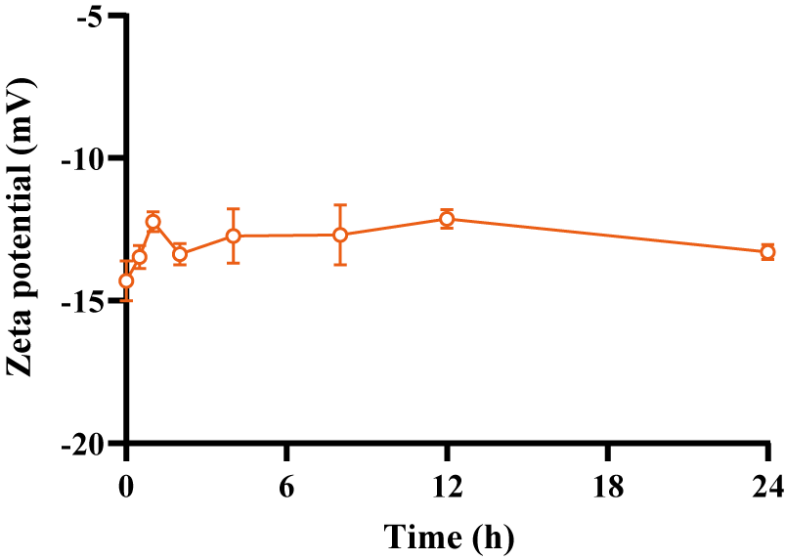


**Fig. S6.** Zeta potential changes of TP@Pd in PBS versus time by zeta sizer.


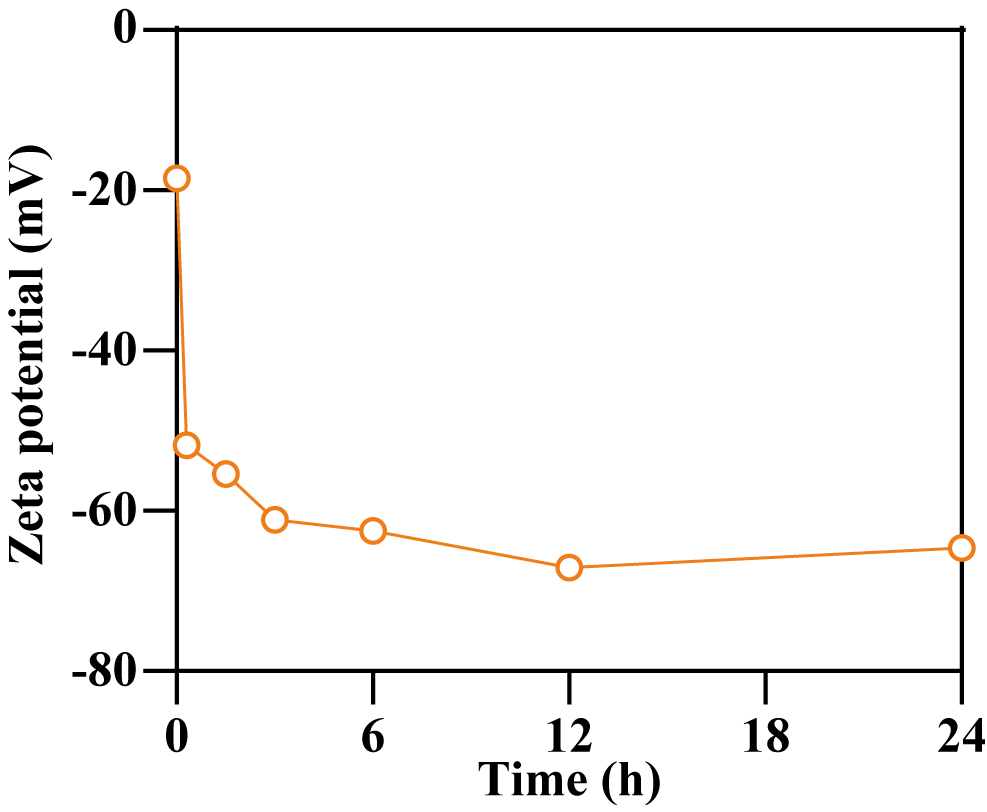


**Fig. S7.** Zeta potential changes of TP@Pd in pH=5.0 versus time by zeta sizer.


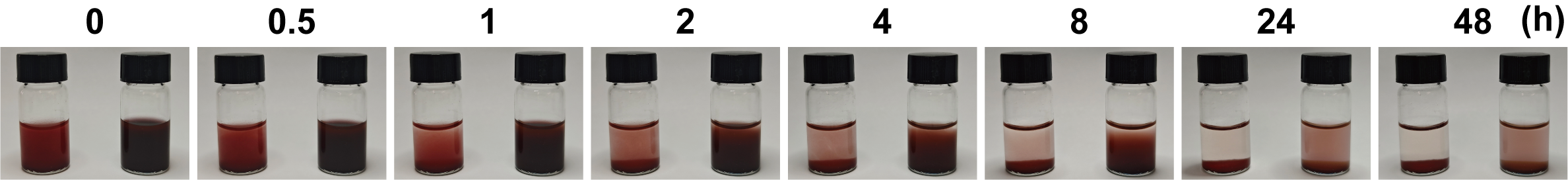


**Fig. S8.** Dispersion and stability of TP (left) and TP@Pd (right) in blood serum of SD rats at predetermined time points (0, 0.5, 1, 2, 4, 8, 24 and 48 h).


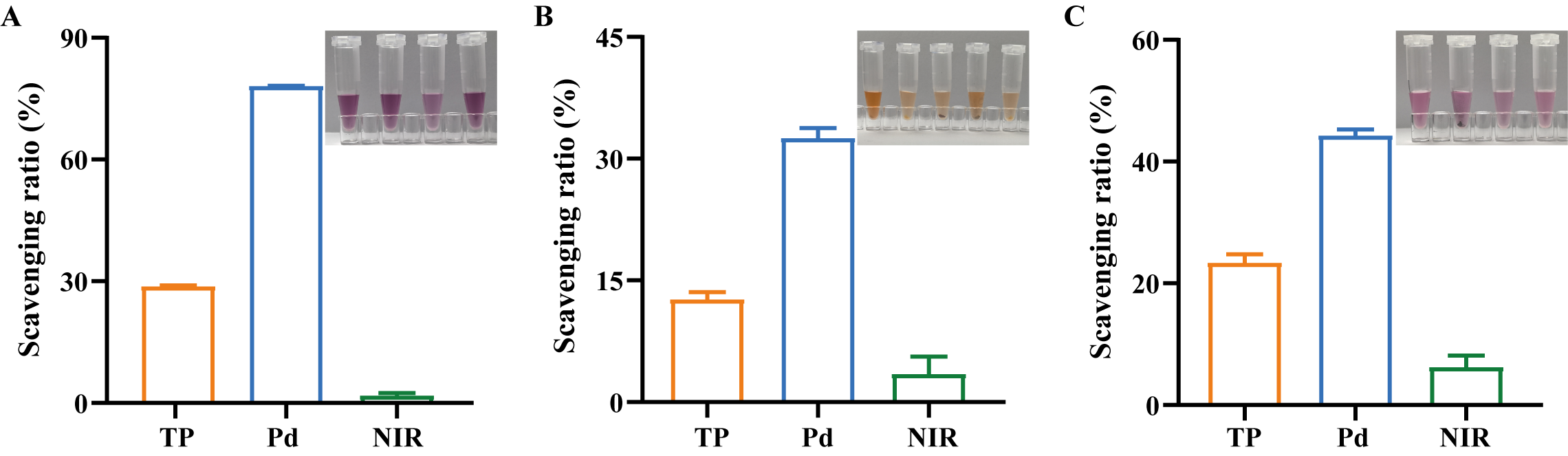


**Fig. S9.** ROS scavenging capacity by CAT (A), ·OH (B) and ·O_2_^-^ (C) testing kits: nanosized Pd and TP with 200 μg/mL and NIR irradiation alone (NIR) (808 nm, 1 W/cm^2^, 10 min).


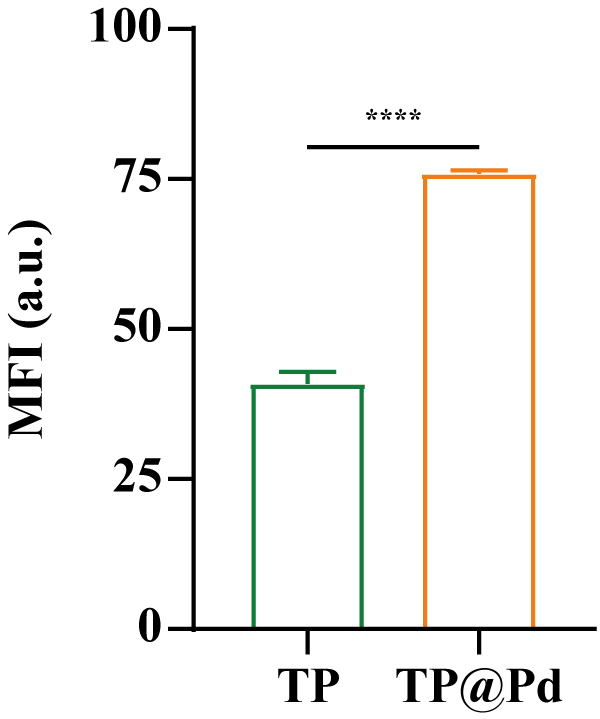


**Fig. S10.** Quantified results of cellular uptake by confocal microscopy. The corresponding groups were: cells respectively incubated with Cy5-TP (TP) or Cy5-TP@Pd (TP@Pd) for 3 h. (“*” symbol compared between TP and TP@Pd, *p<0.05, **p<0.01, ***p<0.001 and ****p<0.0001)


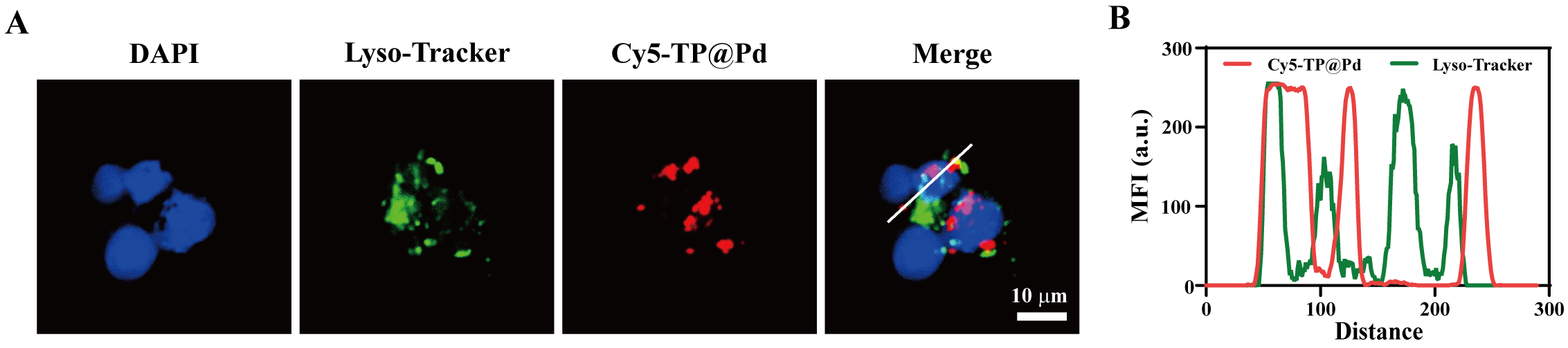


**Fig. S11.** Co-staining images of lysosome and TP@Pd in macrophage by confocal microscopy, and the corresponding co-localization results (B): nucleus (DAPI), lysosome (Lyso-Tracker) and TP@Pd (Cy5-TP@Pd).


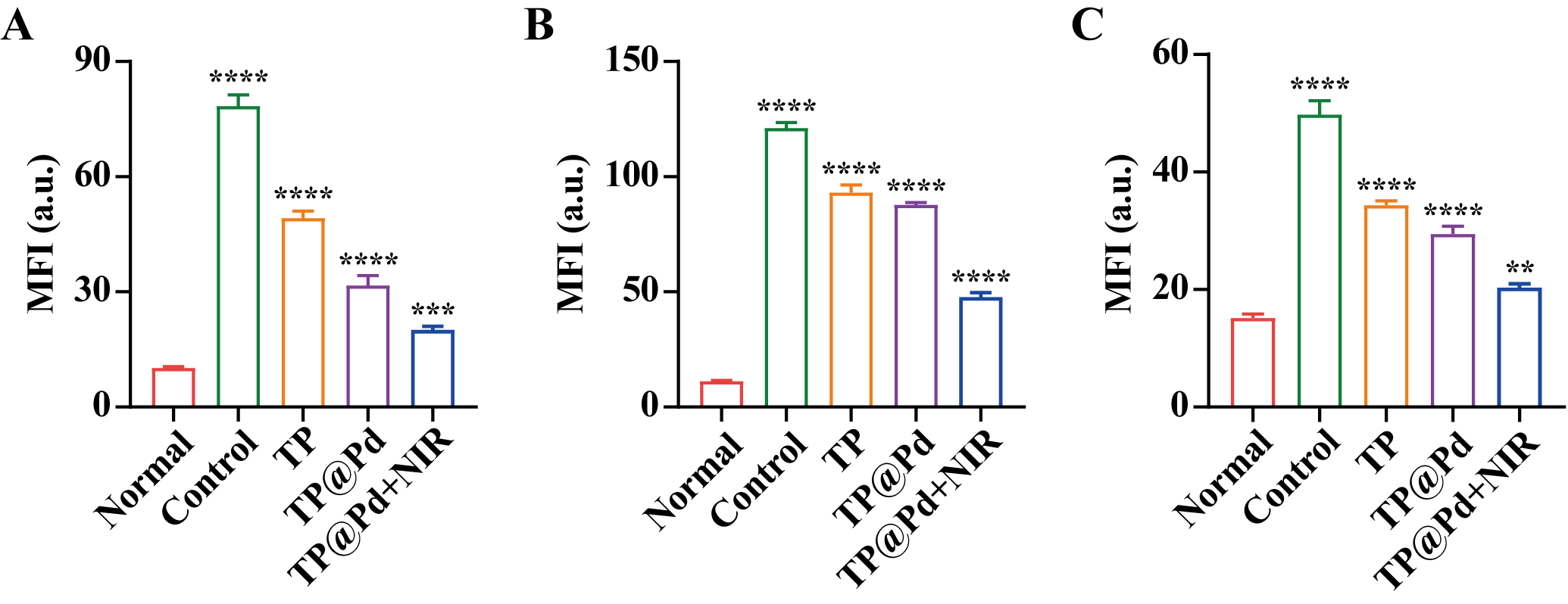


**Fig. S12.** Quantified results of intracellular ROS levels of treated cells: DCFH-DA (A), DHE (B) and HPF (C). The corresponding groups were: cells without treatment (normal group), cells pre-treated with LPS followed by incubating with PBS (control group), cells pre-treated with LPS followed by incubating with 200 μg/mL TP (TP), cells pre-treated with LPS followed by incubating with 200 μg/mL TP@Pd (TP@Pd), and cells pre-treated with LPS followed by incubating with 200 μg/mL TP@Pd and NIR irradiation (1 W/cm^2^) (TP@Pd+NIR). (“*” symbol compared with normal group, *p<0.05, **p<0.01, ***p<0.001 and ****p<0.0001)


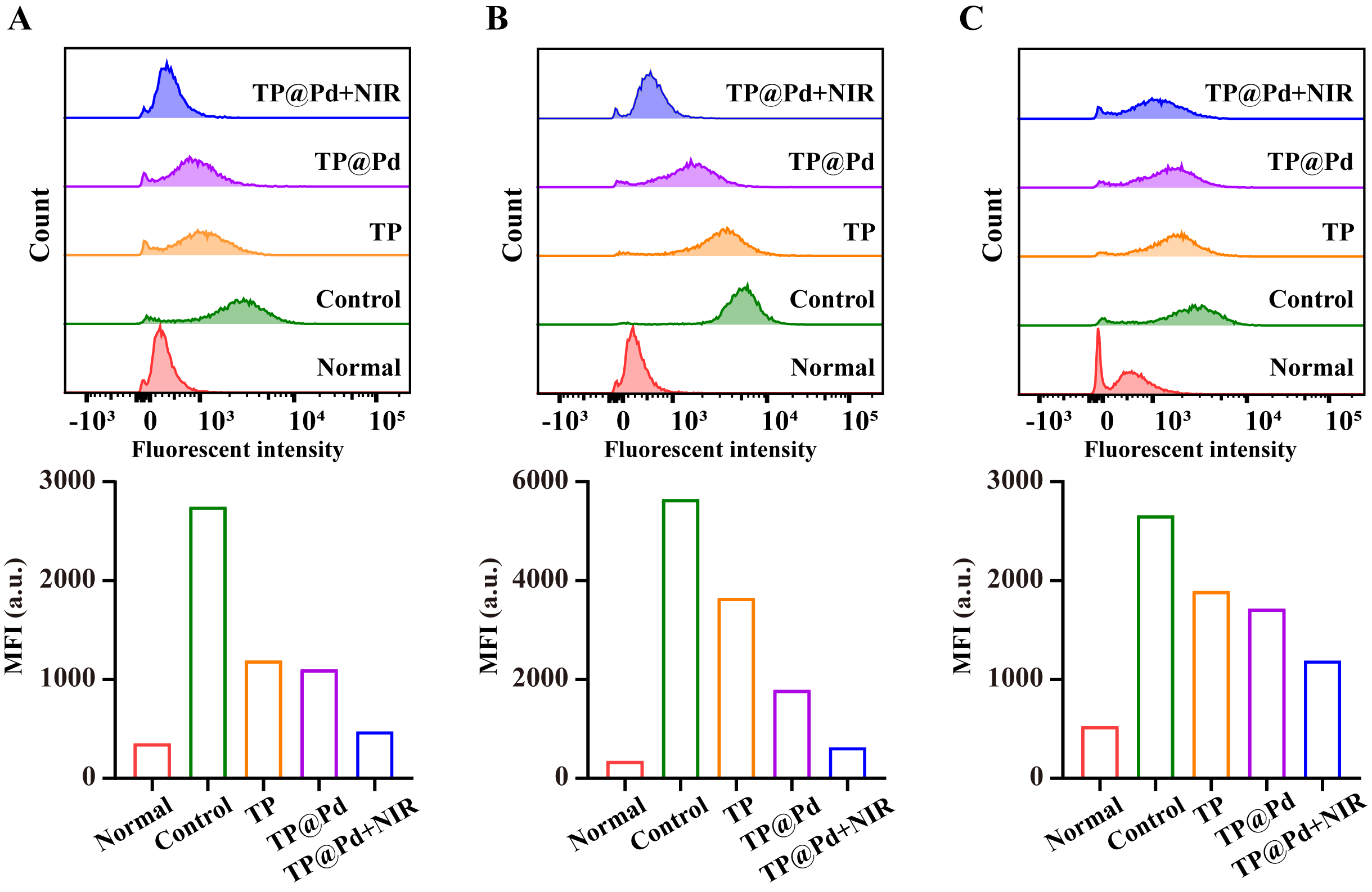


**Fig. S13.** The intracellular ROS levels of treated cells by flow cytometry and the corresponding quantified results: DCFH-DA (A), DHE (B) and HPF (C). The corresponding groups were: cells without treatment (normal group), cells pre-treated with LPS followed by incubating with PBS (control group), cells pre-treated with LPS followed by incubating with 200 μg/mL TP (TP), cells pre-treated with LPS followed by incubating with 200 μg/mL TP@Pd (TP@Pd), and cells pre-treated with LPS followed by incubating with 200 μg/mL TP@Pd and NIR irradiation (1 W/cm^2^) (TP@Pd+NIR).


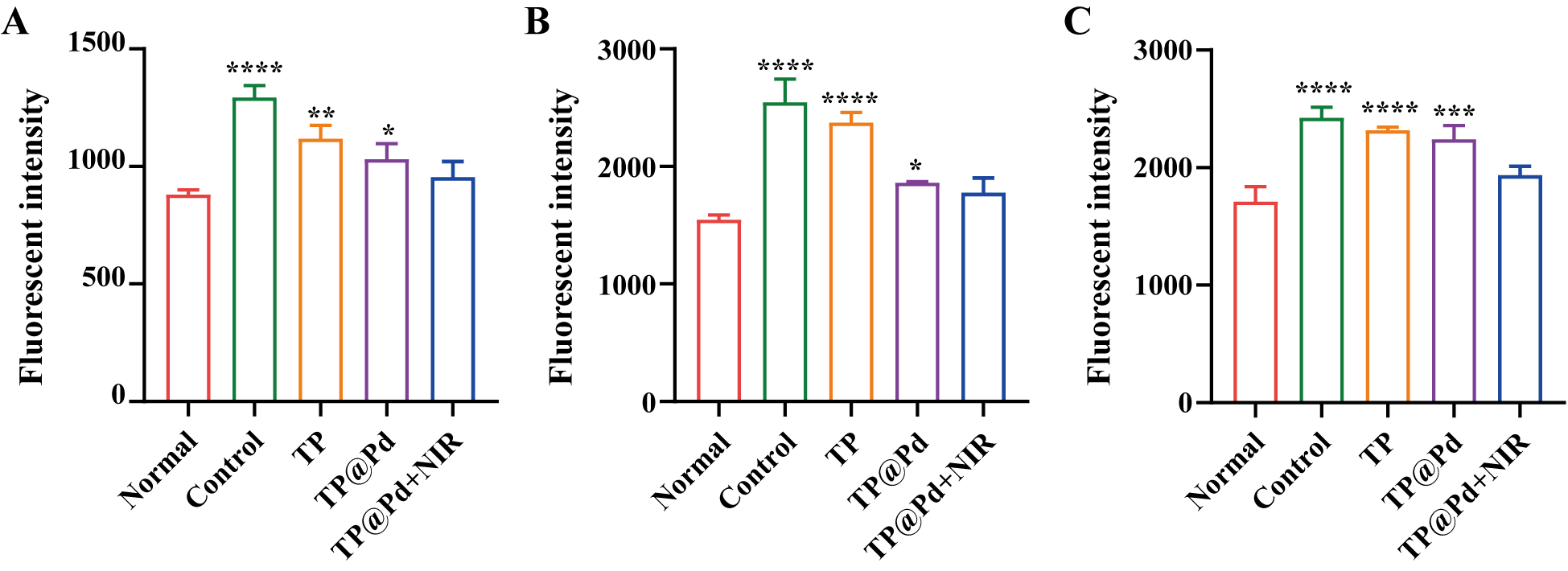


**Fig. S14.** The extracellular ROS levels of treated cells by ROS testing probes: DCFH-DA (A), DHE (B) and HPF (C). The corresponding groups were: cells without treatment (normal group), cells pre-treated with LPS followed by incubating with PBS (control group), cells pre-treated with LPS followed by incubating with 200 μg/mL TP (TP), cells pre-treated with LPS followed by incubating with 200 μg/mL TP@Pd (TP@Pd), and cells pre-treated with LPS followed by incubating with 200 μg/mL TP@Pd and NIR irradiation (1 W/cm^2^) (TP@Pd+NIR). (“*” symbol compared with normal group, *p<0.05, **p<0.01, ***p<0.001 and ****p<0.0001)


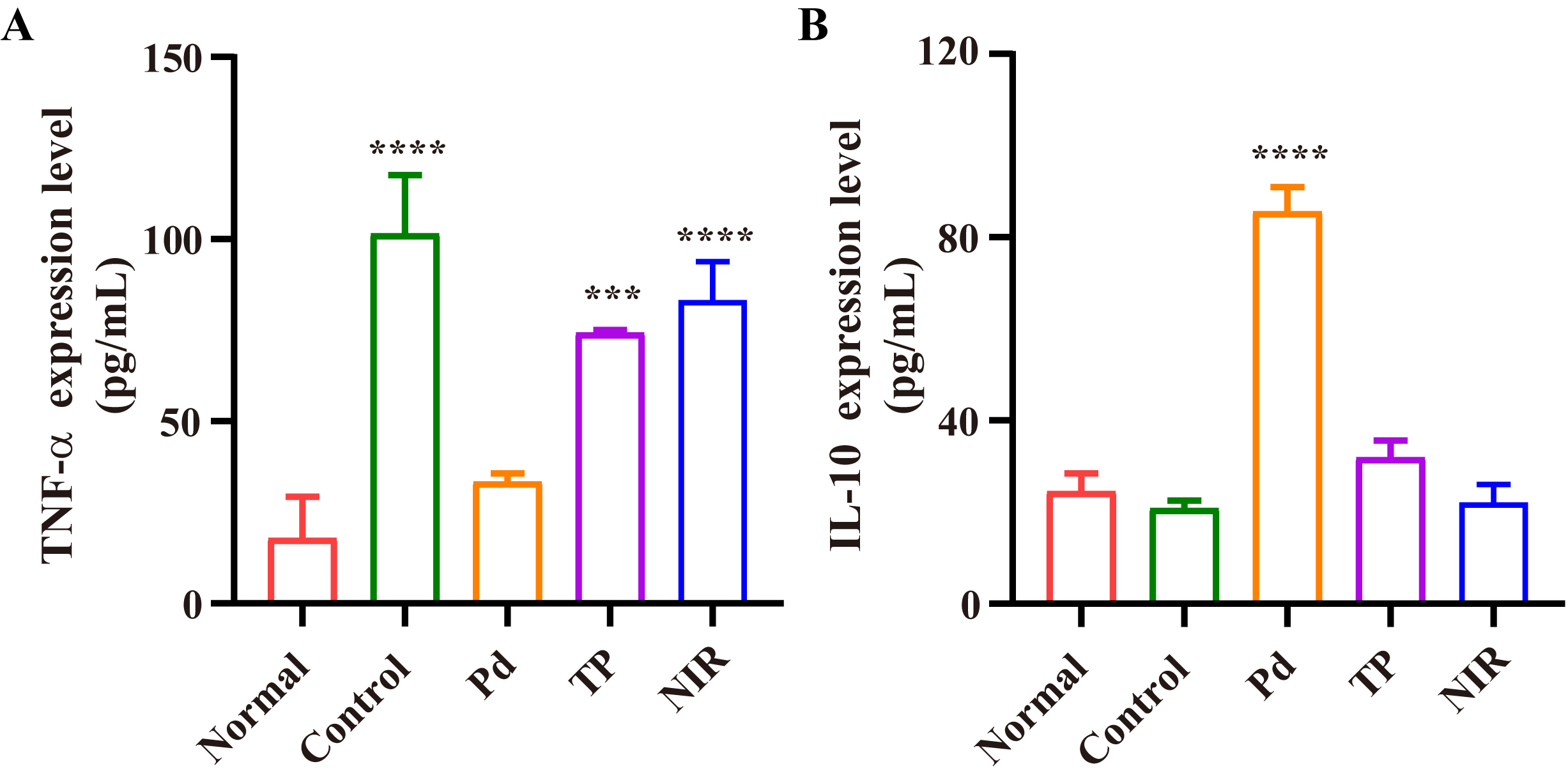


**Fig. S15.** TNF-α (A) and IL-10 (B) expression levels of the supernatant of treated cells by ELISA. The corresponding groups were: cells without treatment (normal group), cells pre-treated with LPS followed by incubating with PBS (control group), cells pre-treated with LPS followed by incubating with 200 μg/mL Pd (Pd), cells pre-treated with LPS followed by incubating with 200 μg/mL TP (TP), and cells pre-treated with LPS followed by NIR irradiation alone (1 W/cm^2^) (NIR). (“*” symbol compared with normal group, *p<0.05, **p<0.01, ***p<0.001 and ****p<0.0001)

**
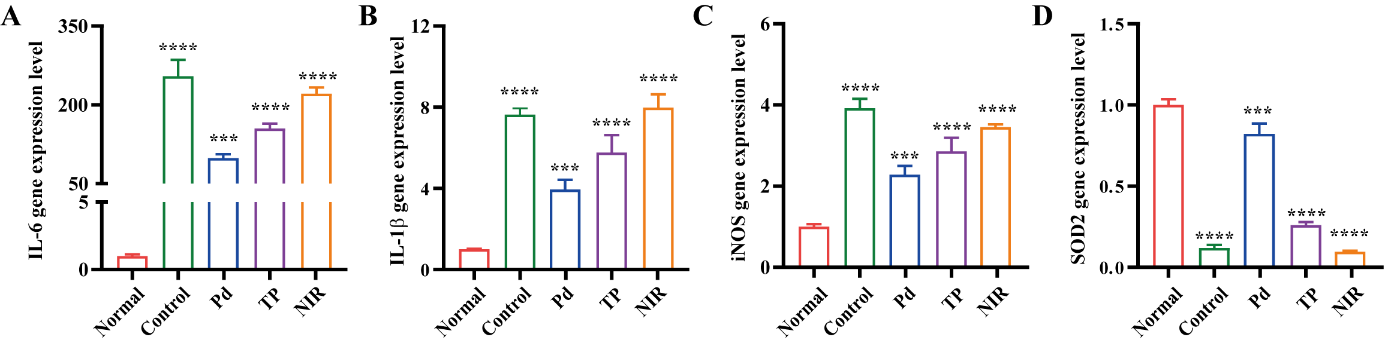
**

**Fig. S16.** Relative genes expression levels of treated cells by RT-qPCR: IL-6 (A), IL-1β (B), iNOS (C) and SOD2 (D). The corresponding groups were: cells without treatment (normal group), cells pre-treated with LPS followed by incubating with PBS (control group), cells pre-treated with LPS followed by incubating with 200 μg/mL Pd (Pd), cells pre-treated with LPS followed by incubating with 200 μg/mL TP (TP), and cells pre-treated with LPS followed by NIR irradiation alone (1 W/cm^2^) (NIR). (“*” symbol compared with normal group, *p<0.05, **p<0.01, ***p<0.001 and ****p<0.0001)

**
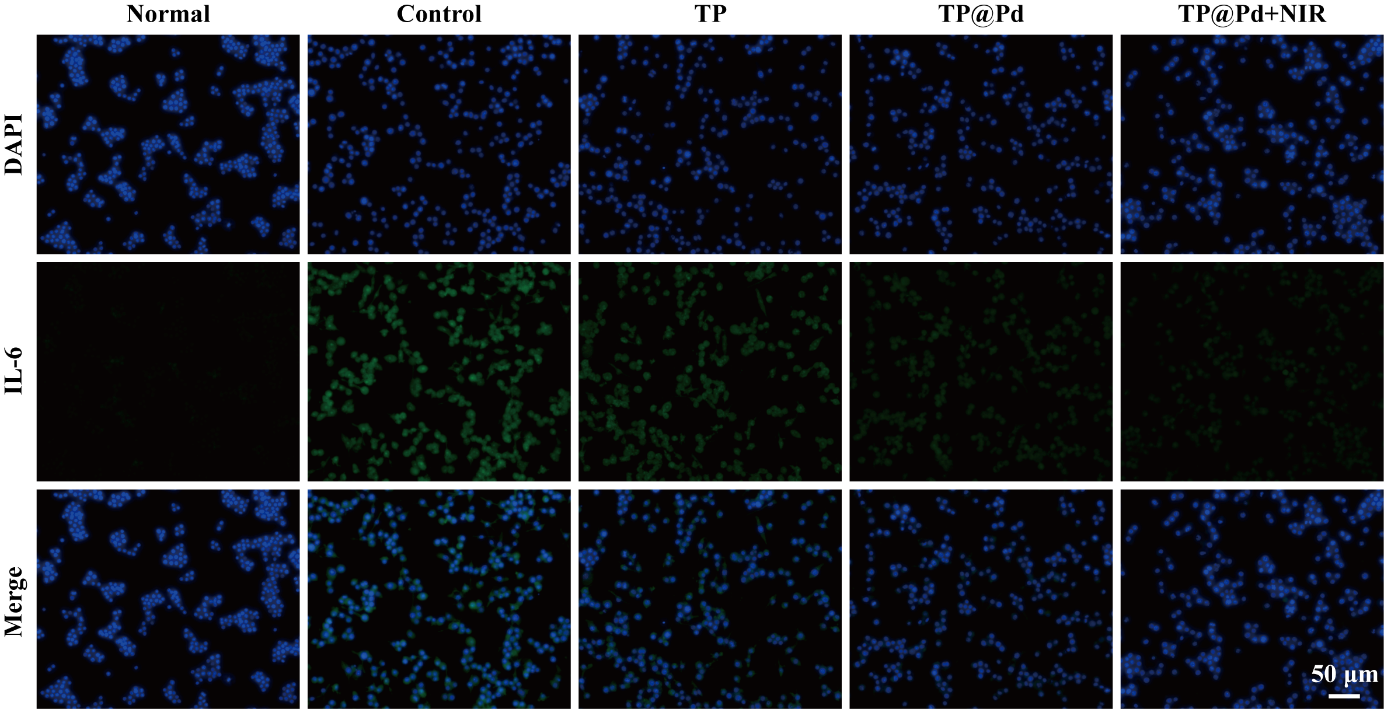
**

**Fig. S17.** IL-6 expression level of treated cells by fluorescent microscopy. The corresponding groups were: cells without treatment (normal group), cells pre-treated with LPS followed by incubating with PBS (control group), cells pre-treated with LPS followed by incubating with 200 μg/mL TP (TP), cells pre-treated with LPS followed by incubating with 200 μg/mL TP@Pd (TP@Pd), and cells pre-treated with LPS followed by incubating with 200 μg/mL TP@Pd and NIR irradiation (1 W/cm^2^) (TP@Pd+NIR).

**
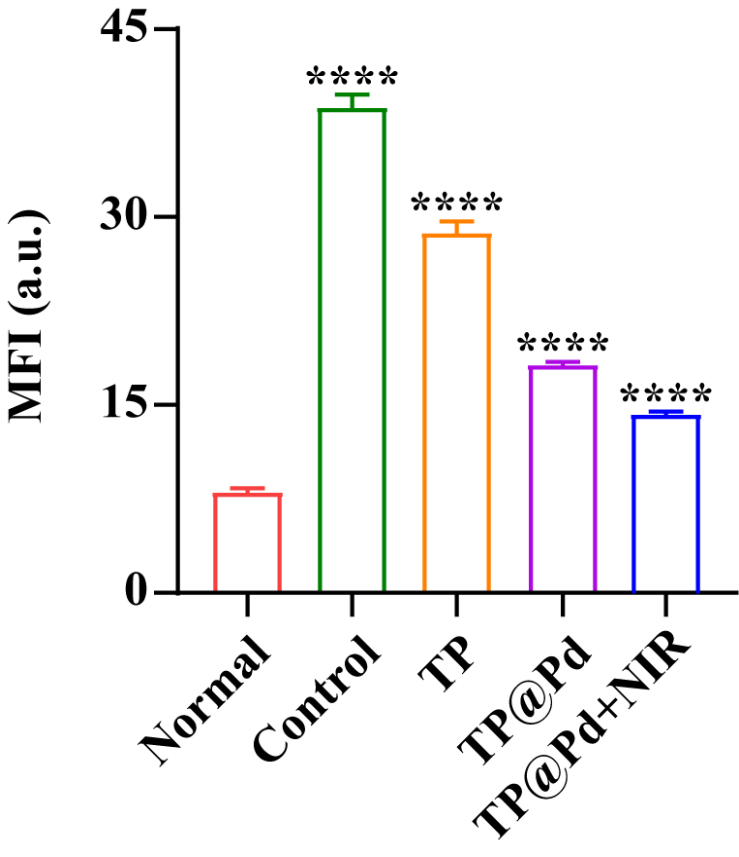
**

**Fig. S18.** Quantified results of IL-6 expression level of treated cells by fluorescent microscopy. The corresponding groups were: cells without treatment (normal group), cells pre-treated with LPS followed by incubating with PBS (control group), cells pre-treated with LPS followed by incubating with 200 μg/mL TP (TP), cells pre-treated with LPS followed by incubating with 200 μg/mL TP@Pd (TP@Pd), and cells pre-treated with LPS followed by incubating with 200 μg/mL TP@Pd and NIR irradiation (1 W/cm^2^) (TP@Pd+NIR). (“*” symbol compared with normal group, *p<0.05, **p<0.01, ***p<0.001 and ****p<0.0001)

**
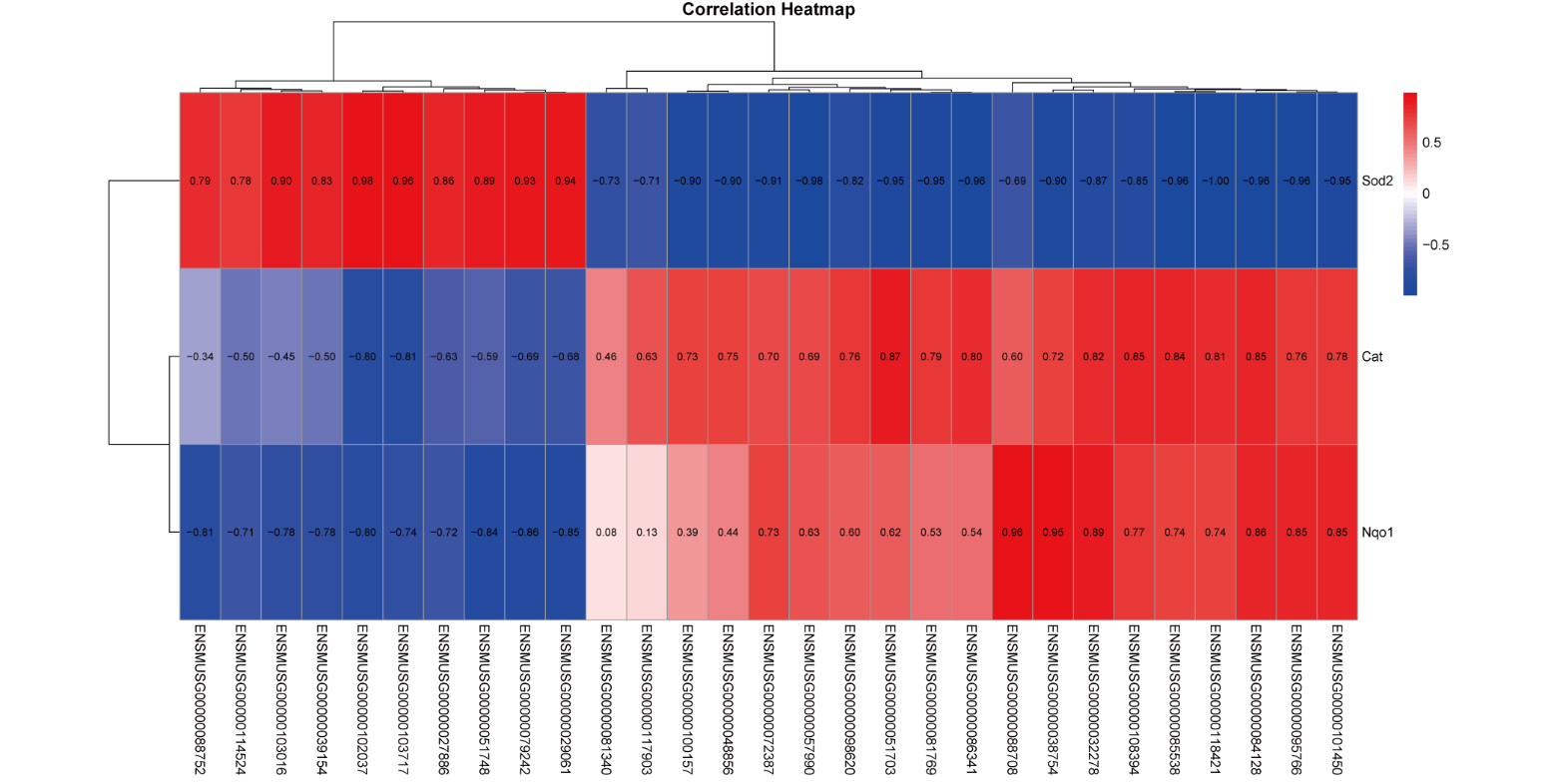
Fig. S19**. A correlation heatmap of typical antioxidant enzymes and DEGs.


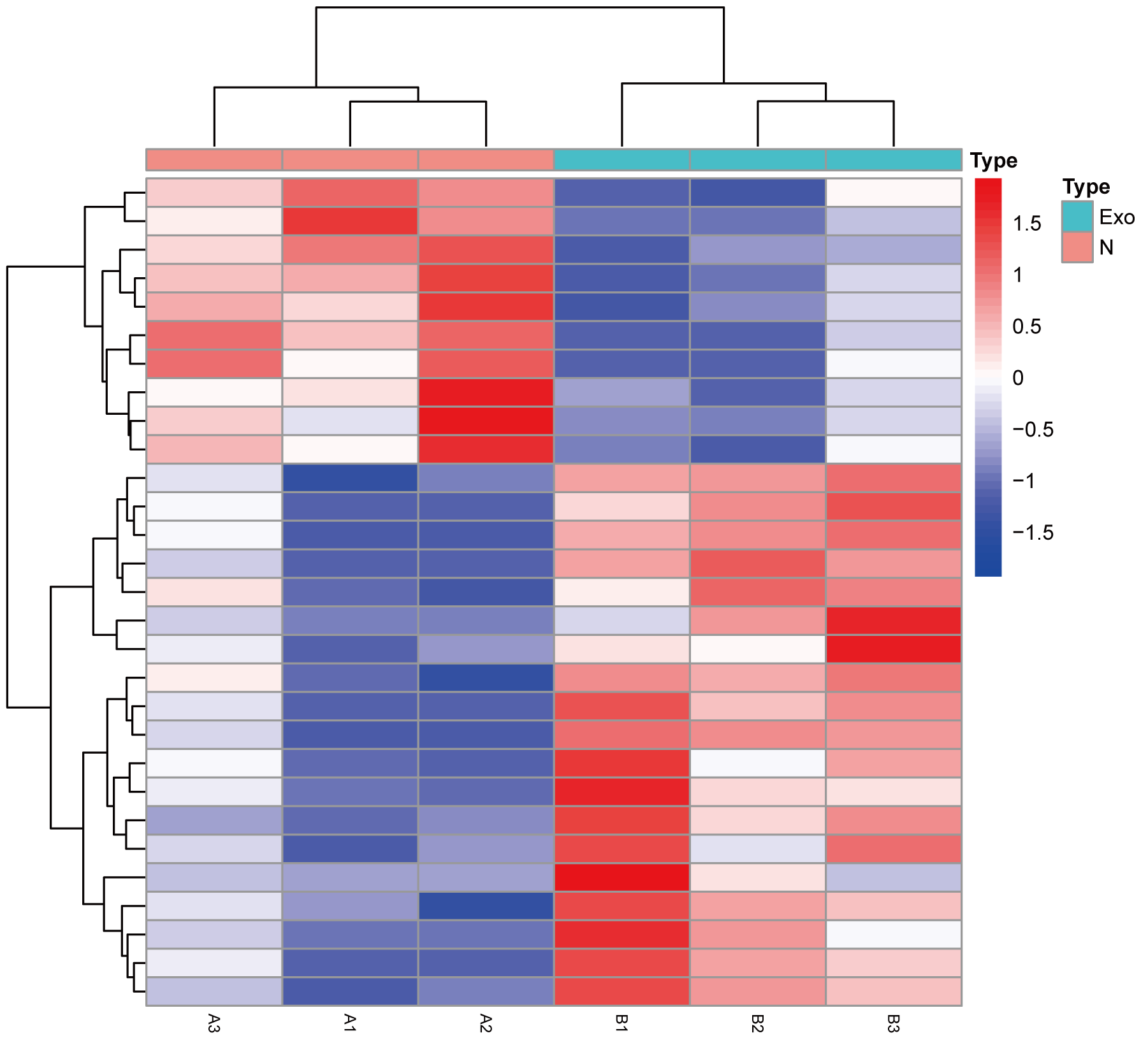


**Fig. S20**. A heatmap of antioxidant related DEGs between control group (A1, A2 and A3) and TP@Pd (B1, B2 and B3).

**
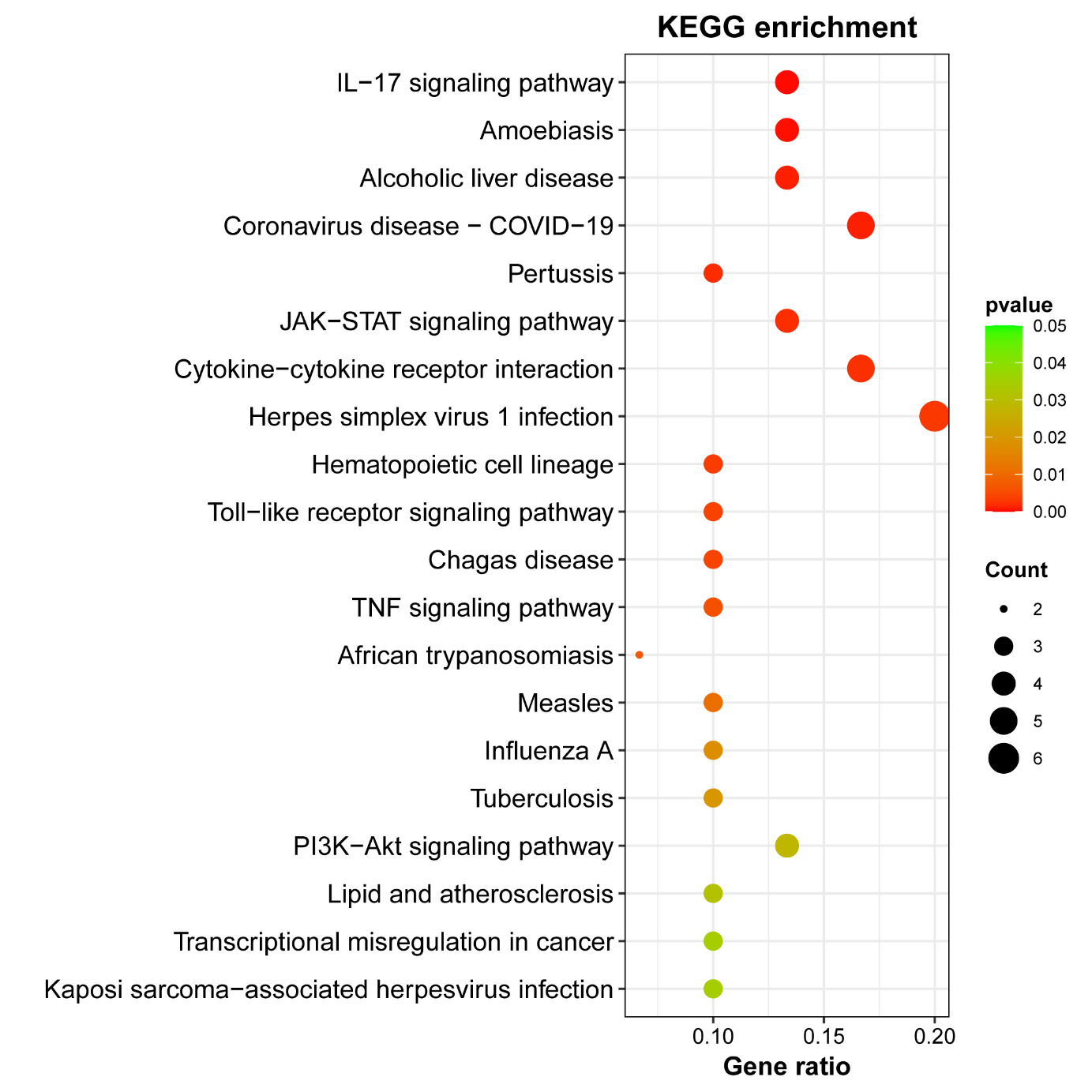
Fig. S21.** KEGG enrichment analysis of the biological functions related to DEGs.


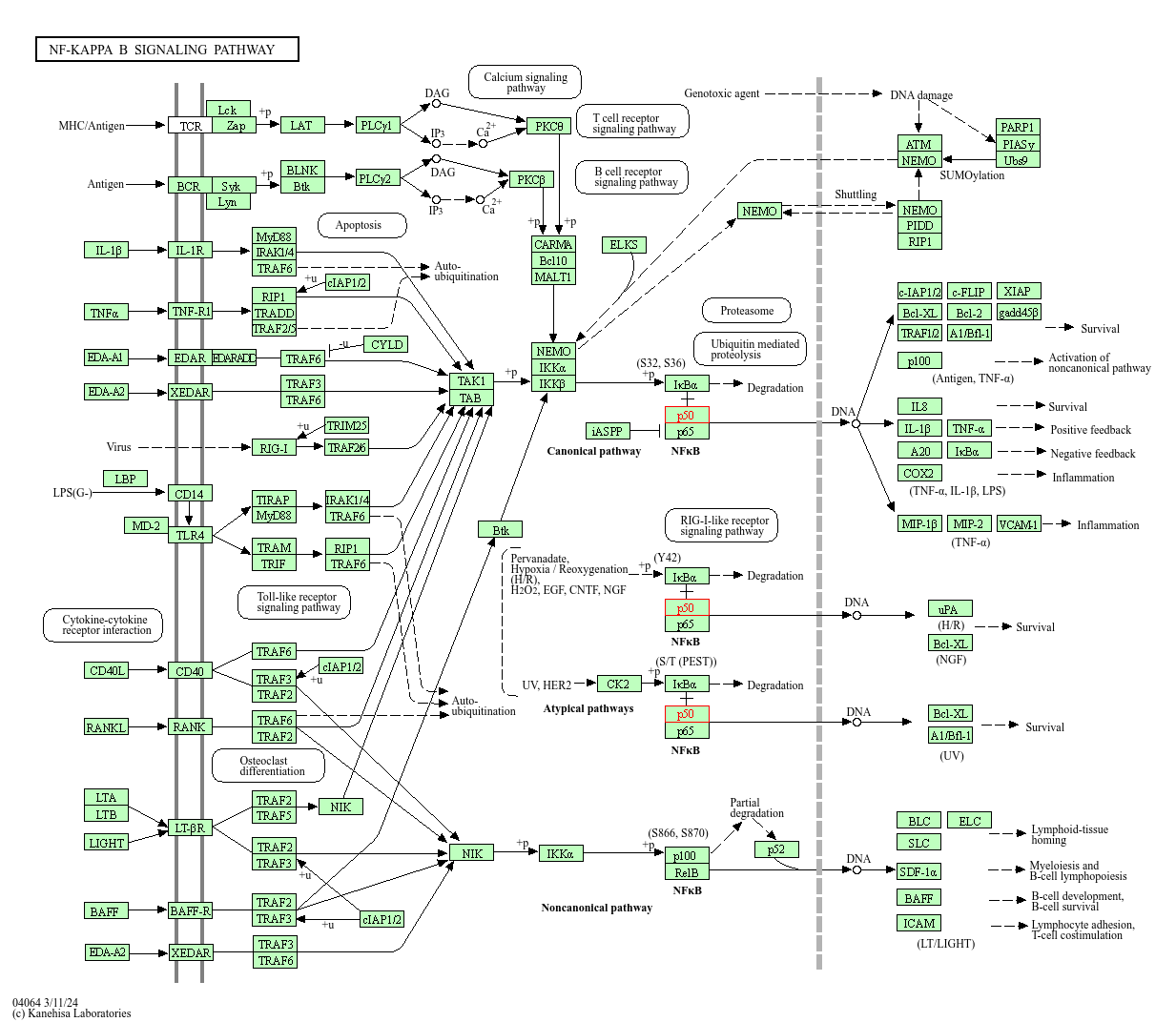


**Fig. S22.** NF-κB signaling pathway retrieved from KEGG database.


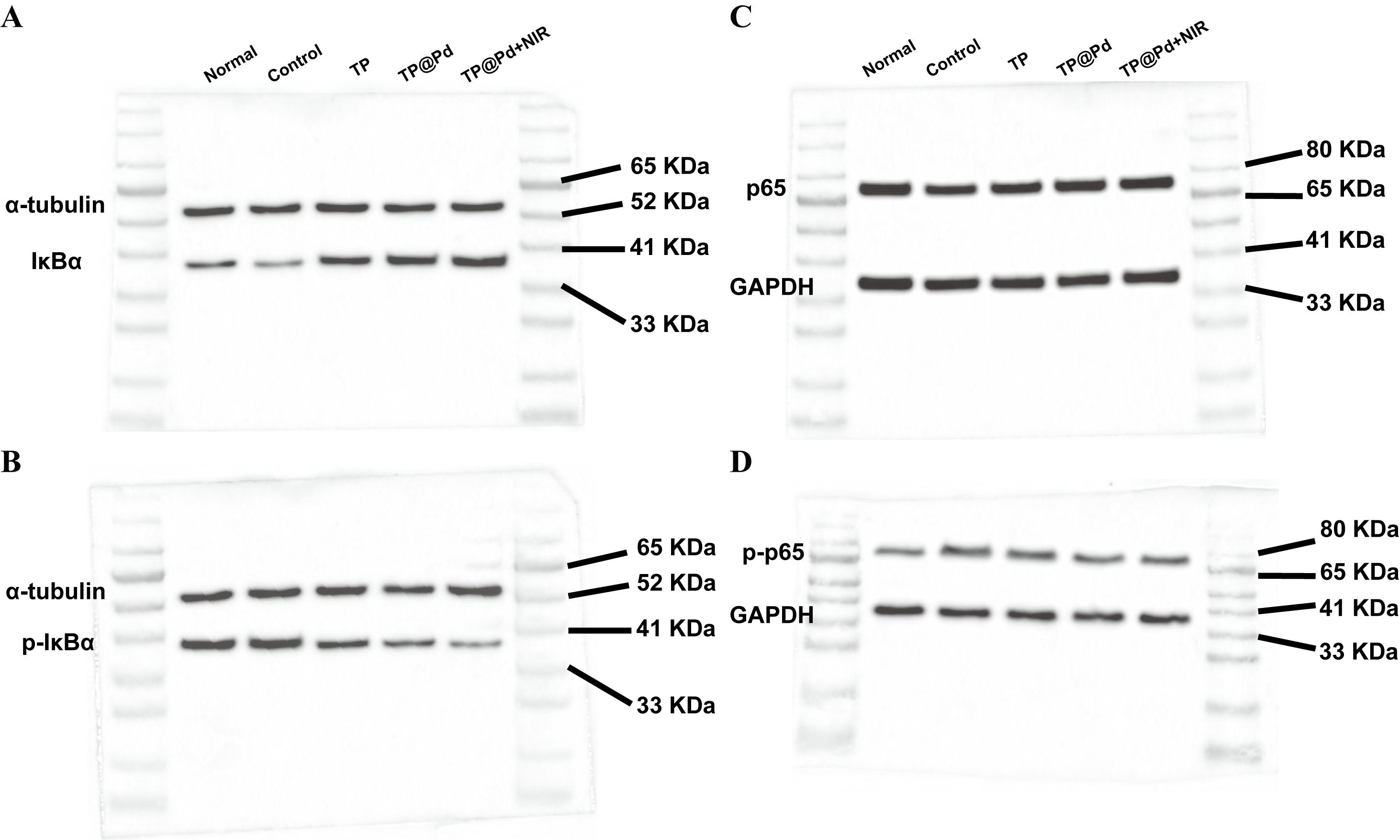


**Fig. S23.** Original images of relative proteins expression levels of treated cells by WB: IκBα (A), p-IκBα (B), p65 (C) and p-p65 (D). The corresponding groups were: cells without treatment (normal group), cells pre-treated with LPS followed by incubating with PBS (control group), cells pre-treated with LPS followed by incubating with 200 μg/mL TP (TP), cells pre-treated with LPS followed by incubating with 200 μg/mL TP@Pd (TP@Pd), and cells pre-treated with LPS followed by incubating with 200 μg/mL TP@Pd and NIR irradiation (1 W/cm^2^) (TP@Pd+NIR).


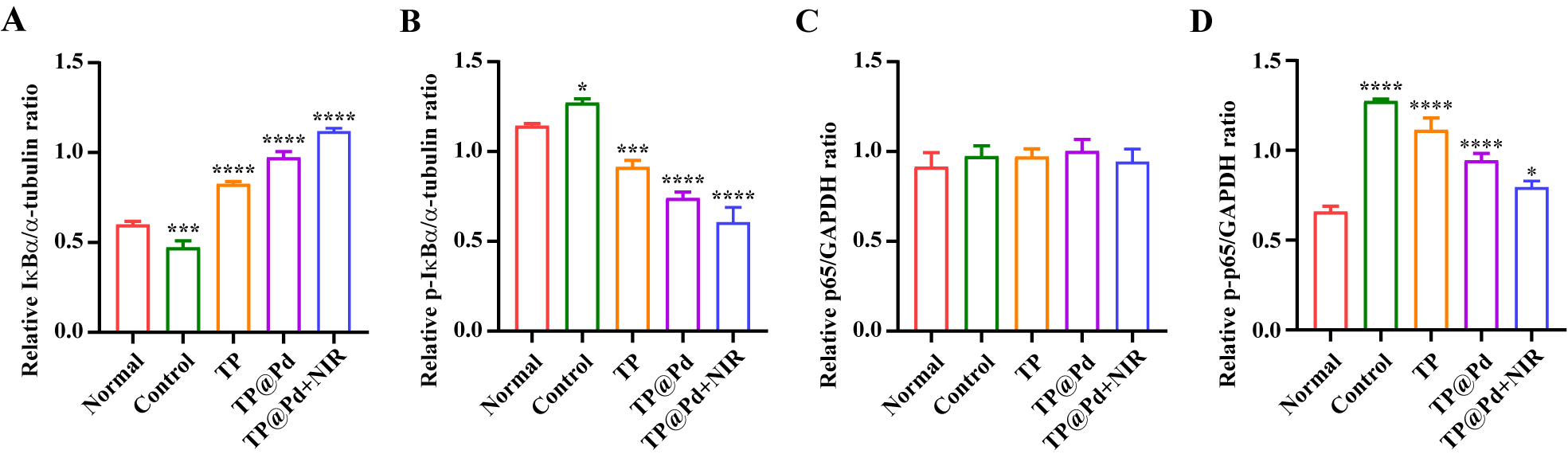


**Fig. S24.** Quantified results of relative proteins expression levels of treated cells by WB: IκBα (A), p-IκBα (B), p65 (C) and p-p65 (D). The corresponding groups were: cells without treatment (normal group), cells pre-treated with LPS followed by incubating with PBS (control group), cells pre-treated with LPS followed by incubating with 200 μg/mL TP (TP), cells pre-treated with LPS followed by incubating with 200 μg/mL TP@Pd (TP@Pd), and cells pre-treated with LPS followed by incubating with 200 μg/mL TP@Pd and NIR irradiation (1 W/cm^2^) (TP@Pd+NIR). (“*” symbol compared with normal group, *p<0.05, **p<0.01, ***p<0.001 and ****p<0.0001)


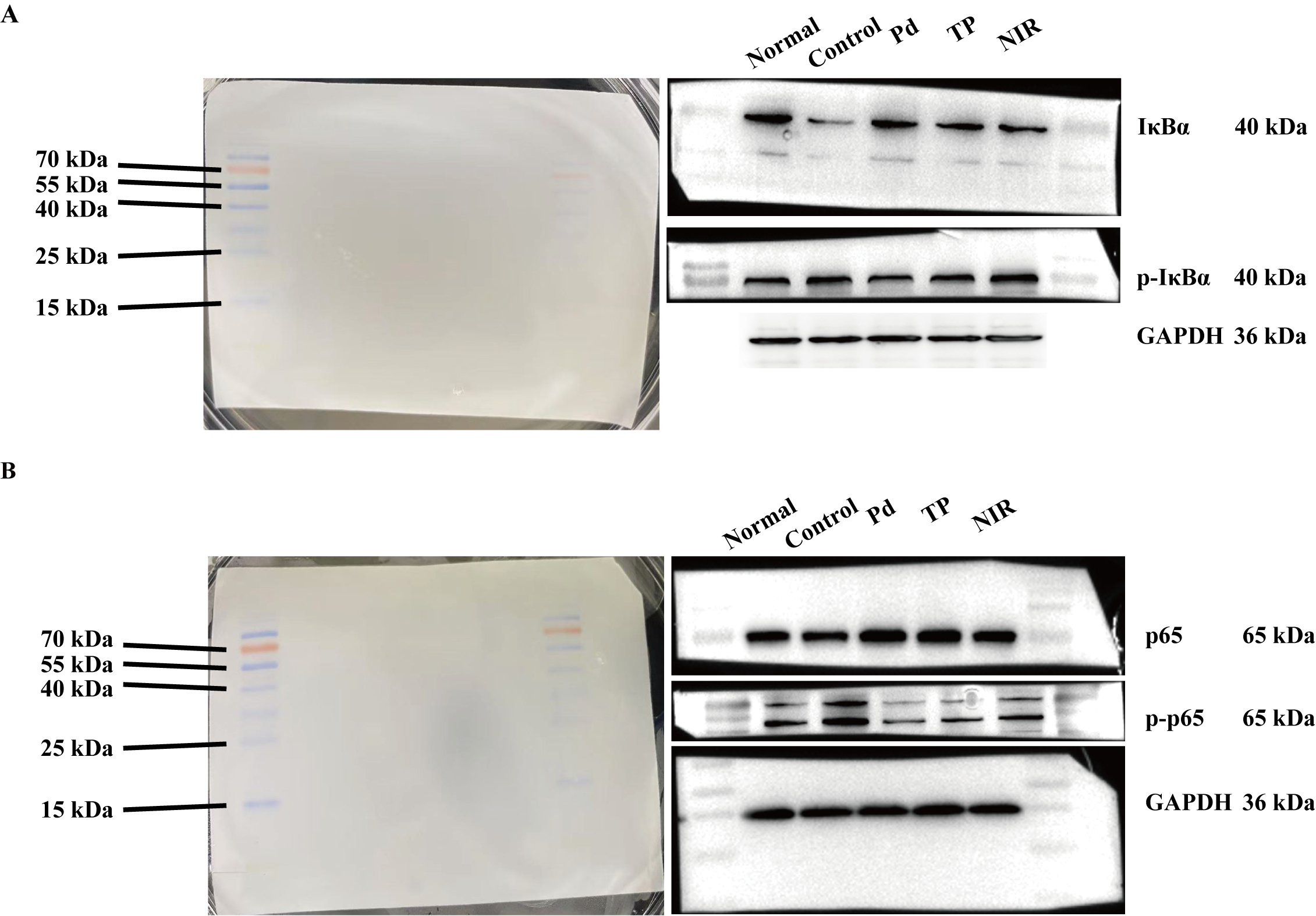


**Fig. S25.** Original images of relative proteins expression levels of treated cells by WB: IκBα and p-IκBα (A), and p65 and p-p65 (B). The corresponding groups were: cells without treatment (normal group), cells pre-treated with LPS followed by incubating with PBS (control group), cells pre-treated with LPS followed by incubating with 200 μg/mL Pd (Pd), cells pre-treated with LPS followed by incubating with 200 μg/mL TP (TP), and cells pre-treated with LPS followed by NIR irradiation alone (1 W/cm^2^) (NIR).


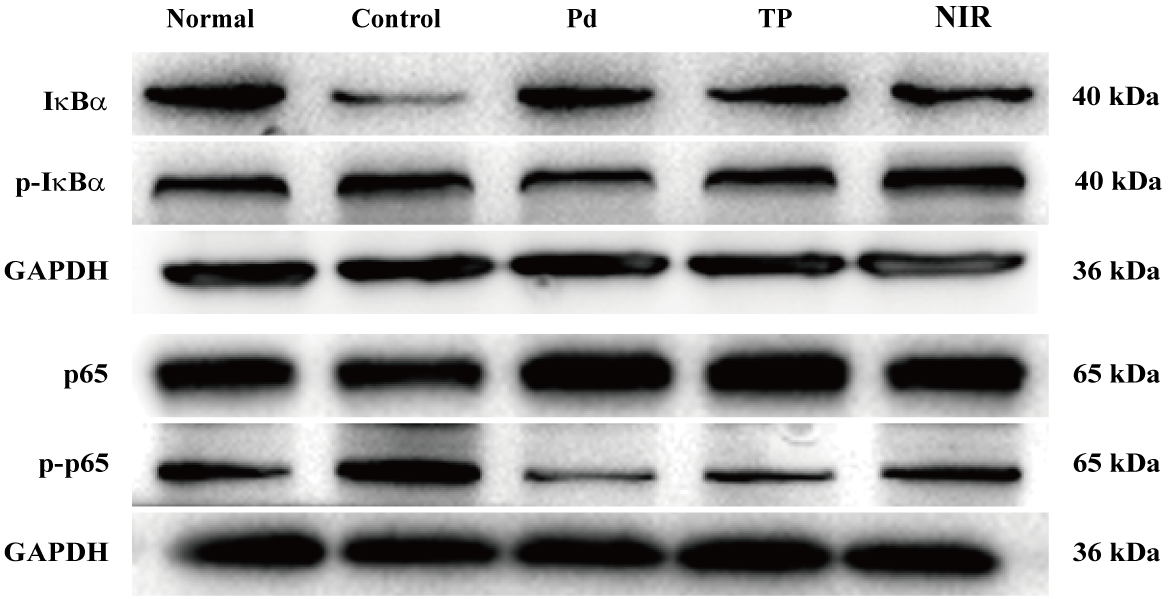


**Fig. S26.** The relative proteins expression levels of treated cells by WB. The corresponding groups were: cells without treatment (normal group), cells pre-treated with LPS followed by incubating with PBS (control group), cells pre-treated with LPS followed by incubating with 200 μg/mL Pd (Pd), cells pre-treated with LPS followed by incubating with 200 μg/mL TP (TP), and cells pre-treated with LPS followed by NIR irradiation alone (1 W/cm^2^) (NIR).

**
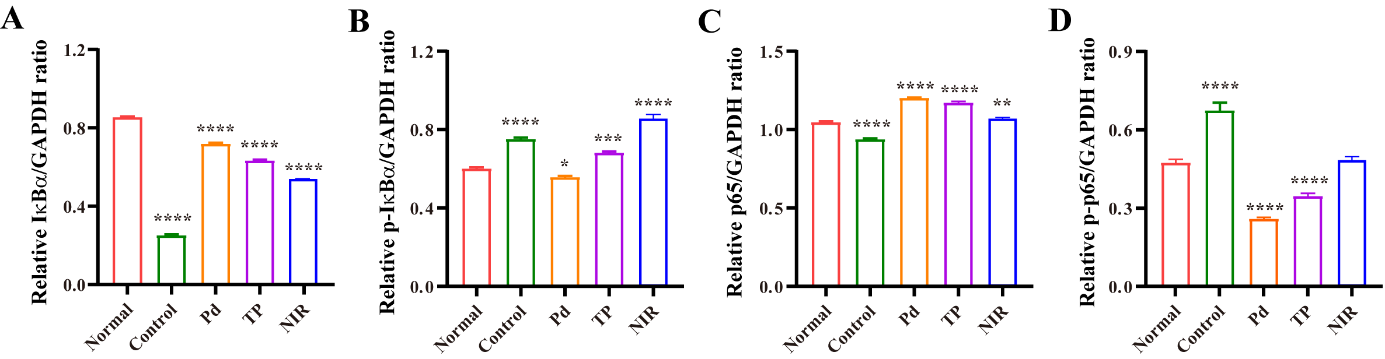
**

**Fig. S27.** Quantified results of relative proteins expression levels of treated cells by WB: IκBα (A), p-IκBα (B), p65 (C) and p-p65 (D). The corresponding groups were: cells without treatment (normal group), cells pre-treated with LPS followed by incubating with PBS (control group), cells pre-treated with LPS followed by incubating with 200 μg/mL Pd (Pd), cells pre-treated with LPS followed by incubating with 200 μg/mL TP (TP), and cells pre-treated with LPS followed by NIR irradiation alone (1 W/cm^2^) (NIR). (“*” symbol compared with normal group, *p<0.05, **p<0.01, ***p<0.001 and ****p<0.0001)


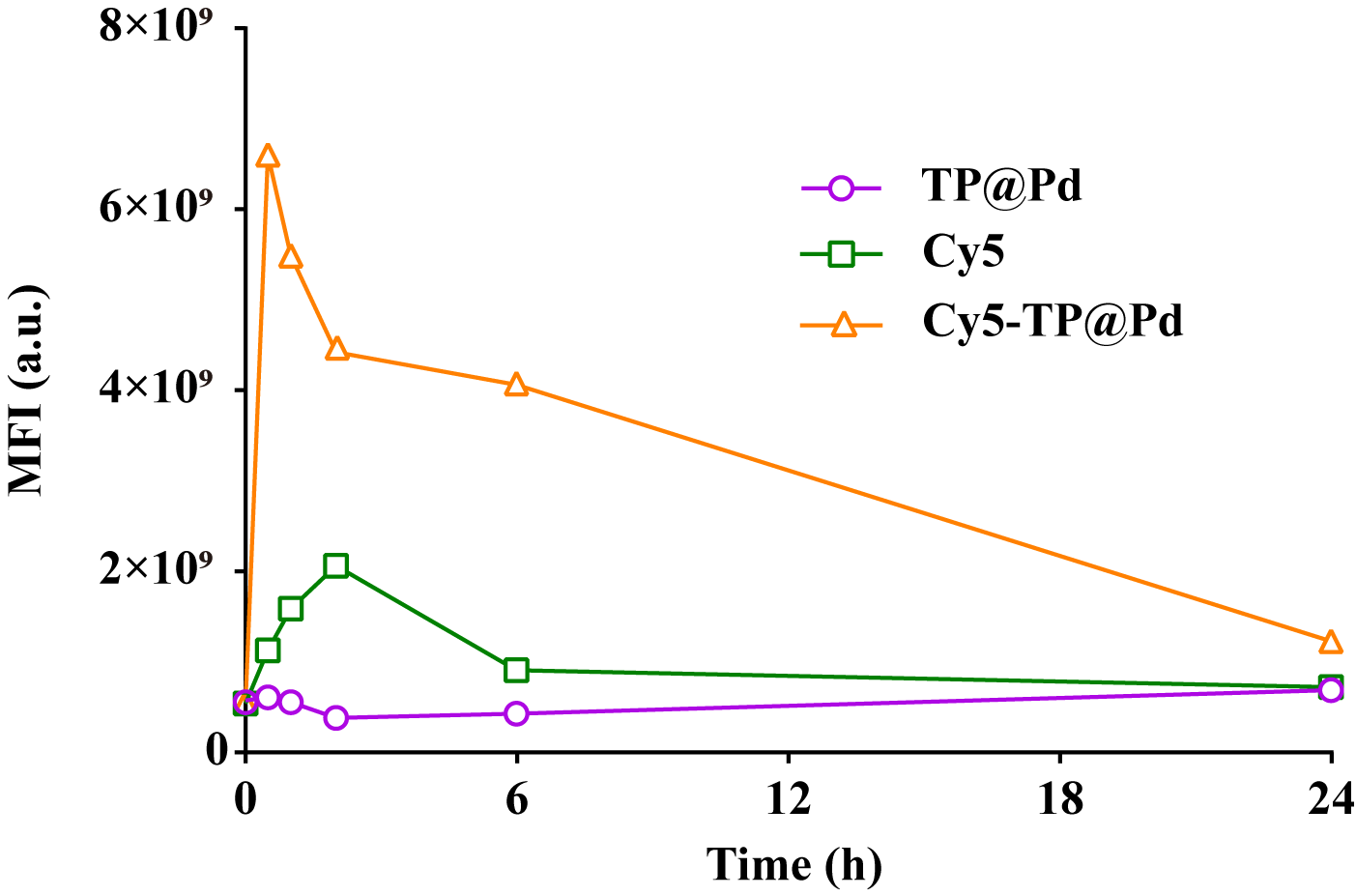


**Fig. S28.** Quantified fluorescent intensity of *in vivo* biodistribution in lung tissue by IVIS. The corresponding groups were: rats with TP@Pd (TP@Pd), Cy5 (Cy5), and Cy5-TP@Pd injection (Cy5-TP@Pd).


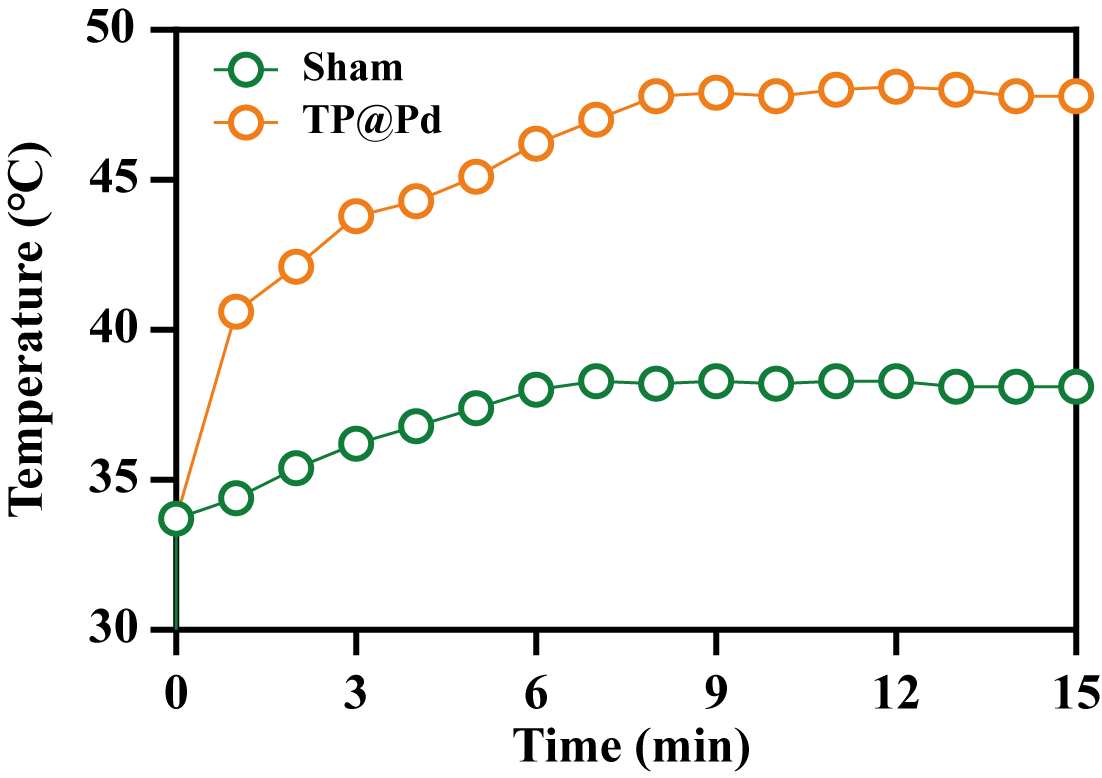


**Fig. S29.** Temperature changes of treated rats versus time under NIR irradiation (1 W/cm^2^). The corresponding groups were: rats with saline (sham group) and TP@Pd injection (TP@Pd).


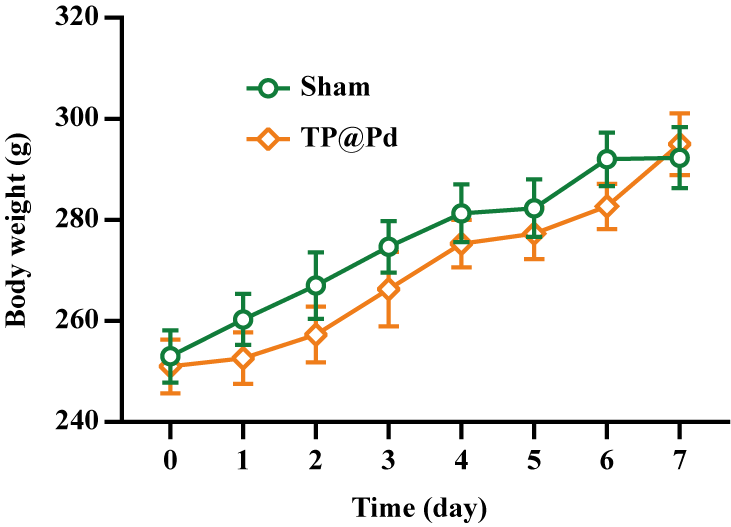


**Fig. S30.** The body weight change of treated rats versus time. The corresponding groups were: rats with saline (sham group), and TP@Pd injection (TP@Pd).

**
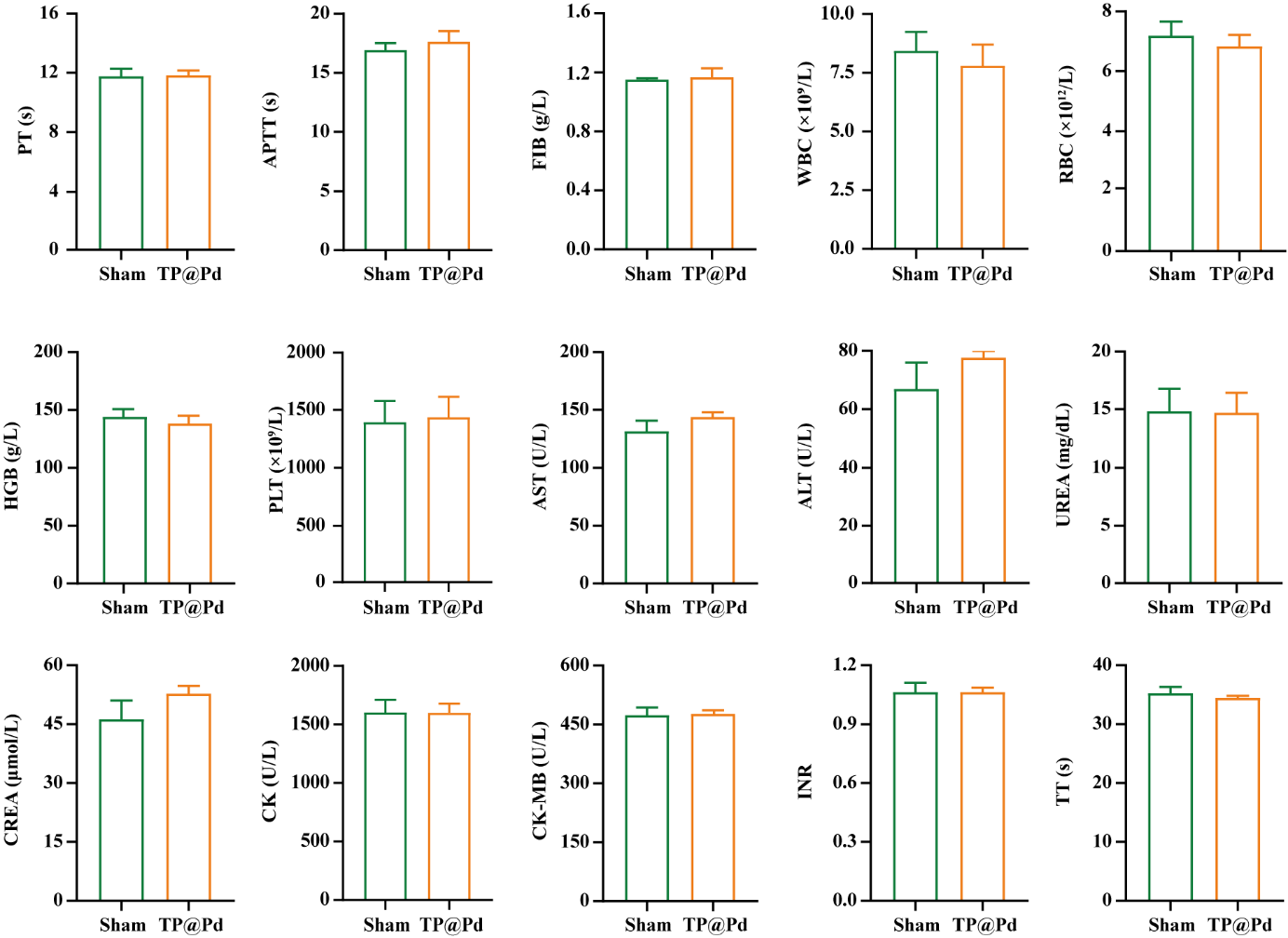
Fig. S31.** Blood indicators of treated rats after 7 days. The corresponding groups were: rats with saline (sham group) and TP@Pd injection (TP@Pd).


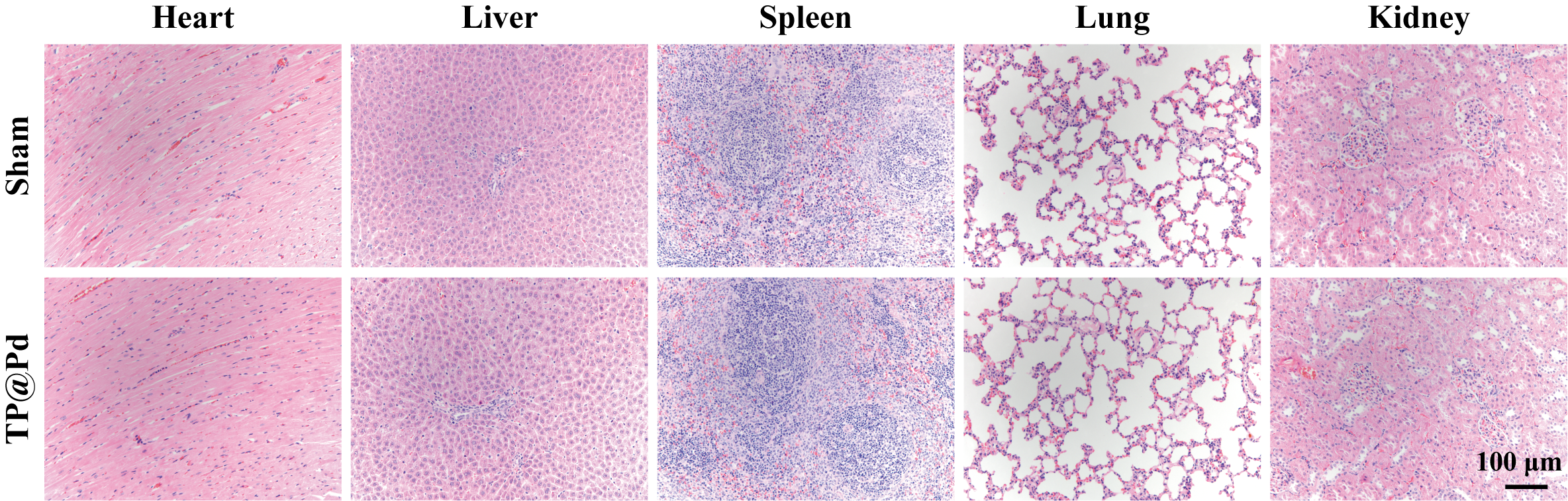


**Fig. S32.** H&E staining images of major organs (heart, liver, spleen, lung and kidney) of treated rats after 7 days. The corresponding groups were: rats with saline (sham group) and TP@Pd injection (TP@Pd).

**
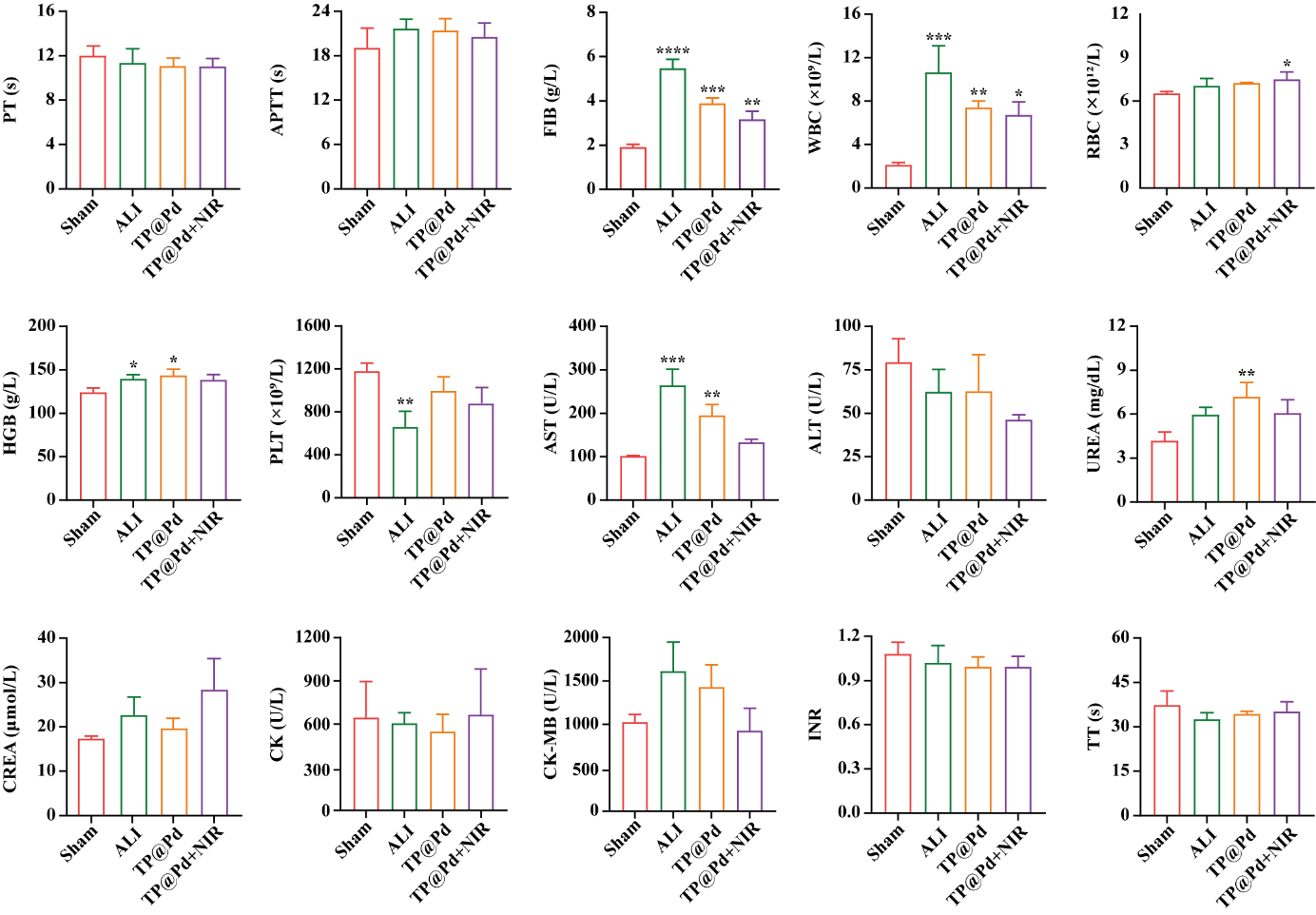
**

**Fig. S33.** Blood indicators of treated rats. The corresponding groups were: rats without treatment (sham group), LPS induced rats with saline injection (ALI group), LPS induced rats with TP@Pd injection (TP@Pd), and LPS induced rats with TP@Pd injection combining with NIR irradiation (TP@Pd+NIR). (“*” symbol compared with sham group, *p<0.05, **p<0.01, ***p<0.001 and ****p<0.0001)


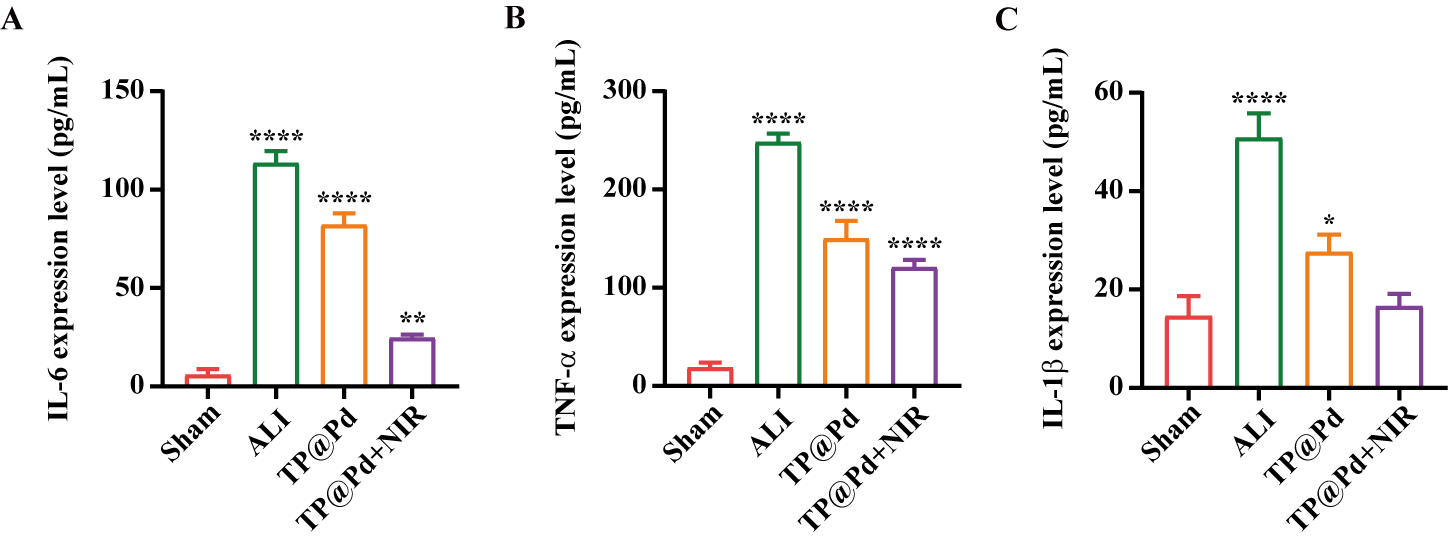


**Fig. S34.** Inflammatory factors (IL-6 (A), TNF-α (B), and IL-1β (C)) expression levels of blood serum of treated rats by ELISA. The corresponding groups were: rats without treatment (sham group), LPS induced rats with saline injection (ALI group), LPS induced rats with TP@Pd injection (TP@Pd), and LPS induced rats with TP@Pd injection combining with NIR irradiation (TP@Pd+NIR). (“*” symbol compared with sham group, *p<0.05, **p<0.01, ***p<0.001 and ****p<0.0001)


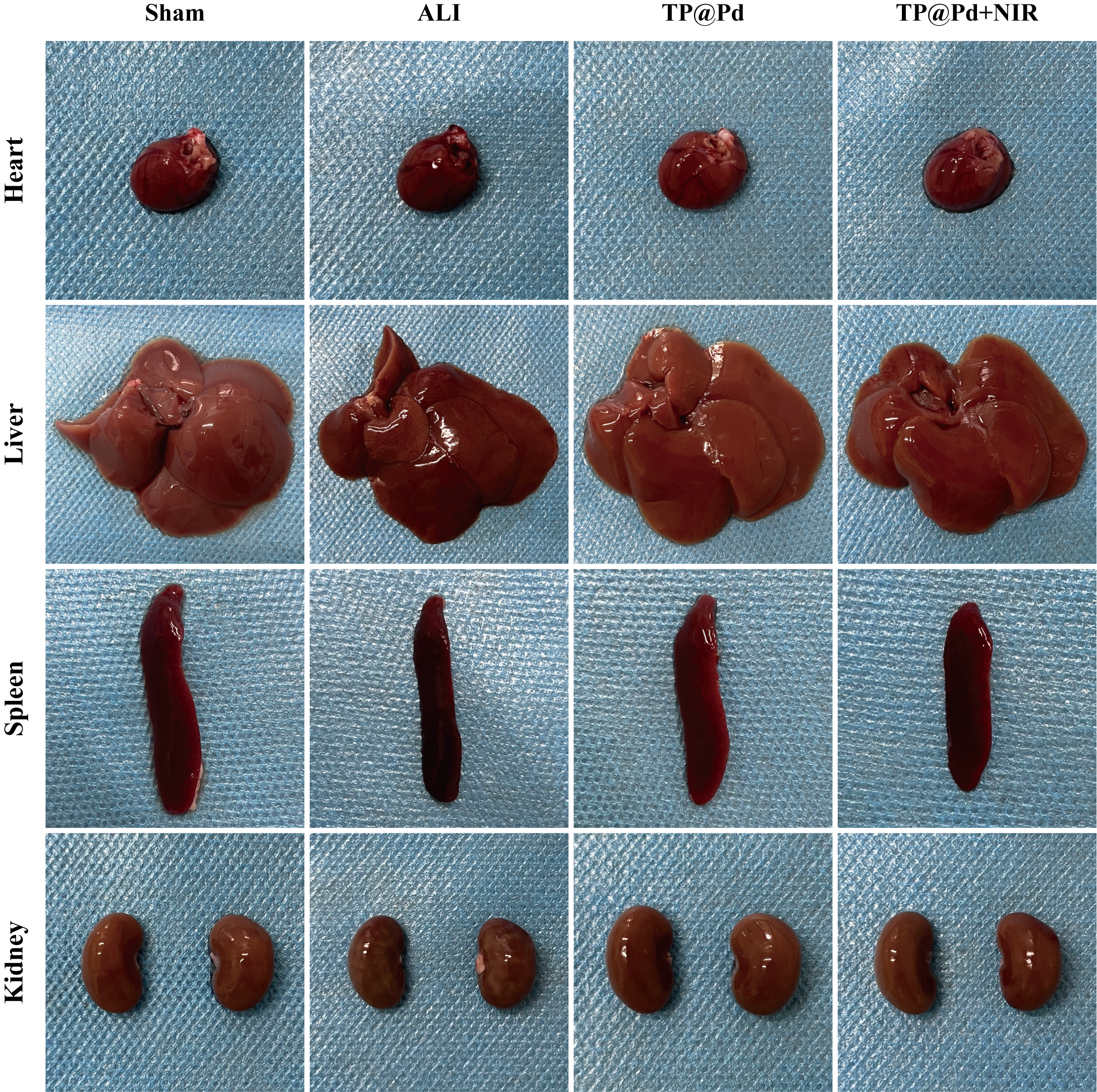


**Fig. S35.** Macroscopic observation of other organs (heart, liver, spleen and kidney) of treated rats. The corresponding groups were: rats without treatment (sham group), LPS induced rats with saline injection (ALI group), LPS induced rats with TP@Pd injection (TP@Pd), and LPS induced rats with TP@Pd injection combining with NIR irradiation (TP@Pd+NIR).


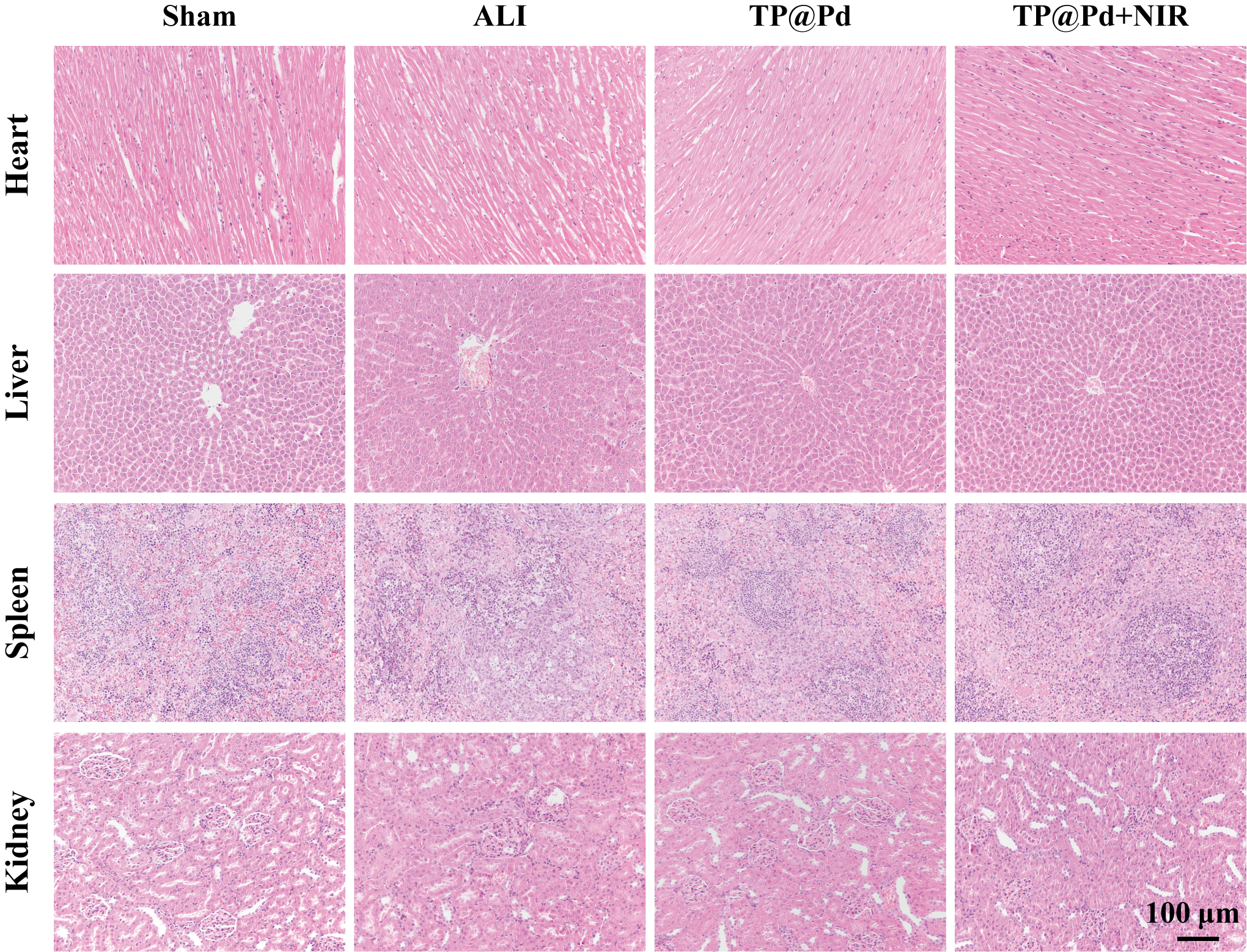


**Fig. S36.** H&E staining images of other tissues (heart, liver, spleen and kidney) of treated rats. The corresponding groups were: rats without treatment (sham group), LPS induced rats with saline injection (ALI group), LPS induced rats with TP@Pd injection (TP@Pd), and LPS induced rats with TP@Pd injection combining with NIR irradiation (TP@Pd+NIR).


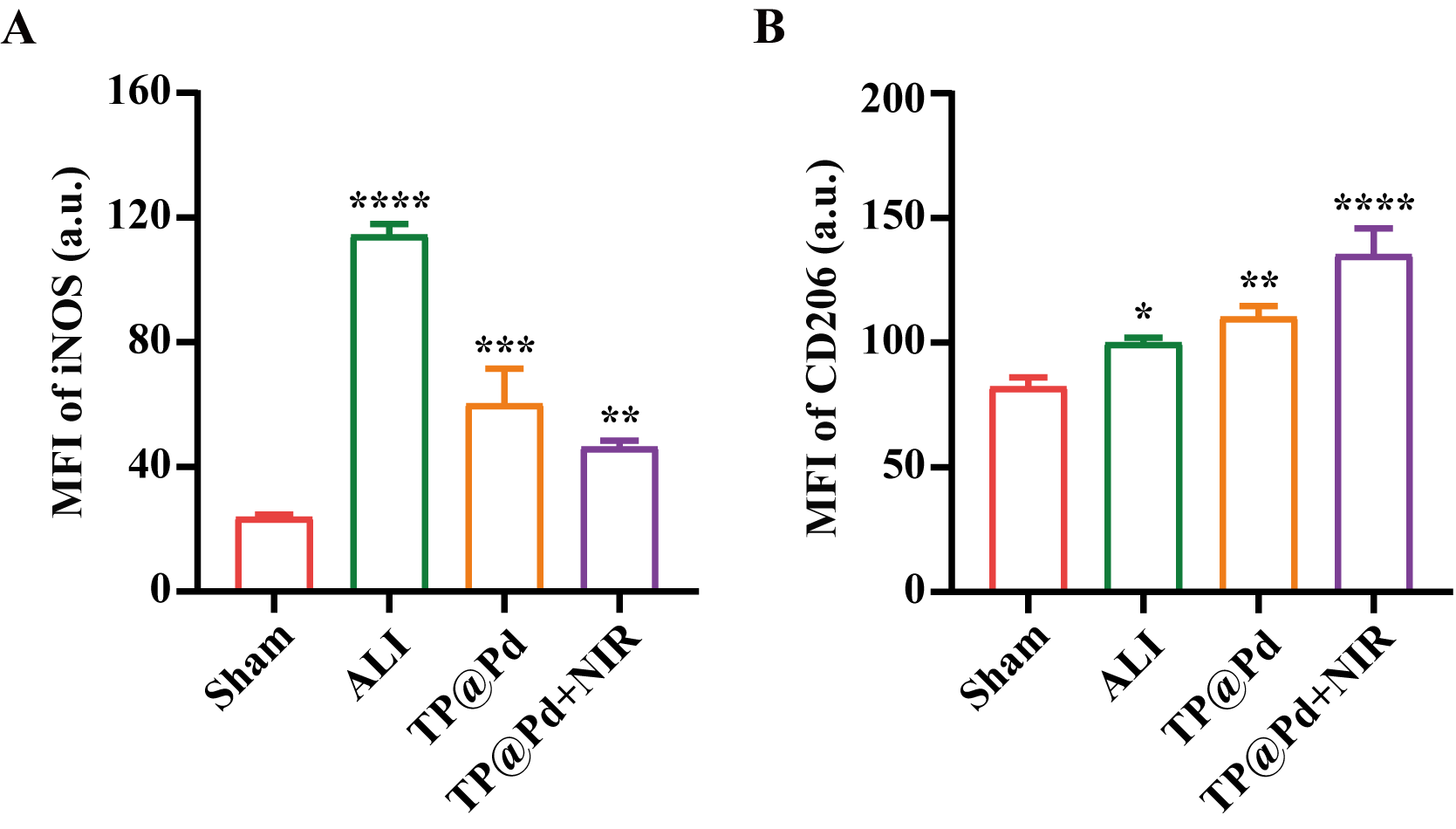


**Fig. S37.** Quantified results of iNOS and CD206 co-immunostaining images of lung tissue of treated rats. The corresponding groups were: rats without treatment (sham group), LPS induced rats with saline injection (ALI group), LPS induced rats with TP@Pd injection (TP@Pd), and LPS induced rats with TP@Pd injection combining with NIR irradiation (TP@Pd+NIR). (“*” symbol compared with sham group, *p<0.05, **p<0.01, ***p<0.001 and ****p<0.0001)


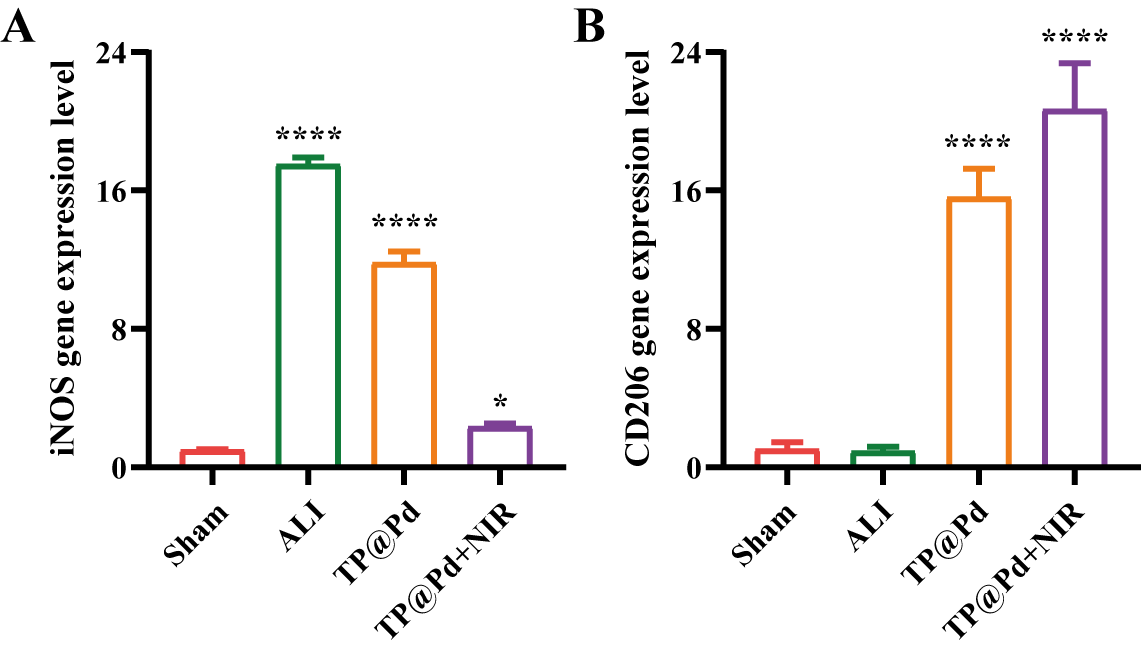


**Fig. S38.** iNOS and CD206 gene expression levels of lung tissue of treated rats by RT-qPCR. The corresponding groups were: rats without treatment (sham group), LPS induced rats with saline injection (ALI group), LPS induced rats with TP@Pd injection (TP@Pd), and LPS induced rats with TP@Pd injection combining with NIR irradiation (TP@Pd+NIR). (“*” symbol compared with sham group, *p<0.05, **p<0.01, ***p<0.001 and ****p<0.0001)


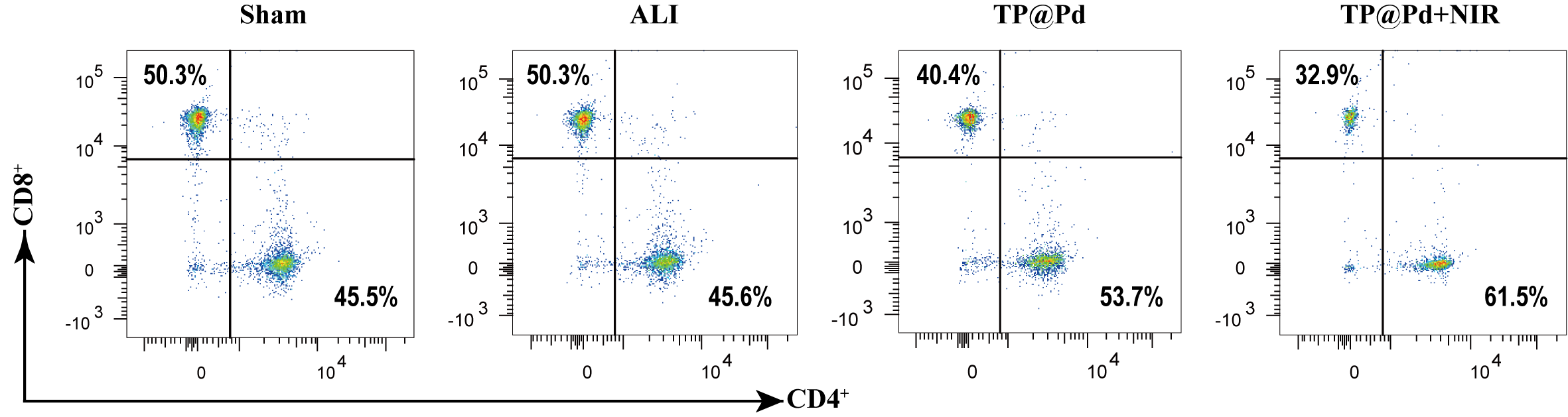


**Fig. S39.** Gating of the number of CD4^+^ and CD8^+^ T cells in the blood of treated rats by flow cytometry. The corresponding groups were: rats without treatment (sham group), LPS induced rats with saline injection (ALI group), LPS induced rats with TP@Pd injection (TP@Pd), and LPS induced rats with TP@Pd injection combining with NIR irradiation (TP@Pd+NIR).


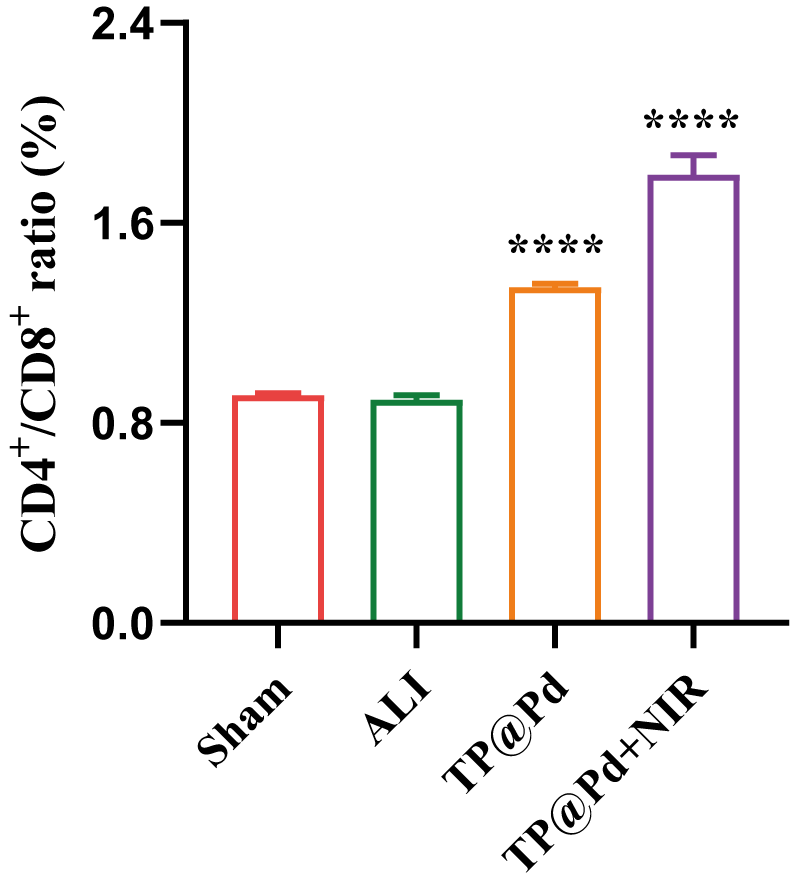


**Fig. S40.** CD4^+^/CD8^+^ ratio in the blood of treated rats by flow cytometry. The corresponding groups were: rats without treatment (sham group), LPS induced rats with saline injection (ALI group), LPS induced rats with TP@Pd injection (TP@Pd), and LPS induced rats with TP@Pd injection combining with NIR irradiation (TP@Pd+NIR). (“*” symbol compared with sham group, *p<0.05, **p<0.01, ***p<0.001 and ****p<0.0001)


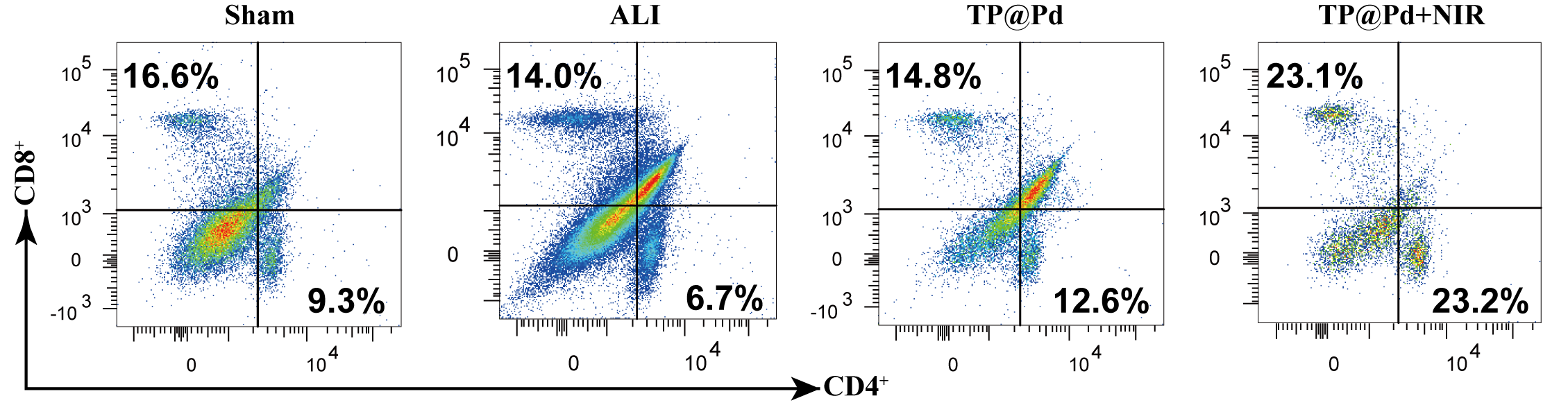


**Fig. S41.** Gating of the number of CD4^+^ and CD8^+^ T cells in lung tissue of treated rats by flow cytometry. The corresponding groups were: rats without treatment (sham group), LPS induced rats with saline injection (ALI group), LPS induced rats with TP@Pd injection (TP@Pd), and LPS induced rats with TP@Pd injection combining with NIR irradiation (TP@Pd+NIR).


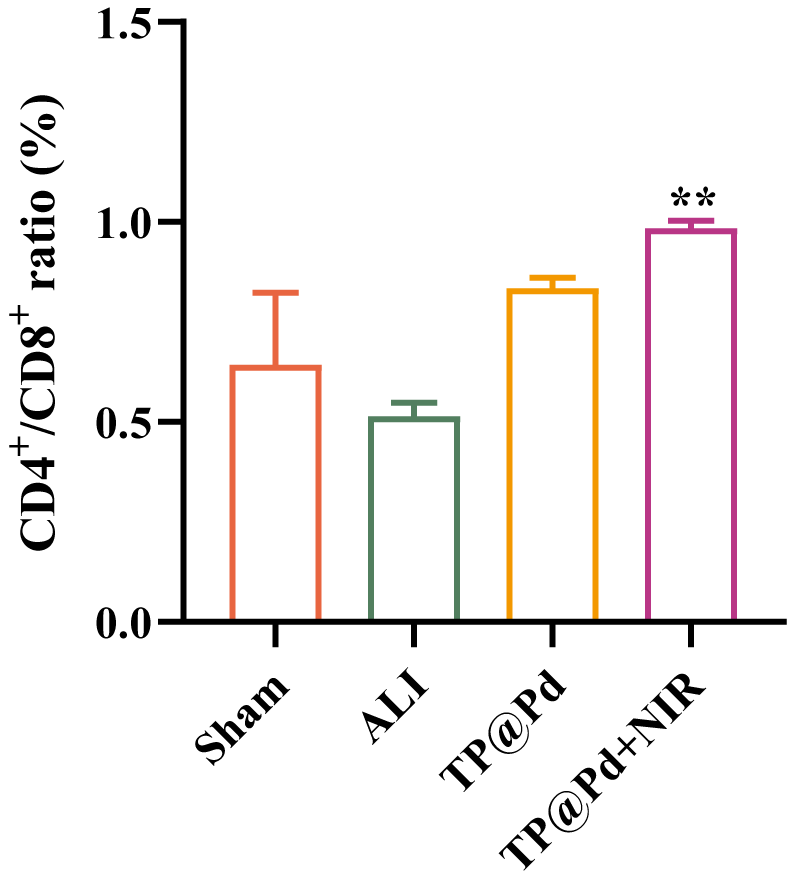


**Fig. S42.** CD4^+^/CD8^+^ ratio in lung tissue of treated rats by flow cytometry. The corresponding groups were: rats without treatment (sham group), LPS induced rats with saline injection (ALI group), LPS induced rats with TP@Pd injection (TP@Pd), and LPS induced rats with TP@Pd injection combining with NIR irradiation (TP@Pd+NIR). (“*” symbol compared with sham group, *p<0.05, **p<0.01, ***p<0.001 and ****p<0.0001)


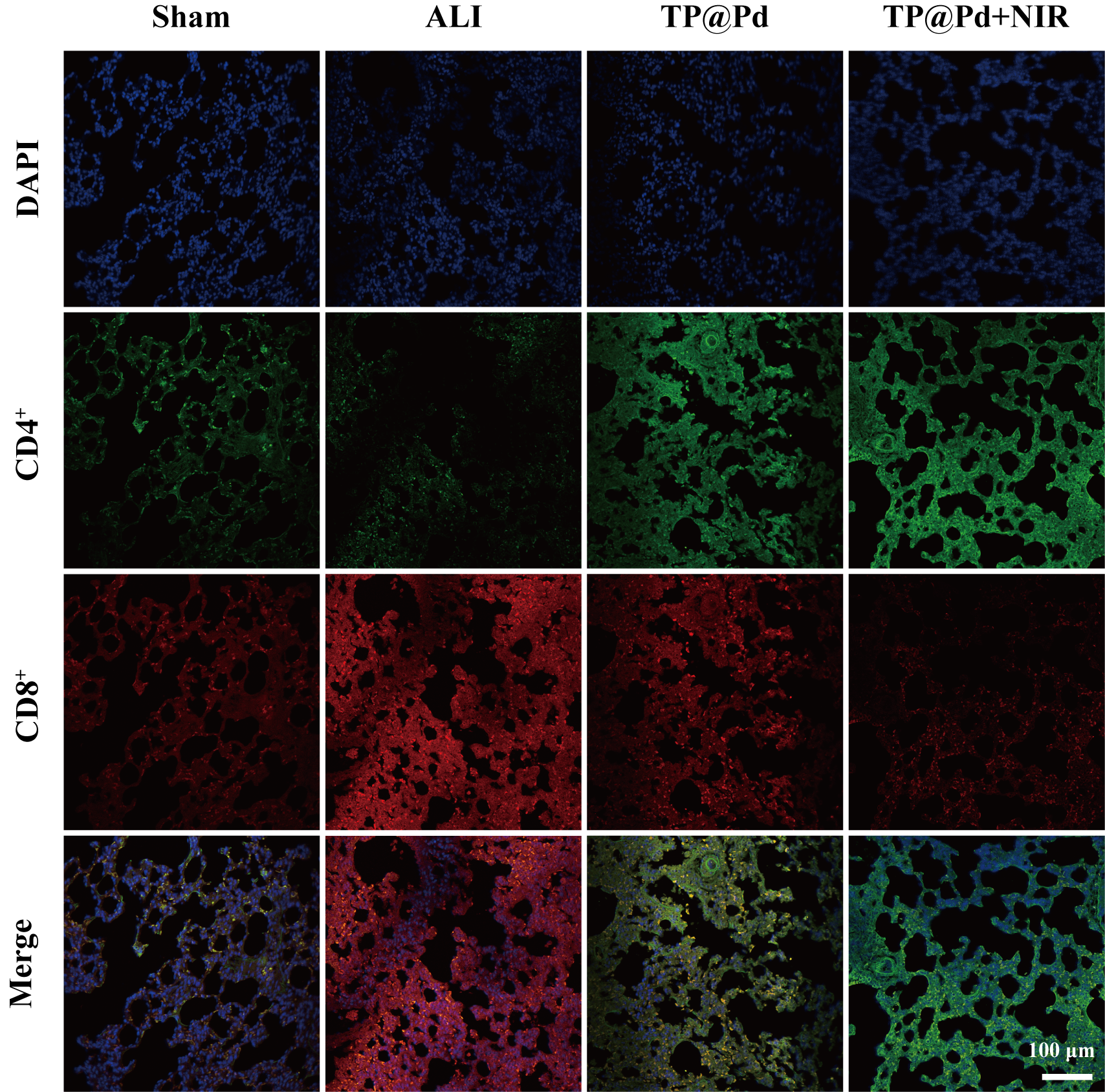


**Fig. S43.** CD4^+^ and CD8^+^ T cells co-immunofluorescent staining images in lung tissue of treated rats by fluorescent microscopy. The corresponding groups were: rats without treatment (sham group), LPS induced rats with saline injection (ALI group), LPS induced rats with TP@Pd injection (TP@Pd), and LPS induced rats with TP@Pd injection combining with NIR irradiation (TP@Pd+NIR).


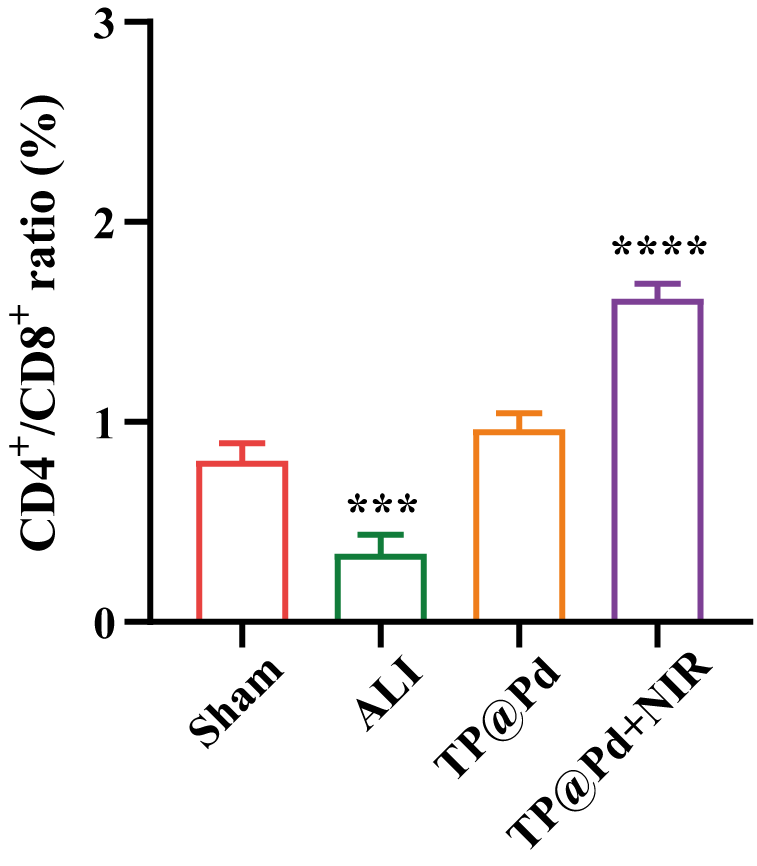


**Fig. S44.** CD4^+^/CD8^+^ ratio in lung tissue of treated rats. The corresponding groups were: rats without treatment (sham group), LPS induced rats with saline injection (ALI group), LPS induced rats with TP@Pd injection (TP@Pd), and LPS induced rats with TP@Pd injection combining with NIR irradiation (TP@Pd+NIR). (“*” symbol compared with sham group, *p<0.05, **p<0.01, ***p<0.001 and ****p<0.0001)


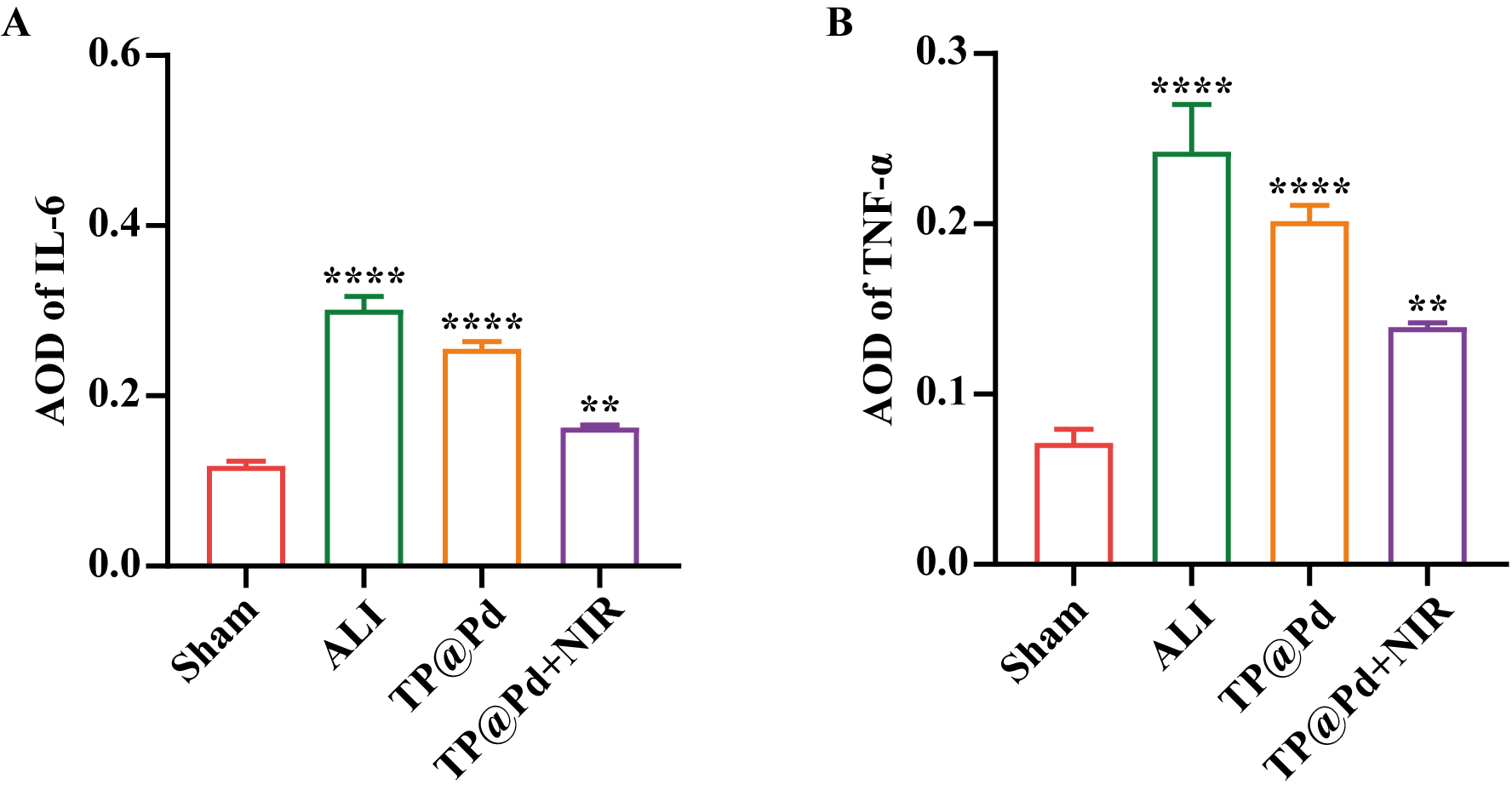


**Fig. S45.** Quantified results of IL-6 and TNF-α expression levels of lung tissue of treated rats. The corresponding groups were: rats without treatment (sham group), LPS induced rats with saline injection (ALI group), LPS induced rats with TP@Pd injection (TP@Pd), and LPS induced rats with TP@Pd injection combining with NIR irradiation (TP@Pd+NIR). (“*” symbol compared with normal group, *p<0.05, **p<0.01, ***p<0.001 and ****p<0.0001)


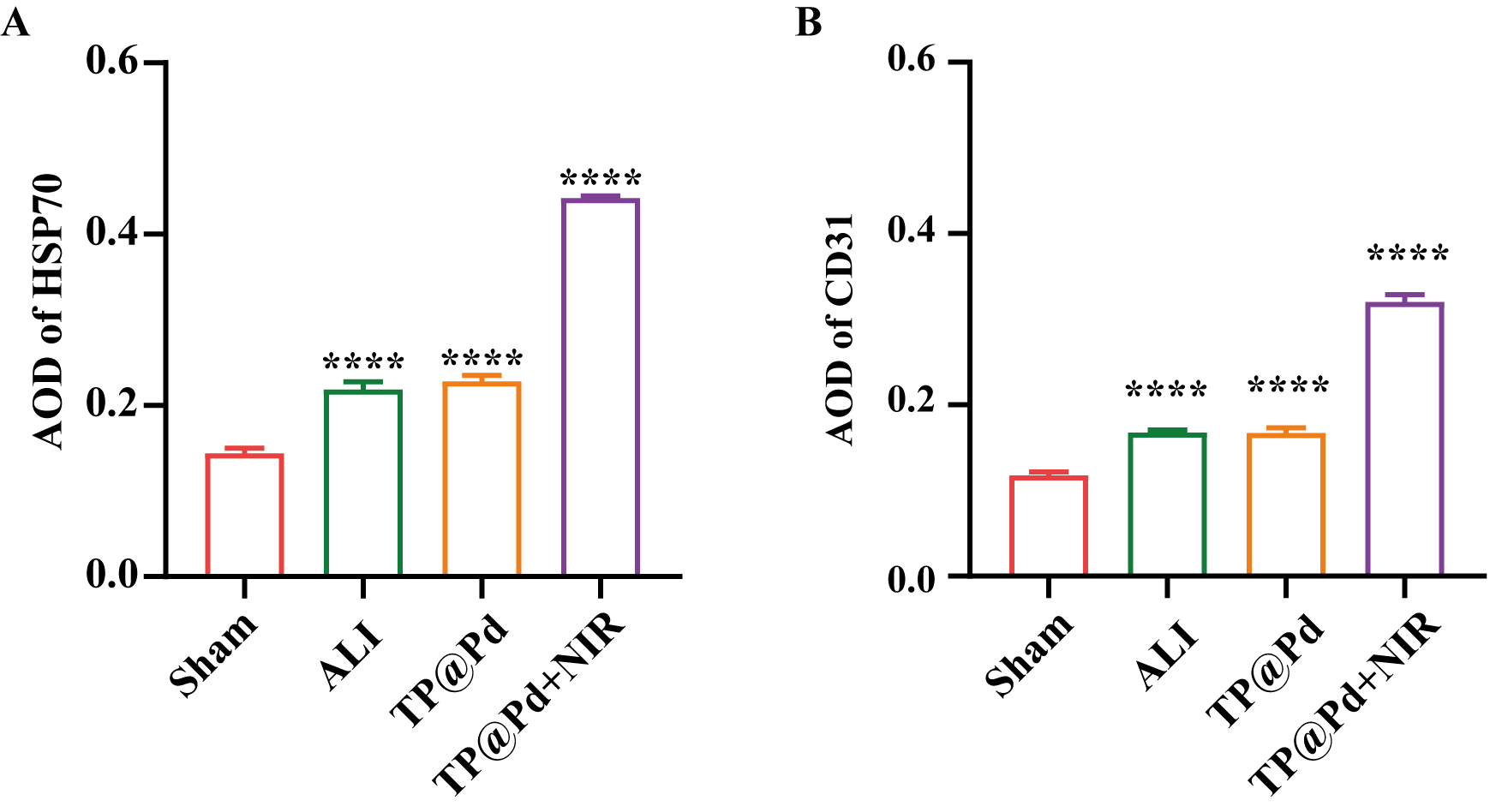


**Fig. S46.** Quantified results of HSP70 and CD31 expression levels of lung tissue of treated rats. The corresponding groups were: rats without treatment (sham group), LPS induced rats with saline injection (ALI group), LPS induced rats with TP@Pd injection (TP@Pd), and LPS induced rats with TP@Pd injection combining with NIR irradiation (TP@Pd+NIR). (“*” symbol compared with normal group, *p<0.05, **p<0.01, ***p<0.001 and ****p<0.0001)

Table S1. Reaction recipe of TP and TP@Pd and their corresponding diameter by TEM, zeta potential by zeta sizer, and weight loss by TGA.

| Name | TAPA (mg) | PA  (mg) | TP  (mg) | Na_2_PdCl_4_ (mg) | Diameter (nm) | Zeta potential (mV) | Weight remaining ratio (%) |
| --- | --- | --- | --- | --- | --- | --- | --- |
| TP | 60 | 30 | / | / | 216.15±17.16 | 32.63±1.03 | 47.44 |
| TP@Pd | / | / | 50 | 50 | 231.97±31.69 | -18.50±0.46 | 64.55 |

Table S2. Element composition of TP and TP@Pd by XPS and ICP-OES.

| Name | XPS | | | | ICP-OES |
| --- | --- | --- | --- | --- | --- |
|  | C (%) | N (%) | O (%) | Pd (%) | Pd (%) |
| TP | 80.42 | 7.86 | 11.72 | / | / |
| TP@Pd | 68.36 | 6.91 | 22.87 | 1.86 | 8.60±0.21 |

Table S3. The surface adsorption properties of TP and TP@Pd by BET.

| Sample | Pore size (nm) | Pore volume (cm^3^/g) | Surface area (m^2^/g) |
| --- | --- | --- | --- |
| TP | 16.85 | 0.63 | 161.32 |
| TP@Pd | 12.14 | 0.51 | 192.30 |

Table S4. ROS scavenging ratio of TP and TP@Pd with the concentration of 200 μg/mL.

| Name | TP (%) | TP@Pd (%) | TP@Pd +NIR (%) |
| --- | --- | --- | --- |
| H_2_O_2_ | 27.74±0.75 | 59.75±0.48 | 69.30±0.26 |
| ·OH | 12.45±0.93 | 38.73±3.75 | 64.00±2.93 |
| ·O_2_^-^ | 25.69±0.77 | 46.62±1.86 | 66.90±0.51 |

Table S5. ROS scavenging ratio of TP@Pd with different concentrations.

| Name | 50 μg/mL (%) | 100 μg/mL (%) | 200 μg/mL (%) | 500 μg/mL (%) |
| --- | --- | --- | --- | --- |
| H_2_O_2_ | 36.32±0.84 | 41.80±0.39 | 58.84±0.49 | 84.69±0.20 |
| ·OH | 24.13±1.45 | 31.20±1.62 | 40.29±1.34 | 52.26±0.98 |
| ·O_2_^-^ | 22.08±1.83 | 30.25±1.46 | 45.02±1.39 | 73.79±0.63 |

Table S6. ROS scavenging ratio of TP and Pd with the concentration of 200 μg/mL, and NIR irradiation alone for 10 min.

| Name | TP (%) | Pd (%) | NIR (%) |
| --- | --- | --- | --- |
| H_2_O_2_ | 28.68±0.32 | 78.07±0.14 | 1.73±0.71 |
| ·OH | 12.62±0.95 | 32.51±1.26 | 3.43±2.18 |
| ·O_2_^-^ | 23.31±1.45 | 44.24±1.06 | 6.19±1.92 |

Table S7. Blood indicators of treated rats. The corresponding groups were: rats without treatment (Sham group) and rats with TP@Pd injection (TP@Pd) after 7 days.

| Name | Sham group | TP@Pd |
| --- | --- | --- |
| PT (s) | 11.77±0.49 | 11.83±0.32 |
| INR | 1.06±0.05 | 1.06±0.02 |
| APTT (s) | 16.93±0.58 | 17.63±0.90 |
| TT (s) | 35.23±1.10 | 34.43±0.38 |
| FIB (g/L) | 1.15±0.01 | 1.17±0.06 |
| WBC (×10^9^/L) | 8.43±0.80 | 7.80±0.90 |
| RBC (×10^12^/L) | 7.19±0.47 | 6.83±0.38 |
| HGB (g/L) | 144.00±8.66 | 137.67±7.51 |
| PLT (×10^9^/L) | 1394.33±186.51 | 1438.33±177.30 |
| ALT (U/L) | 66.89±9.09 | 77.71±2.29 |
| AST (U/L) | 131.66±9.07 | 143.86±4.04 |
| CREA (μmol/L) | 46.22±4.81 | 52.77±2.02 |
| UREA (mg/dL) | 14.84±1.96 | 14.72±1.72 |
| CK (U/L) | 1602.43±108.25 | 1600.79±77.22 |
| CK-MB (U/L) | 473.70±19.51 | 476.55±9.56 |

Table S8. Blood indicators of treated rats. The corresponding groups were: rats without treatment (sham group), LPS induced rats with PBS injection (ALI group), LPS induced rats with TP@Pd injection (TP@Pd), and LPS induced rats with TP@Pd injection and NIR irradiation (TP@Pd+NIR).

| Name | Sham | ALI | TP@Pd | TP@Pd+NIR |
| --- | --- | --- | --- | --- |
| PT (s) | 12.00±0.89 | 11.33±1.29 | 11.07±0.72 | 11.03±0.81 |
| APTT (s) | 19.07±2.67 | 21.67±1.27 | 21.43±1.57 | 20.53±1.97 |
| FIB (g/L) | 1.92±0.12 | 5.47±0.42 | 3.89±0.23 | 3.16±0.36 |
| WBC (×10^9^/L) | 2.13±0.23 | 10.63±2.46 | 7.41±0.59 | 6.73±1.21 |
| RBC (×10^12^/L) | 6.52±0.15 | 7.04±0.52 | 7.25±0.04 | 7.50±0.51 |
| HGB (g/L) | 124.10±5.04 | 139.67±4.51 | 143.33±7.23 | 138.33±6.03 |
| PLT (×10^9^/L) | 1181.10±73.23 | 661.00±146.74 | 995.67±131.93 | 882.00±145.17 |
| AST (U/L) | 101.33±1.53 | 264.00±37.24 | 195.00±25.24 | 133.33±7.02 |
| ALT (U/L) | 79.33±13.65 | 62.33±12.90 | 62.67±21.13 | 46.33±2.89 |
| UREA (mg/dL) | 4.19±0.61 | 5.95±0.51 | 7.19±0.98 | 6.06±0.92 |
| CREA (μmol/L) | 17.33±0.58 | 22.67±4.04 | 19.67±2.31 | 28.33±7.09 |
| CK (U/L) | 644.00±250.57 | 605.67±73.50 | 547.67±121.01 | 664.67±322.07 |
| CK-MB (U/L) | 1025.33±88.95 | 1611.33±333.71 | 1430.67±255.21 | 927.67±258.46 |
| INR | 1.08±0.08 | 1.02±0.12 | 0.99±0.07 | 0.99±0.07 |
| TT (s) | 37.30±4.75 | 32.57±2.21 | 34.40±0.82 | 35.23±3.26 |

Table S9. Detailed primer sequences for qRT-PCR.

| Gene | Forward sequences (5’ to 3’) | Reverse sequences (3’ to 5’) |
| --- | --- | --- |
| GAPDH | ACTTGAAGGGTGGAGCCAAA | GCCCTTCCACAATGCCAAAG |
| IL-6 | GAGAGGAGACTTCACAGAGGATACC | TCATTTCCACGATTTCCCAGAGAAC |
| IL-1β | TGCCACCTTTTGACAGTGATG | ATGTGCTGCTGCGAGATTTG |
| SOD2 | GTAGGGCCTGTCCGATGATG | CGCTACTGAGAAAGGTGCCA |
| iNOS | TGGAGCGAGTTGTGGATTGT | GTGAGGGCTTGGCTGAGTGA |
| CD86 | CAGCACGGACTTGAACAACC | CTCCACGGAAACAGCATCTGA |
| CD206 | TAGCACTGGGTTGCATTGGT | TGCAGGGTTGACATGAGACC |
| IL-10 | GAGAAGCATGGCCCAGAAATC | GAGAAATCGATGACAGCGCC |
| CD31 | AGTCAGAGTCTTCCTTGCCC | TCTGTTTGGCCTTGGCTTTC |
| HSP70 | CTTGGGCACCGATTACTGTC | ATAATCCCCTGGTACAGTGC |
